# β-catenin-driven innate and metabolic reprograming in macrophages fuel T-cell-dependent inflammation in *Toxoplasma gondii* infection: implications for therapeutic intervention

**DOI:** 10.1038/s41419-026-08953-1

**Published:** 2026-06-13

**Authors:** Geetika Kumari, Amit Kumar, Rasmiranjan Muduli, Mayami Das, Prithwik Bhowmik, Biplab Singha, Ankita Namdeo, Criss Dcosta, Neerja Wadhwa, Jaswinder Singh Maras, Rakesh Kundu, Nishith Gupta, Ruchi Anand, Dhanasekaran Shanmugam, Tanmay Majumdar

**Affiliations:** 1 National Institute of Immunology, New Delhi, India; 2 Biochemical Sciences Division, CSIR-National Chemical Laboratory, Pune, India; 3 Department of Chemistry, Indian Institute of Technology, Mumbai, India; 4 Department of Molecular and Cellular Medicine, Institute of Liver and Biliary Sciences, New Delhi, India; 5 Department of Zoology, Visva-Bharati University, Santiniketan, India; 6 Intracellular Parasite Education and Research Labs, Department of Biological Sciences, Birla Institute of Technology and Science, Hyderabad, India

**Keywords:** Inflammasome, Parasitic infection, Cell signalling, Cell death and immune response, Inflammasome

## Abstract

*Toxoplasma gondii* activates innate immunity via TLR11/12 in mice, but the lack of functional human counterparts leaves a gap in understanding parasite sensing in humans. Here, we bridge this gap by uncovering a host-intrinsic sensing mechanism, wherein β-catenin signaling mediates immune recognition of *T. gondii*. Notably, this parasite hijacks the PI3K-AKT-β-catenin pathway in macrophages to promote its replication. While β-catenin ablation, either genetically or pharmacologically (XAV939), disavows this process, thereby inhibiting replication. Phospho-β-catenin-TCF4 drives IRF4 transcription, followed by phosphorylation of IRF4, which regulates CYBB transcription. Augmented CYBB enhances mitochondrial-ROS and triggers mitophagy via PINK1/PARKIN, whereas ablation of β-catenin preserves mitochondrial fitness, thereby impeding parasite growth. Enhanced ROS can oxidize host mitochondrial DNA, which then functions as a host-associated molecular pattern (HAMP). This activates the cytosolic pathogen recognition receptor (PRR) AIM2, triggering the AIM2-NLRP3-ASC-caspase-1-IL-1β inflammasome cascade. This cascade leads to gasdermin-D-mediated pyroptosis, a process that critically depends on the phosphorylation of β-catenin. *T. gondii*’s ASP5 protease plays an essential role in the phosphorylation of β-catenin-mediated inflammasome activation. Metabolically, β-catenin-dependent enhanced ROS stabilized HIF-1α, which stimulates the HKII-LDH-A axis, promoting the Warburg effect, histone acetylation and pro-inflammatory M1-macrophage polarization (IL-12/IL-6/IL-23/TNF-α). β-catenin ablation shifts metabolism to oxidative-phosphorylation, fostering M2-phenotype (IL-2/IL-10/TGF-β) that abrogates parasites survival. β-catenin also strengthens MHC-TCR avidity, driving Th1/Tc1, Th9/Tc9, and Th17/Tc17 paradigm, whereas β-catenin inhibition promotes anti-inflammatory Th2/Tc2/Threg/Tcreg differentiation. Additionally, macrophage intrinsic β-catenin dictates metabolic divergence in both CD4⁺ and CD8⁺T-cells. Notably, β-catenin-deletion in macrophages protects mice (β-cat^ΔMΦ^) against infection, highlighting that XAV939 has therapeutic potential against toxoplasmosis.

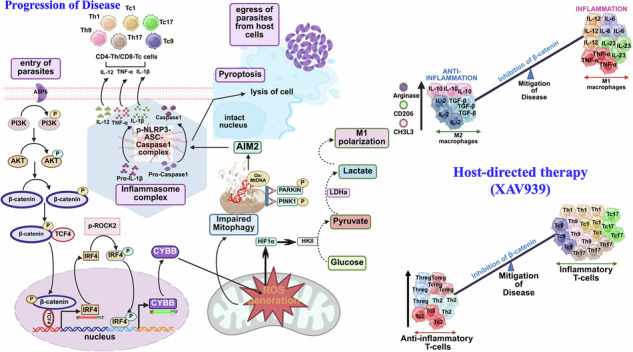

## Introduction

*Toxoplasma gondii* is an obligate intracellular protozoan parasite capable of infecting nearly all warm-blooded animals, including humans, and is among the most widespread parasitic pathogens worldwide [1]. Transmission occurs primarily through ingestion of tissue cysts in undercooked meat or oocysts from contaminated food or water. Following oral acquisition, *T. gondii* invades intestinal epithelial cells, disseminates systemically, and establishes infection in various organs by hijacking immune cells such as macrophages and dendritic cells [2, 3].

The infection can manifest as acute or chronic disease [4, 5]. Acute infection is characterized by rapid tachyzoite replication and dissemination, particularly severe in immunocompromised hosts or during primary exposure [6]. In murine models, oral infection with tissue cysts mimics natural transmission, triggering a robust and coordinated immune response involving innate and adaptive arms [7]. Pattern recognition receptors, particularly Toll-like receptors (TLRs), are key to early parasite sensing. In mice, TLR11 and TLR12 detect *T. gondii* profilin and activate MyD88-dependent IL-12 production, which drives IFN-γ-mediated immunity [8, 9]. However, humans lack functional TLR11/12, raising important questions about alternate sensing mechanisms and immune control in human macrophages [10].

To evade immune clearance, *T. gondii* actively manipulates host signaling pathways. A critical target is the PI3K-AKT axis in macrophages, which manoeuvres inflammatory responses and enhances parasite survival [11, 12]. β-catenin, a multifunctional transcriptional co-activator in Wnt signaling, is increasingly recognized for its immunomodulatory roles in macrophages. It regulates inflammatory gene expression, cytokine production, and metabolic programming, key determinants of macrophage polarization and T-cell interactions [13, 14].

During acute *T. gondii* infection, macrophages serve as first responders and central regulators of host defense. They sense the parasite through TLRs and other PRRs and secrete cytokines such as IL-12, TNF-α, and IL-1β to activate effector responses [15, 16]. However, the precise intracellular signaling cascades that balance macrophage activation(17), polarization, and inflammatory output remain incompletely defined. Studies suggest that *T. gondii* may activate pro-inflammatory cytokines and promote an M1-like, an inflammatory phenotype, potentially favoring parasite persistence [17]. While some reports indicate that *T. gondii* activates β-catenin [18] via secreted effector proteins such as GRA18 [19], others suggest suppression of canonical Wnt/β-catenin signaling [20], highlighting unresolved discrepancies.

Interferon regulatory factors (IRFs), particularly IRF4, also govern macrophage polarization and cytokine production. IRF4 expression is linked to macrophage polarization [21] and T-helper 9-cell differentiation [22]. β-catenin has been implicated in transcriptional regulation of IRFs [18], yet the β-catenin-IRF4 interaction and its relevance during *T. gondii* infection remain poorly understood.

Inflammasome activation is a hallmark of early immune responses during acute toxoplasmosis. Notably, the NLRP3 inflammasome, in coordination with absent in melanoma 2 (AIM2), mediates caspase-1 activation and the release of IL-1β and IL-18-cytokines central to inflammation and immunopathology [23, 24]. These inflammasomes are activated by mitochondrial dysfunction and reactive oxygen species (ROS), primarily produced by NADPH oxidase (CYBB/NOX2) in infected macrophages [25, 26]. Recent studies suggest β-catenin regulates mitochondrial integrity [27, 28] and ROS dynamics, potentially linking this pathway to inflammasome activation.

In addition to inflammatory signaling, metabolic reprogramming underlies macrophage functional states. Upon activation, macrophages switch from oxidative phosphorylation to aerobic glycolysis (the Warburg effect), favoring M1 polarization and pro-inflammatory output [29–31]. Wnt/β-catenin signaling is known to support this metabolic shift by stabilizing HIF1α and enhancing expression of glycolytic enzymes such as HKII and LDHA [31]. This metabolic phenotype also influences T-cell fate decisions, promoting differentiation into Th1, Th17, and other inflammatory subsets [32] essential for pathogen clearance.

Here, we investigate how *T. gondii* exploits β-catenin signaling in macrophages to orchestrate immune evasion through metabolic and inflammatory modulation. We demonstrate that infection activates the PI3K/AKT/β-catenin/IRF4/CYBB axis, leading to elevated ROS, which drives mitophagy, mitochondrial DNA oxidation, and AIM2/NLRP3 inflammasome activation. This cascade results in heightened IL-1β production and M1 polarization, further supporting inflammatory T-cell responses (Th1, Th9, Th17). Inhibition of β-catenin-genetically or pharmacologically, reduces parasite burden and skews macrophages toward an M2 phenotype with enhanced oxidative phosphorylation, thereby favoring anti-inflammatory T-cell subsets (Th2, Tc2, Tregs). Additionally, macrophage intrinsic β-catenin dictates metabolic divergence in T-cells, rewires CD4⁺T-cells toward glycolysis and disrupts TCA cycle, while CD8⁺T-cells utilize both glycolysis and mitochondrial respiration for sustained inflammation. Our findings resolve conflicting reports on β-catenin’s role and establish it as a critical node in the host-parasite interface during acute toxoplasmosis.

## Results

### Mechanisms of PI3K-AKT-β-catenin pathway activation by *T. gondii*

*T. gondii*, an intracellular protozoan parasite, can manoeuvre host cell signaling pathways to facilitate its survival and replication [33, 34]. Building on evidence that *T. gondii* activates PI3K-AKT signaling [11], we investigated whether this axis coordinates β-catenin-driven immune regulation and metabolic reprogramming, thereby influencing infection outcome. We further examined whether targeting this pathway could suppress parasite growth.

**Fig. 1:**
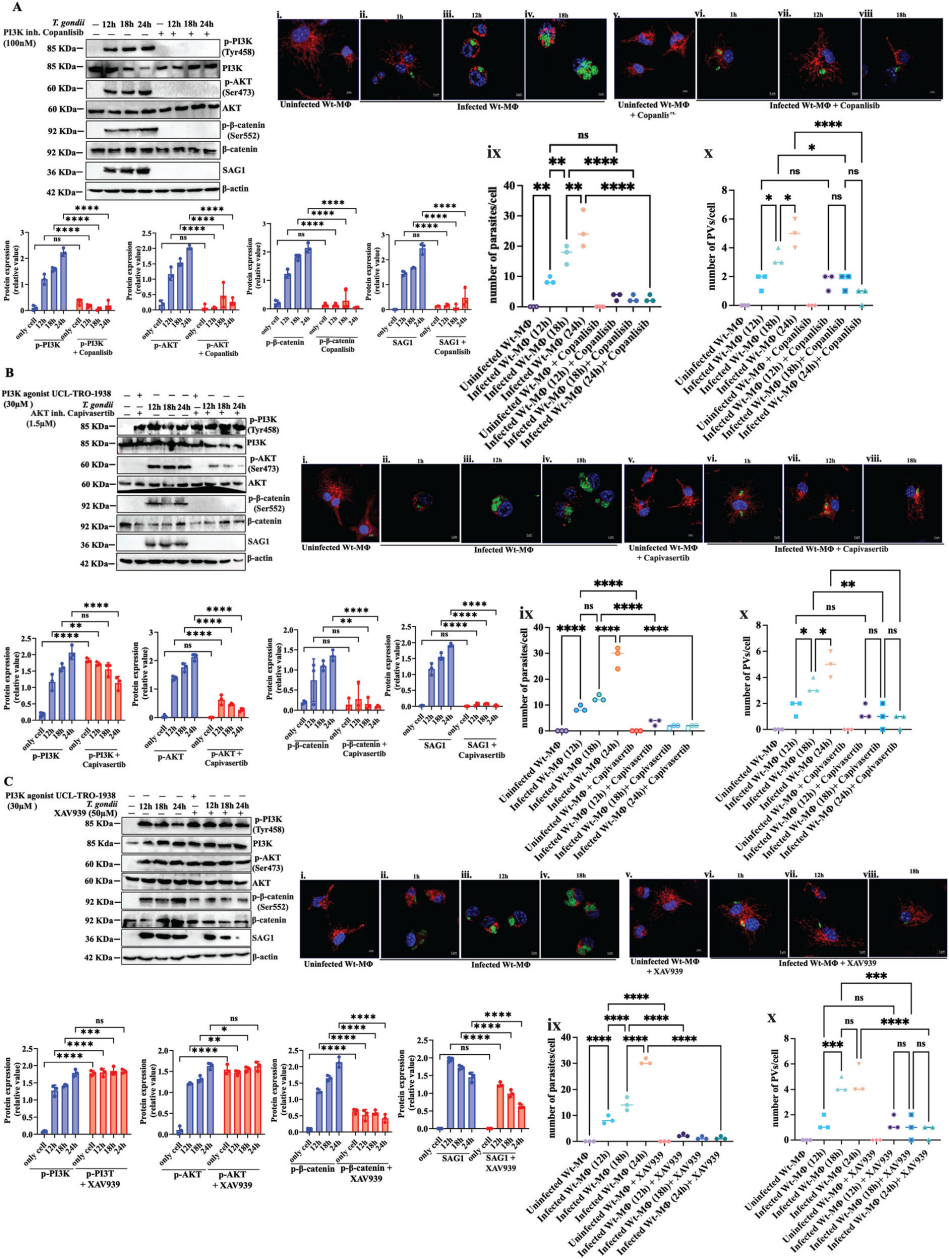
Activation of PI3K-AKT-β-catenin pathway by *T. gondii.* **A** CD11b^+^F4/80^+^ bone marrow macrophages (Wt-MΦ) derived from wild-type mice were infected with the RH strain of *T. gondii* in the presence or absence of the PI3K inhibitor copanlisib. Phospho-PI3K, phospho-AKT, phospho-β-catenin and *T. gondii* SAG1 protein at the indicated time post-infection were analysed with the total samples by immunoblotting. The average intensity of each band was plotted in bar diagram of three individual experiments after normalizing against total β-actin. Data shown are the mean ± SEM (*n* = 3) with statistical significance assessed using two-way ANOVA. Statistical significance is indicated as ****p < 0.0001, and “ns” representing no significance. (i-viii) Wt**-**MΦ infected with the GFP-RH strain of *T. gondii* in the presence or absence of the copanlisib and at the indicated times, stained for mitochondria (Mito tracker, red) and nucleus (DAPI, blue) and visualized by confocal microscopy. Scale bars represent 5 μm. (ix) The number of parasites within infected cells and (x) the total number of PVs per cell were quantified and presented as bar graphs. Data are expressed as mean ± SEM (*n* = 3), and statistical significance was determined using one-way ANOVA. Significance levels are indicated as: ****p < 0.0001, **p < 0.01, *p < 0.05*;* “ns” representing no significance. **B** Wt-MΦ infected with the RH strain of *T. gondii* were analyzed in the presence or absence of the AKT inhibitor capivasertib. PI3K agonist UCL-TRO-1938 was used to activate PI3K. Phospho-PI3K (85Kda), phospho-AKT (60Kda), phospho-β-catenin and SAG1 protein at the indicated time post-infection were analysed with the total samples by immunoblotting. The average intensity of each band was plotted in bar diagram of three individual experiments after normalizing against total β-actin. Data shown are the mean ± SEM (*n* = 3) with statistical significance assessed using two-way ANOVA. Statistical significance is indicated as ****p < 0.0001, **p < 0.01, and “ns” representing no significance. (i-viii) Wt-MΦ infected with the GFP-RH strain of *T. gondii* in the presence or absence of the capivasertib and at the indicated times, stained for mitochondria (Mito tracker, red) and nucleus (DAPI, blue) and visualized by confocal microscopy. Scale bars represent 5 μm. (ix) The number of parasites within infected cells and (x) the total number of PVs per cell were quantified and presented as bar graphs. Data are expressed as mean ± SEM (*n* = 3), and statistical significance was determined using one-way ANOVA. Significance levels are indicated as: ****p < 0.0001, **p < 0.01, *p < 0.05*;* “ns” representing no significance. **C** Wt-MΦ infected with the RH strain of *T. gondii* were analyzed in the presence or absence of the β-catenin inhibitor XAV939. PI3K agonist UCL-TRO-1938 was used to activate the PI3K-AKT signaling axis. Phospho-PI3K, phospho-AKT, phospho-β-catenin and SAG1 protein at the indicated time post-infection were analysed with the total samples by immunoblotting. The average intensity of each band was plotted in bar diagram of three individual experiments after normalizing against total β-actin. Data shown are the mean ± SEM (*n* = 3) with statistical significance assessed using two-way ANOVA. Statistical significance is indicated as ****p < 0.0001, ***p < 0.001, **p < 0.01, *p < 0.05, and “ns” representing no significance. (i-viii) Wt-MΦ infected with the GFP-RH strain of *T. gondii* in the presence or absence of the XAV939 and at the indicated times, stained for mitochondria (Mito tracker, red) and nucleus (DAPI, blue) and visualized by confocal microscopy. Scale bars represent 5 μm. (ix) The number of parasites within infected cells and (x) the total number of PVs per cell were quantified and presented as bar graphs. Data are expressed as mean ± SEM (*n* = 3), and statistical significance was determined using one-way ANOVA. Significance levels are indicated as: ****p < 0.0001, ***p < 0.001, *p < 0.05*;* “ns” representing no significance.

We observed phosphorylation of PI3K-Tyr458, AKT-Ser473, and β-catenin-Ser552 during infection in Wt-MΦ (Wt-BMDM) (Fig. 1A, lanes 2-4). Treatment with the PI3K inhibitor copanlisib abolished PI3K phosphorylation and consequently prevented phosphorylation of AKT and β-catenin, resulting in impaired parasite growth (lanes 6-8, Fig. 1A). Microscopy confirmed higher parasite loads and distinct parasitophorous vacuoles (PVs) in untreated infected Wt-MΦ compared to copanlisib-treated cells (Fig. 1Aii–viii). Quantification showed significant reductions in parasite number and PVs upon copanlisib treatment (Fig. 1Aix–x). The effect was dose-dependent, with 100 nM copanlisib fully inhibiting PI3K phosphorylation without cytotoxicity (Figs. S1Ai and Aiv). siRNA-mediated PI3K silencing yielded similar results, reducing PI3K phosphorylation, downstream AKT and β-catenin activation, and parasite growth (Fig. S1B).

Using the AKT inhibitor capivasertib, we observed inhibition of AKT and β-catenin phosphorylation without affecting PI3K phosphorylation (lanes 7-9, Fig. 1B), confirming that PI3K acts upstream. Capivasertib-treated cells showed reduced parasite burden and PV formation compared to untreated infected Wt-MΦ (Fig.1Bii–viii), with significant reductions in parasite number and PVs (Fig. 1Bix–x). Complete inhibition of AKT phosphorylation was achieved at 1.5 μM without cytotoxicity (Fig. S1Aii, Aiv). siRNA-mediated AKT knockdown similarly reduced AKT expression, blocked β-catenin phosphorylation, and impaired parasite growth (Fig. S1C).

To directly assess β-catenin, we used XAV939, a tankyrase 1/2 inhibitor. XAV939 abrogated β-catenin activation and inhibited parasite growth (lanes 6-8, seventh panel, Fig. 1C). In cells treated with both a PI3K agonist and XAV939, phosphorylation of PI3K (lane 5, first panel, Fig. 1C) and AKT (lane 5, third panel, Fig. 1C) persisted, whereas β-catenin phosphorylation at Ser552 was blocked (lane 5, fifth panel, Fig. 1C), confirming that β-catenin functions downstream of PI3K-AKT. Untreated infected Wt-MΦ exhibited higher parasite loads and distinct PVs (Fig. 1Cii–iv) compared to XAV939-treated cells (Fig. 1Cvi–viii), with significant reductions in parasite number and PVs upon treatment (Fig. 1Cix–x). The inhibitory effect was dose-dependent, with 50 μM XAV939 completely abrogating β-catenin expression without cytotoxicity (Fig. S1Aiii, Aiv, J). Collectively, these findings demonstrate that *T. gondii* exploits the PI3K-AKT-β-catenin axis through phosphorylation of key components (Fig. 1A–C). Disruption of this cascade at the level of PI3K (copanlisib), AKT (capivasertib), or β-catenin (XAV939) consistently blocks downstream signaling, reduces parasite proliferation, and limits PV formation, highlighting this pathway as a promising target for host-directed therapeutic intervention.

### Preferential binding of inhibitors toward mammalian PI3K, AKT, and TNKS1 supports a host-directed therapeutic strategy

To evaluate whether the inhibitors used in this study preferentially interact with host proteins over their *T. gondii* homologs, we performed comparative molecular docking analyses using structural models of PI3K, AKT, and TNKS1 from both mammalian and parasitic systems (Fig. S2). Overall, the analysis revealed consistent differences in binding pocket architecture and docking scores between host and parasite proteins, suggesting differential ligand compatibility while supporting a host-directed therapeutic approach.

Briefly, with the PI3K enzyme, docking of copanlisib within the mammalian enzyme revealed a well-defined and enclosed binding pocket that supports extensive ligand stabilization (Fig. S2i.A, C). The docking results indicate that the mammalian PI3K inhibitor is deeply anchored within the catalytic pocket and stabilized by a dense hydrogen-bonding network with Lys833, Asp841, Asp884, and Asp964, predominantly through interactions involving the ligand’s pyrimidine ring [35]. In contrast, the *T. gondii* PI3K homolog exhibited a comparatively more open and solvent-accessible binding pocket (Fig. S2i.B, D), resulting in a marked reduction in the interaction profile. Here, copanlisib primarily engaged residues such as Cys1386, Lys833, and Val1382, forming fewer stabilizing contacts. Notably, the absence of key acidic residues, including an aspartate present in the mammalian active site, likely limits electrostatic stabilization of the ligand, with the pyrimidine ring only forming interactions with the Lys833 residue. These structural differences were reflected in comparatively lower docking scores for the parasitic homolog, indicating less favourable binding of the inhibitor to the *T. gondii* PI3K.

A similar trend of differential affinity was also observed for capivasertib, which in the mammalian system binds to AKT kinase. In the mammalian AKT structure, capivasertib was accommodated within a compact and well-defined binding pocket (Fig. S2ii.A, C), forming multiple stabilizing interactions with residues K158, Y229, A230, E234, E278, M281, and D292. These interactions support a stable binding conformation and reflect compatibility with the conserved kinase domain. In contrast, the *T. gondii* AKT homolog, the kinase, exhibits a less compact binding pocket (Fig. S2ii.B, D) [36]. This resulted in fewer interactions, while in the mammalian system, the inhibitor also interacts with A230 and D292; these interactions were absent in the *T. gondii* AKT due to the differential pocket architecture. Thus, the overall interaction network was less extensive, and the docking score (-8.34) suggested only moderate affinity, likely due to differences in active-site geometry and residue composition relative to the mammalian protein.

For TNKS1, docking of XAV939 further highlighted pronounced differences between host and parasite homologs [37]. In the mammalian TNKS1 protein, the ligand was well accommodated within a defined and enclosed binding pocket (Fig. S2iii.A, C), forming multiple stabilizing interactions, including hydrogen bonds with Tyr1206, Gly1204, His1177, Pro1176, and His1269. These interactions are consistent with the conserved catalytic domain of mammalian TNKS and support stable ligand binding. In contrast, the *T. gondii* TNKS homolog exhibited a markedly altered structural architecture (Fig. S2iii.B, D), in which the ligand failed to occupy a defined active site and instead appeared largely surface-exposed. Only very limited interactions were observed, and the docking score (-1.42) indicated very weak binding affinity. Furthermore, blind docking suggested that the ligand interacts with non-conserved regions rather than a canonical catalytic pocket, reinforcing the lack of inhibitor specificity.

Taken together, these results demonstrate that while the inhibitors form stable and well-defined interactions with mammalian proteins, their interactions with *T. gondii* homologs are comparatively weaker and associated with lower docking scores. These findings suggest a preferential binding of these inhibitors toward mammalian targets, consistent with a host-directed therapeutic mechanism.

Since molecular docking only provides a predictive, static view of ligand-protein interactions, to further verify that copanlisib (PI3K inhibitor), capivasertib (AKT inhibitor), and XAV939 (TNKS1 inhibitor) indeed are specific and do not affect tachyzoite viability, we pre-incubated extracellular RH tachyzoites with each inhibitor at the same concentrations used for cellular protein inhibition for 30 min. Following incubation, these treated parasites were used to infect Wt-MΦ for 12 h. Western blot analysis of parasite growth revealed no differences between vehicle-exposed parasites and those pre-exposed to any of the three inhibitors, indicating that none of the compounds impair tachyzoite viability or replication (Fig. S1N).

Taken together, the docking analyses, along with siRNA-mediated knockdown of PI3K, AKT, and β-catenin, demonstrate that these inhibitors act selectively on host enzymes rather than on their *T. gondii* counterparts. This host-directed mode of action mitigates parasite infection while minimizing direct selective pressure on the parasite, highlighting the therapeutic promise of targeting host signaling pathways.

### Phosphorylation of β-catenin is essential for *T. gondii* replication

**Fig. 2:**
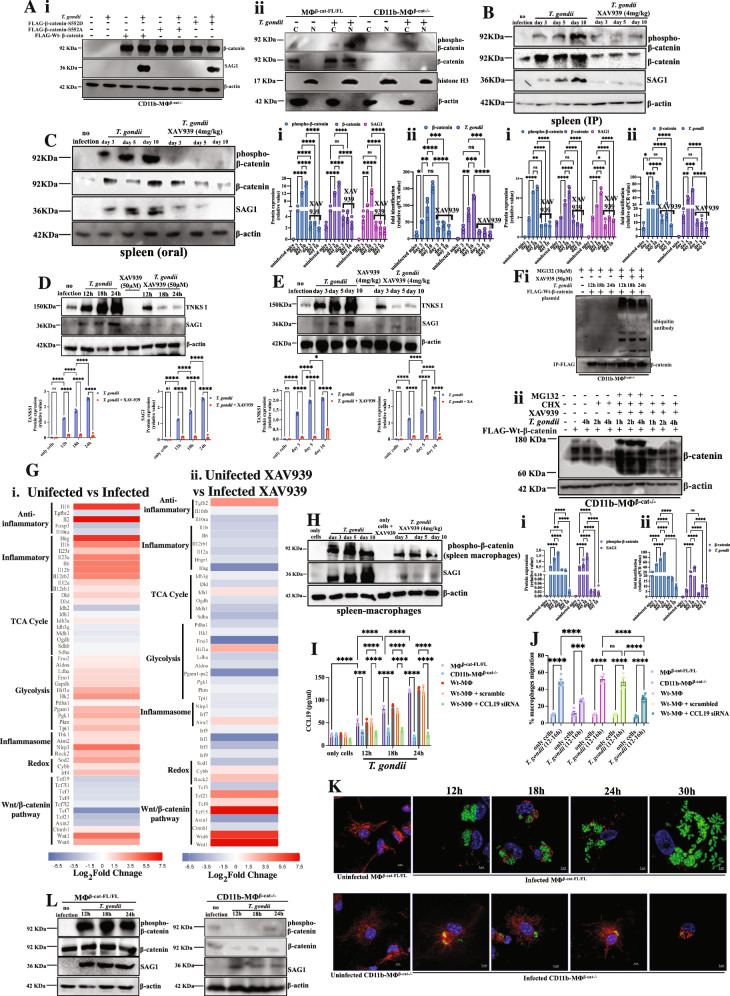
Pharmacological or genetic inhibition of β-catenin abrogates replication of parasites. **A** i CD11b-MΦ^β-cat⁻/⁻^ macrophages from β-cat^ΔMΦ^ mice were transfected with FLAG-tagged wild-type (Wt), phospho-mutant (S552A), or phospho-mimic (S552D) β-catenin plasmids, either in the presence or absence of *T. gondii* infection for 12 h. The samples were then subjected to immunoblot analysis using antibodies against β-catenin, and SAG1 to assess their expression levels. β-actin antibody was used as an internal loading control. ii. To determine the localization of phospho-β-catenin, nuclear (N) and cytoplasmic (C) fractions were isolated from MΦ^β-cat-FL/FL^ and CD11b-MΦ^β-cat⁻/⁻^ cells at 12 h.p.i. Immunoblotting was performed using antibodies against phospho-β-catenin and total β-catenin, with fraction purity confirmed by histone H3 as a nuclear marker and β-actin as a cytoplasmic marker. Spleens of **B** i.p. infected mice, **C** oral infected mice were collected, and total lysates were subjected to immunoblotting to detect phospho-β-catenin, total β-catenin, SAG1, and β-actin (loading control). **B, C** i. The average band intensities from three independent experiments were normalized to total β-actin and plotted as bar graphs for phospho-β-catenin, total β-catenin, and SAG1. Data shown are the mean ± SEM (*n* = 3) with statistical significance assessed using two-way ANOVA. Statistical significance is indicated as ****p < 0.0001, ***p < 0.001, **p < 0.01, *p < 0.05, and “ns” for no significance. **B, C** ii. The growth of *T. gondii* (*B1*) and expression of β-catenin (*CTNNB1*) in spleen-i.p. infected mice (**B**), spleen-oral infected mice (**C**) treated with XAV939 were quantified by qPCR. Data shown are the mean ± SEM (*n* = 3) with statistical significance assessed using two-way ANOVA. Statistical significance is indicated as ****p < 0.0001, ***p < 0.001, **p < 0.01, *p < 0.05, and “ns” indicating no significance. **D** Wt-MΦ were infected ex vivo with the RH strain in the presence or absence of the tankyrase 1 inhibitor (XAV939). The expression levels of tankyrase 1 (TNKS1) and SAG1 were analyzed at the indicated time points post-infection using immunoblotting of total cell lysates. The average band intensities for TNKS1 and SAG1 from three independent experiments were normalized to β-actin and presented as bar graphs. Data shown are the mean ± SEM (*n* = 3) with statistical significance assessed using two-way ANOVA. Statistical significance is indicated as ****p < 0.0001. **E** In Wt mice i.p. infected with the RH strain, one group was treated with XAV939, while the other group remained untreated. At the indicated days post-infection, spleens were collected, and the expression levels of TNKS1 and SAG1 were analyzed via immunoblotting of total splenocytes. Average band intensities for TNKS1 and SAG1 were normalized to β-actin and plotted as bar graphs based on data from three independent experiments. Data shown are the mean ± SEM (*n* = 3) with statistical significance assessed using two-way ANOVA. Statistical significance is indicated as ****p < 0.0001, *p < 0.05 and “ns” indicating no significance. **F** i CD11b-MΦ^β-cat⁻/⁻^ macrophages were transfected with FLAG-tagged Wt-β-catenin plasmid in the presence of MG132 and subsequently infected with RH strain. One set was treated with XAV939, while the other remained untreated. Total cell lysates were subjected to immunoprecipitation using a FLAG antibody, followed by immunoblotting with a ubiquitin antibody. (ii) CD11b-MΦ^β-cat⁻/⁻^ macrophages (lane 1) confirm antibody specificity. Reconstitution with FLAG-Wt-β-catenin restores protein expression (lane 2), which remains detectable upon infection (lane 3). Treatment with CHX (lanes 4-5) results in a time-dependent decrease in β-catenin levels, indicating protein turnover. Co-treatment with XAV939 (lanes 9-11) accelerates β-catenin degradation, whereas addition of the proteasome inhibitor MG132 in the presence of XAV939 (lanes 6-8) partially rescues β-catenin levels and leads to the accumulation of higher molecular weight β-catenin species, consistent with ubiquitinated forms. **G** KEGG analysis was performed on differentially expressed genes (>2 fold, *p*-value < 0.05) between i. splenocytes of Uninfected vs Infected mice and ii. splenocytes of Uninfected XAV939 vs Infected XAV939 treated mice. Heatmaps were generated with genes selected from representative KEGG pathways. (Red: high, blue: low expression). Both up- and down-regulated genes of the host cell were analyzed together. **H** CD11b⁺F4/80⁺ macrophages were isolated from the spleen of uninfected and infected mice using FACS sorting with CD11b and F4/80 antibodies. Phospho-β-catenin and SAG1 protein levels were analyzed by immunoblotting at the indicated time points post-infection. **(i)** The average intensity of each band was plotted in bar diagram of three individual experiments after normalizing against total β-actin. Data shown are the mean ± SEM (*n* = 6) with statistical significance assessed using two-way ANOVA. Statistical significance is indicated as, ****p < 0.0001, ***p < 0.001, **p < 0.01, *p < 0.05, and “ns” indicating no significance. (ii) The growth of *T. gondii* (*B1*) and expression of β-catenin (*CTNNB1*) in macrophages of spleen from infected mice treated with XAV939 were quantified by qPCR. Data shown are the mean ± SEM (*n* = 6) with statistical significance assessed using two-way ANOVA. Statistical significance is indicated as ****p < 0.0001, ***p < 0.001, **p < 0.01, *p < 0.05 and “ns” indicating no significance. **I** CCL19 chemokine levels in culture supernatants were quantitatively measured by ELISA from in MΦ^β-cat-FL/FL^, CD11b-MΦ^β-cat⁻/⁻^, Wt-MΦ macrophages and Wt-MΦ macrophages transfected with either scrambled siRNA or CCL19-specific siRNA following ex vivo infection with RH *T. gondii* parasites. Data shown are the mean ± SEM (*n* = 4) with statistical significance assessed using two-way ANOVA. Statistical significance is indicated as ****p < 0.0001. **J** The transmigration ability of MΦ^β-cat-FL/FL^, CD11b-MΦ^β-cat⁻/⁻^, Wt-MΦ macrophages and Wt-MΦ macrophages transfected with either scrambled siRNA or CCL19-specific siRNA following infection was evaluated using a transwell assay. The graph presents representative data of the mean ± SEM (*n* = 4) with statistical significance assessed using two-way ANOVA. Statistical significance is indicated as ****p < 0.0001. **K** MΦ^β-cat-FL/FL^ and CD11b-MΦ^β-cat⁻/⁻^ cells were infected with the GFP-RH strain of *T. gondii* and, at the indicated time points, stained for mitochondria (MitoTracker, red) and nuclei (DAPI, blue). The cells were visualized using confocal microscopy, with scale bars representing 5 μm. **L** MΦ^β-cat-FL/FL^ and CD11b-MΦ^β-cat⁻/⁻^ cells were infected with *T. gondii*, and at the specified time points, the cells were harvested for immunoblot analysis using antibodies against phospho-β-catenin, β-catenin, SAG1, and β-actin (loading control).

To determine whether β-catenin phosphorylation at S552 is required for parasite growth, CD11b-MΦ^β-cat⁻/⁻^ macrophages were purified using CD11b and F4/80 markers. These cells failed to support *T. gondii* proliferation (lane 2, second panel, Fig. 2Ai). Transfection with FLAG-Wt-β-catenin restored β-catenin expression (lane 3, first panel, Fig. 2Ai) and, after infection, overexpressed β-catenin (lane 4, first panel, Fig. 2Ai) supports parasites growth (lane 4, second panel, Fig. 2Ai). In contrast, FLAG-β-catenin-S552A-transfected CD11b-MΦ^β-cat⁻/⁻^ macrophages showed expression of β-catenin (lane 6, first panel, Fig. 2Ai) but abrogated parasite growth (lane 6, first and third panels) as the phosphorylation domain of β-catenin mutated. Transient expression of the phospho-mimetic FLAG-β-catenin-S552D in CD11b-MΦ^β-cat⁻/⁻^ macrophages induced further increase of parasite infection, supporting parasite growth (lane 8, first and second panels, Fig. 2Ai).

Immunoblot analysis of cytoplasmic and nuclear fractions revealed that phosphorylated β-catenin translocated into the nucleus upon infection in MΦβ-cat^FL/FL^ macrophages (Fig. 2Aii), whereas no nuclear β-catenin was detected in CD11b-MΦ^β-cat⁻/⁻^ cells. These results indicate that phosphorylation of β-catenin at S552 is indispensable for *T. gondii* growth in macrophages, with nuclear translocation of phospho-β-catenin directly correlating with increased parasite proliferation as detected by SAG1 expression.

### Abrogation of β-catenin halts parasite growth in mice

All core experiments were conducted using intraperitoneal (i.p.) infection with *T. gondii* RH strain tachyzoites to ensure synchronized systemic dissemination and reproducible assessment of immune responses. This route provides precise dose control and is widely accepted as a reliable model of acute toxoplasmosis [5, 38, 39]. To demonstrate that β-catenin mediated effects are not organ or route-specific, we included supporting experiments using oral cysts infection of ME49 [40], which mimics natural transmission. Together, these complementary infection models demonstrate that the β-catenin mediated effects are not route-dependent, thereby reinforcing the broader relevance of our findings across distinct anatomical and immunological environments.

First, we aim to investigate how inhibition of β-catenin signaling influences survival, weight maintenance, immune function, and metabolic regulation during *T. gondii* infection. Hence, we utilized two distinct infection models: (i) i.p. injection of 50 tachyzoites of the RH strain [41–43]. and (ii) oral administration of 100 cysts. We assessed the systemic dissemination of parasites in the spleen in both models. This study specifically focuses on the acute phase of infection rather than chronic infection. To inhibit β-catenin signaling in in vivo system, we employed two complementary strategies: (i) genetic deletion of β-catenin specifically in macrophages using β-cat^ΔMΦ^ mice, with β-catenin^flox^ mice as wild-type controls; and (ii) pharmacological inhibition using Wnt/β-catenin pathway inhibitors like XAV939, JW55, and Wnt-C59 to identify the most effective compounds for enhancing resistance to infection and reducing infection-associated weight loss. All inhibitors were delivered via the i.p. to 1 day post infected mice for seven consecutive days at a dose of 4 mg/kg body weight per day, based on their determined LC_50_ values.

Among the tested compounds, XAV939 and Wnt-C59 demonstrated the highest efficacy, each achieving over 80% survival in i.p. infected mice (Fig. S1D) without significant weight loss (Fig. S1E). Notably, both i.p. (Fig. S1G) and orally (Fig. S1I) infected wild-type and β-catenin^flox^ mice experienced over 30% body weight loss by 10 days post-infection, with more than 50% mortality in both i.p. (Fig. S1D, F) and the orally infected groups (Fig. S1H).

In contrast, mice treated with XAV939 or Wnt-C59 whether infected i.p. (Fig. S1D) or orally (Fig. S1H) as well as β-cat^ΔMΦ^ mice infected i.p. (Fig. S1F) or orally (Fig. S1H), exhibited markedly enhanced resistance to infection, with survival rates exceeding 80% compared to infected wild-type and β-catenin^flox^ controls (Fig. S1D, F, H). These results indicate that inhibition of β-catenin signaling, either via genetic ablation (in β-cat^ΔMΦ^ mice) or pharmacological intervention, significantly impairs parasite proliferation during acute infection regardless of the route of infection thereby preventing excessive weight loss (Fig. S1E, G, I) and improving overall survival outcomes (Fig. S1D, F, H).

Based on its superior efficacy and lower cytotoxicity compared to Wnt-C59 (Fig. S1K), we selected XAV939 (Fig. S1Aiv,J) for use in subsequent experiments to inhibit β-catenin signaling.

To investigate the role of β-catenin in systemic infection and parasite dissemination in mice, we collected spleen tissue following i.p. infection with RH (Fig. 2B) and oral infection with cysts (Fig. 2C) at various time points post-infection. The levels of phospho-β-catenin and *T. gondii* were assessed using both western blotting (Fig. 2B, Ci) and qPCR (Fig. 2B, Cii). We found a concomitant augmentation in phosphorylation of β-catenin-S552 in spleen (lane 2-4, first panel, Fig. 2B, C), and *T. gondii* growth during both i.p. and oral infection (lane 2-4, third panel, Fig. 2B, C), but in XAV939-treated infected mice, both levels of β-catenin (lane 5-7, second panel, Fig. 2B, C) and phosphorylated β-catenin (lane 5-7, first panel, Fig. 2B, C) were curtailed down. Attenuation of β-catenin signaling by XAV939 prevented parasite growth in spleen (lane 5-7, third panel, Fig. 2B, C), considerably improving mice weight (Fig. S1E, G, I) and survivability (Fig. S1D, F, H). This is because, treatment with XAV939 impaired β-catenin transcription and translation, as evidenced by decreased β-catenin at protein (Fig. 2Bi, Ci) and mRNA levels (Fig. 2Bii, Cii).

To test the efficiency of XAV939, we looked for expression of tankyrase1 through ex vivo (Fig. 2D) and in vivo (i.p.) (Fig. 2E) infection and we found an increase in tankyrase1 over time following infection in both bone marrow-derived macrophages (Wt-MΦ) (lanes 2-4, first panel, Fig. 2D) and in the spleens of infected mice (lanes 2-4, first panel, Fig. 2E). This increase in tankyrase1 was associated with enhanced parasite growth in infected Wt-MΦ (lanes 2-4, second panel, Fig. 2D) and in the spleens of infected mice over the course of infection (lanes 2-4, second panel, Fig. 2E). Treatment with XAV939 in Wt-MΦ inhibited tankyrase1 expression (lane 5, first panel, Fig. 2D), and this inhibition of tankyrase1 by XAV939 reduced parasite growth (lanes 6-8, second panel, Fig. 2D). Similarly, in vivo treatment of infected mice with XAV939 inhibited tankyrase1 expression at different days post-infection (lanes 6-8, first panel, Fig. 2E), which consequently reduced parasite growth in the spleens of infected mice (lanes 6-8, second panel, Fig. 2E).

Thus, we elucidated the mechanism of action of XAV939 on β-catenin and found that XAV939 facilitates β-catenin degradation via the ubiquitin-mediated proteasomal pathway (Fig. 2Fi,ii). For this CD11b-MΦ^β-cat–/–^ cells were transfected with FLAG-Wt-β-catenin and treated with MG132. MG132 is a proteasome inhibitor that prevents the degradation of proteins targeted for ubiquitin-mediated proteolysis. MG132 treatment stabilizes and promotes the accumulation of ubiquitinated β-catenin, including phosphorylated forms, thereby enabling their detection and facilitating the study of their functional roles. Cells were then infected, with or without XAV939, and lysates were collected at various time points post-infection. Immunoprecipitation using FLAG antibody followed by immunoblotting with anti-ubiquitin antibody revealed that Wt-β-catenin was ubiquitinated during infection conditions only in the presence of XAV939 (Fig. 2Fi). These findings confirm that β-catenin is a substrate of the ubiquitin-proteasome system (UPS), and XAV939 causes β-catenin degradation during infection by inhibiting tankyrase 1 (Fig. 2Fi). To examine whether XAV939 regulates β-catenin stability through proteasome-associated degradation, we performed a cycloheximide (CHX) chase assay in β-catenin-reconstituted macrophages under *T. gondii* infection, in the presence or absence of XAV939 and the proteasome inhibitor MG132 (Fig. 2Fii). β-catenin was undetectable in knockout macrophages (lane 1), confirming antibody specificity. Reconstitution with FLAG-Wt-β-catenin (lane 2) restored protein expression under basal conditions. Upon *T. gondii* infection, β-catenin levels remained detectable at 4 h.p.i. (lane 3), indicating that infection alone does not markedly reduce β-catenin abundance.

Inhibition of protein synthesis with CHX (lanes 4-5) resulted in a time-dependent decrease in β-catenin levels, reflecting ongoing protein turnover. Notably, treatment with XAV939 (lanes 9-11) markedly accelerated this reduction, demonstrating decreased β-catenin stability. In contrast, co-treatment with MG132 in the presence of XAV939 (lanes 6-8) partially attenuated β-catenin degradation and led to the accumulation of higher molecular weight β-catenin species, consistent with ubiquitinated forms. Together with the increased ubiquitination observed (Fig. 2Fi), these findings indicate that XAV939 promotes β-catenin degradation by reducing its stability and enhancing its ubiquitin-dependent turnover, in a manner consistent with proteasome-mediated degradation.

### β-catenin signaling promotes *T. gondii*-induced splenic immune activation

To assess whether β-catenin inhibition alleviates infection-induced pathology, we monitored mouse survival (Fig. S1D, F, H) and body weight changes (Fig. S1E, G, I). To examine the role of β-catenin in infection-induced pathology, we quantified spleen index following i.p. infection with tachyzoites (Fig. S1M). In Wt and β-catenin^flox^ mice, infection induced pronounced splenomegaly (~60.11 ± 3.10% to ~78.90 ± 1.64%, *p* < 0.001) (Fig. S1M). In contrast, infected β-cat^ΔMΦ^ mice and XAV939-treated infected mice did not develop splenomegaly with spleen indices (~66.8 ± 3.5% and ~66.2 ± 2.9%) comparable to uninfected controls (ns, *p* > 0.05). These findings demonstrate that genetic deletion or pharmacological inhibition of β-catenin prevents systemic immune activation during acute *T. gondii* infection.

### Transcriptome profiling reveals β-catenin-dependent immune and metabolic reprogramming upon acute *T. gondii* infection

To gain a comprehensive understanding of the molecular alterations induced by *T. gondii* infection, we performed whole transcriptome analysis of mouse splenocytes infected with RH strain parasites through i.p. The analysis focused on identifying significant changes in RNA expression across different experimental groups, including uninfected versus infected mice (Fig. 2Gi) and uninfected XAV939-treated versus infected XAV939-treated mice (Fig. 2Gii). Kyoto encyclopedia of genes and genomes (KEGG) pathway enrichment analysis revealed substantial modulation in several critical biological pathways. Genes involved in inflammatory and anti-inflammatory responses were markedly dysregulated upon infection, highlighting the parasite’s impact on the host immune landscape (Fig. 2Gi). Similarly, pathways related to energy metabolism, including glycolysis and the tricarboxylic acid (TCA) cycle, showed significant alterations, reflecting a parasite-induced metabolic reprogramming. Additionally, genes associated with redox signaling were differentially expressed, indicating oxidative stress and redox imbalance during infection (Fig. 2Gi). XAV939 treatment in infected mice modulated many of these pathways (Fig. 2Gii), advocating the potential role of β-catenin in infection-induced dysregulation. The transcriptomic analysis of infected mice revealed significantly higher expression of several genes associated with the PI3K-Wnt/β-catenin pathway, including *Wnt1* (Wingless-Type MMTV Integration Site Family Member 1), *Tnks*/*Tnks2* (tankyrase 1/2), *Pi3kcb* (Phosphoinositide-3-kinase catalytic subunit beta), and *Ctnnb1* (β-catenin) (Fig. S4A). Notably, administration of XAV939 (4 mg/kg body weight) significantly suppressed the expression of these genes, both in uninfected and infected mice (Fig. S4B–D). Collectively, these findings provide valuable insights into the molecular mechanisms underlying host responses to *T. gondii* infection and the therapeutic impact of XAV939, shedding light on potential targets for intervention.

### β-catenin supports parasites growth in macrophages

While previous studies have established β-catenin’s regulatory effects on macrophage polarization and inflammation, its direct interactions with other signaling pathways in infected macrophages are not well characterized. A critical gap in understanding β-catenin’s function in *T. gondii* infection lies in elucidating the precise molecular mechanisms by which β-catenin signaling influences macrophage responses, particularly inflammasome activation and ROS generation. Additionally, the therapeutic potential of targeting β-catenin in macrophages requires further investigation to understand its broader impact on immune responses, tissue repair, and parasites. For this, we purified CD11b^+^F4/80^+^ macrophages from spleen of uninfected and infected Wt mice (i.p.). Macrophages isolated from the spleen (Fig. 2H) of infected mice harbored parasites, with an observed increase in replication over time, indicating that *T. gondii* actively proliferated within these macrophages as the infection progressed. The enhanced growth of parasites in macrophages was concomitant with phosphorylation of β-catenin. While XAV939-treated infected mice exhibited suppressed phosphorylated β-catenin expression along with effectively inhibited parasite growth in macrophages from the spleen (Fig. 2H). Treatment with XAV939 impaired β-catenin transcription and translation, as evidenced by reduced phospho-β-catenin protein levels (Fig 2Hi) and decreased mRNA expression (Fig. 2Hii). To study th**e** dynamic interactions between *T. gondii* and host cells, live-cell imaging was conducted on *T. gondii* infected MΦ^β-cat-FL/FL^ macrophages (Video S1A). The imaging revealed internalization of *T. gondii* tachyzoites into MΦ^β-cat-FL/FL^ began within 1–3 h post-infection (h.p.i.) after the tachyzoites were introduced into the macrophages. The tachyzoites remained loosely adherent to macrophage cells for approximately 20 min before actively invading the host cells. Upon entry, tachyzoites successfully invaded MΦ^β-cat-FL/FL^ cells, underwent division into daughter cells, and initiated organization for PVs formation. Fully developed PVs were then observed between 8 and 12 h.p.i. At 12 h.p.i., infected MΦ^β-cat-FL/FL^ macrophages exhibited increased migration (Fig. 2J, Video S1A, 12-16 h.p.i.), facilitating cell fusion and subsequently enhancing the parasite load (Video S1A, 12-16 h.p.i.). At 12-16 h.p.i., the trafficking of macrophages helped them to fuse with each other to increase the infection rate followed by the bursting of PVs between 24-30 h.p.i., releasing new parasites that subsequently infected neighbouring uninfected cells. In CD11b-MΦ^β-cat⁻/⁻^, *T. gondii* tachyzoites were able to enter the cells efficiently, with rates of internalization comparable to those observed in β-catenin^FL/FL^ (wild-type) macrophages during the early stages of infection (Video S1B, 1-3 h.p.i.). However, at later stages (8-16 h.p.i.), although parasites remained intracellular, they exhibited impaired mature PV formation and failed to replicate effectively (Video S1B). By 24 h.p.i., parasite viability was markedly reduced, cells then subsequently underwent shrinkage and death without forming functional PVs likely due to the absence of a permissive intracellular environment required for survival and replication suggesting that β-catenin signaling in macrophages contributes critically to establishing a host cell niche conducive to *T. gondii* proliferation (Video S1).

Earlier studies have shown that CCL19 is a key chemokine involved in immune cells trafficking and migration [44]. To investigate the role of β-catenin in the migration of infected macrophages, we quantitatively measured CCL19 chemokine secretion in culture supernatants using ELISA (Fig. 2I). Infected β-catenin^FL/FL^ macrophages and Wt-MΦ macrophages showed a time-dependent increase in CCL19 production (Fig. 2I), which correlated with enhanced migratory capacity during the course of infection (Fig. 2J). In contrast, CD11b-MΦ^β-cat⁻/⁻^ macrophages failed to secrete detectable levels of CCL19 (Fig. 2I), and their migration following infection was significantly impaired (Fig. 2J), indicating that β-catenin signaling is essential for infection-induced chemokine production and macrophage motility. To directly test the functional role of CCL19, we silenced its expression using siRNA in Wt-MΦ macrophages. Knockdown of CCL19 effectively abrogated its secretion during infection in Wt-MΦ macrophages, as confirmed by ELISA (Fig. 2I), and led to a marked reduction in macrophage migration (Fig. 2J). These findings highlight the importance of β-catenin-mediated CCL19 production in facilitating the migration of infected macrophages. Therefore, β-catenin signaling not only regulates macrophage-intrinsic responses like CCL19 secretion and motility and may contribute to parasite dissemination within the host (Video S1A).

Confocal microscopy of fixed MΦ^β-cat-FL/FL^ macrophages revealed that *T. gondii* tachyzoites initiated PVs formation by 12 h post-infection (h.p.i.), accompanied by the migration of mitochondria towards the PV periphery (Fig. 2K). By 24 h.p.i., mitochondria became fully concentrated around the PVs. Between 24-30 h.p.i., parasite-laden macrophages ruptured, releasing the pathogens along with disintegrated mitochondria. However, despite cell lysis, the nuclei of the burst cells remained intact (Fig. 2K). In contrast, in CD11b-MΦ^β-cat⁻/⁻^ macrophages, *T. gondii* was able to enter the cells; however, the internalized parasites failed to form mature PVs (Fig. S3Ai) and were unable to replicate effectively (Fig. S3Aii), thereby failing to establish a stable intracellular niche (Fig. 2K, Video S1B). This suggests that β-catenin signaling is essential for creating a permissive intracellular environment to form PVs. We analyzed the levels of phosphorylated and native β-catenin in MΦ^β-cat-FL/FL^ and CD11b-MΦ^β-cat⁻/⁻^ macrophages. In MΦ^β-cat-FL/FL^ macrophages, β-catenin phosphorylation increased progressively, correlating with parasite growth at various post-infection time points. In contrast, CD11b-MΦ^β-cat⁻/⁻^ macrophages, derived from β-cat^ΔMΦ^ mice, exhibited markedly reduced β-catenin phosphorylation and impaired parasite replication (Fig. 2L, S3Bi,ii). CD11b-MΦ^β-cat⁻/⁻^ macrophages, derived from β-cat^ΔMΦ^ mice and lacking β-catenin, displayed significantly reduced parasite growth, confirmed through protein and RNA analyses (Fig. S3Bi,ii). Flow cytometry analysis revealed parasite infectivity rates of 17 ± 1.5% at 12 h.p.i. and 28 ± 2% at 24 h.p.i., with GFP-*T. gondii*-positive cells observed in MΦ^β-cat-FL/FL^ cells (Fig. S1L). Flow cytometry further supported these findings, showing minimal infection in CD11b-MΦ^β-cat⁻/⁻^ macrophages even at 24 h.p.i. (Fig. S1L). The flow cytometry histogram demonstrated distinct GFP-*T. gondii* positive peaks in MΦ^β-cat-FL/FL^ macrophages, indicating higher infection levels compared to the minimal infection in CD11b-MΦ^β-cat⁻/⁻^ macrophages at 12 h.p.i. Notably, infection rates in MΦ^β-cat-FL/FL^ macrophages increased 24 h.p.i., whereas CD11b-MΦ^β-cat⁻/⁻^ macrophages maintained low infection levels (Fig. S1Li). This study highlights the essential role of β-catenin signaling in supporting *T. gondii* infection by facilitating parasite replication, PVs formation, and macrophage migration via chemokine production. While β-catenin-deficient macrophages allow parasite entry, they fail to support intracellular replication, exhibit diminished chemokine (e.g., CCL19) secretion, and display increased resistance to infection. These findings offer important mechanistic insights and suggest that targeting β-catenin signaling may represent a promising therapeutic strategy to impair *T. gondii* survival and limit disease progression.

### S552 phosphorylation regulates β-catenin nuclear translocation and TCF4 interaction during infection

To determine how S552 phosphorylation regulates β-catenin function, we utilized CD11b-MΦ^β-cat⁻/⁻^ macrophages reconstituted with FLAG-tagged WT β-catenin, a phosphorylation-deficient mutant (S552A), or a phosphomimetic mutant (S552D). Co-immunoprecipitation analysis revealed that Wt-β-catenin interacts with TCF4 following *T. gondii* infection, whereas the S552A mutant failed to associate with TCF4, indicating that phosphorylation at S552 is required for β-catenin-TCF4 interaction (Fig. S2iv). Consistently, cytoplasmic and nuclear fractionation analysis by the immunoblot demonstrated that Wt-β-catenin undergoes infection-induced nuclear translocation, while the S552A mutant remained predominantly cytoplasmic. In contrast, the S552D phosphomimetic mutant exhibited constitutive nuclear localization even in the absence of infection, indicating that S552 phosphorylation is both necessary and sufficient for nuclear accumulation of β-catenin (Fig. S2v). These observations were further supported by confocal microscopy, which showed time-dependent nuclear enrichment of Wt-β-catenin upon infection, persistent cytoplasmic localization of the S552A mutant, and constitutive nuclear localization of the S552D mutant (Fig. S2vi). Together, these findings demonstrate that S552 phosphorylation regulates β-catenin nuclear translocation and its interaction with TCF4, supporting a mechanistic role in β-catenin-mediated signaling during infection.

### β-catenin-TCF4 facilitates IRF4 transcription during *T. gondii* infection

**Fig. 3:**
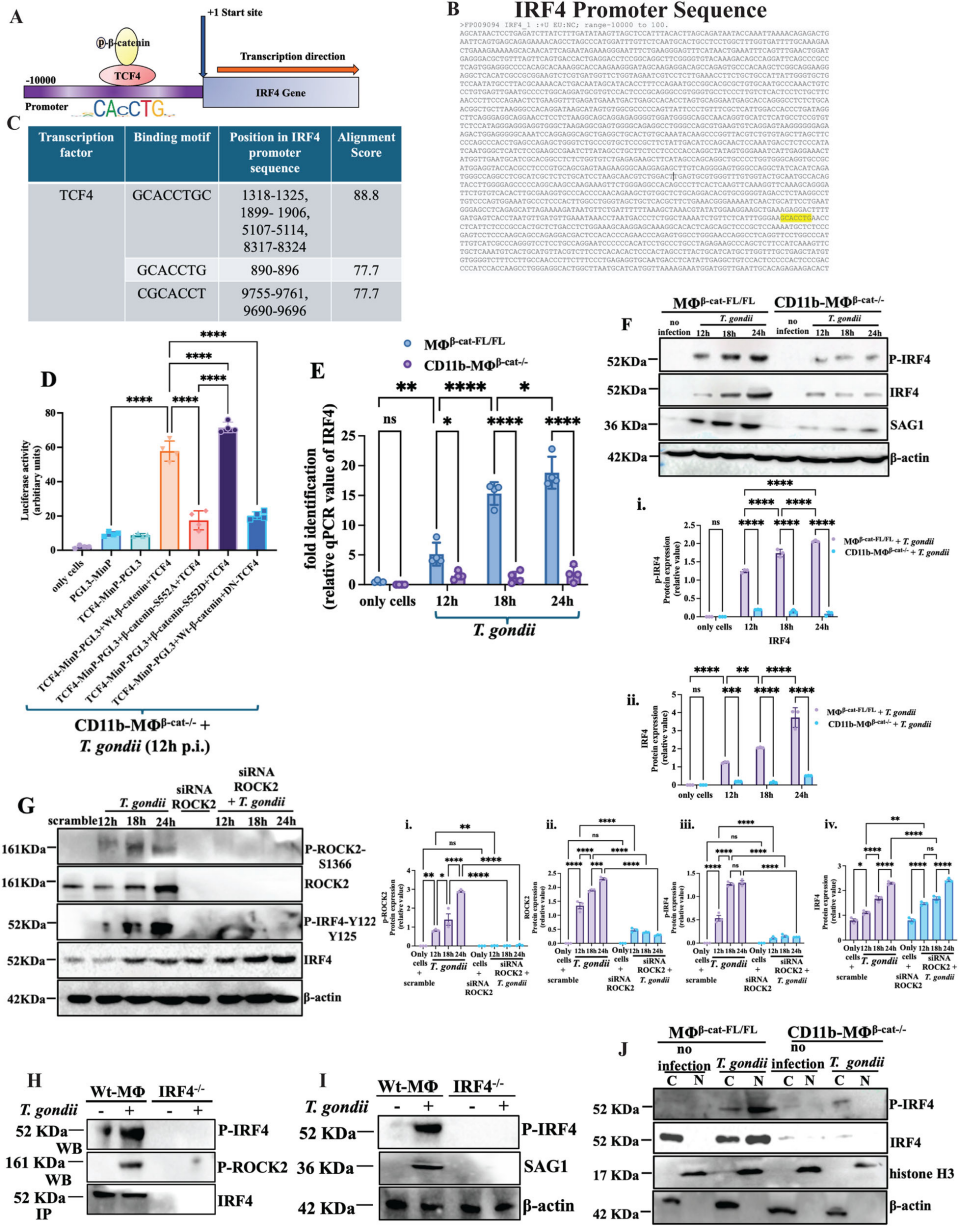
*T. gondii* infection recruits TCF4 to IRF4 promoter, and maintain its functional activity. **A** This is the hypothetical model where TCF4 could bind at the promoter region of IRF4 and regulates the transcription of IRF4. **B** IRF4 promoter sequence has multiple consensus sequences where TCF4 prefers to bind. **C** There were several consensus sequences of TCF4 with their binding site, present on IRF4 promoter site and the alignment score was calculated for individual consensus sequences. **D** TCF4-MinP-PGL3 is TCF4 binding site on IRF4 promoter with minimal promoter in pGL3 luciferase plasmid was cloned. TCF4-MinP-PGL3 was transfected with Wt-β-catenin + TCF4 plasmids, or phospho-mutant-β-catenin (S552A) + TCF4 plasmids, or phospho-mimic-β-catenin (S552D) + TCF4 plasmids or Wt-β-catenin + dominant-negative (DN)-TCF4 plasmids in CD11b-MΦ^β-cat⁻/⁻^ macrophages. pGL3-minimal promoter (PGL3-MinP) luciferase plasmid was taken as negative control and pCMV-Renilla-Luc plasmid was transfected in each condition. At 12 h post infection, cells were harvested for dual luciferase assay of TCF4-MinP-PGL3. The firefly luciferase units were normalized against the Renilla units and expressed in percentage. Data shown are the mean ± SEM (n = 3) with statistical significance assessed using one-way ANOVA. Statistical significance is indicated as ****p < 0.0001. **E** MΦ^β-cat-FL**/**FL^, and CD11b-MΦ^β-cat–/–^ macrophages were infected with RH parasites and mRNA of IRF4 was quantified by real-time PCR. Data was represented by bar diagram after having average ± SE of three individual experiments. Data shown are the mean ± SEM (*n* = 3) with statistical significance assessed using two-way ANOVA. Statistical significance is indicated as ****p < 0.0001, **p < 0.01, *p < 0.05 and “ns” indicating no significance. **F** MΦ^β-cat-FL/FL^, and CD11b-MΦ^β-cat–/–^ macrophages were infected with RH parasites and cells were harvested at different time and total lysates were subjected to immunoblotting to detect phospho-IRF4, total IRF4, SAG1, and β-actin (loading control). **i.,ii**. The average band intensities from three independent experiments were normalized to total β-actin and plotted as bar graphs for phospho-IRF4, and total IRF4. Data are presented as mean ± SE, with statistical significance indicated as ****p < 0.0001, ***p < 0.001, **p < 0.01, and “ns” for no significance. **G** Wt-MΦ macrophages from Wt mice were transected with siRNA or its scrambled version to impede the expression of ROCK2 and then infected with parasites. Cells were harvested at different time and total lysates were subjected to immunoblotting to detect phospho-ROCK2, total ROCK2, phospho-IRF4, IRF4 and β-actin (loading control). **i-iv**. The average band intensities from three independent experiments were normalized to total β-actin and plotted as bar graphs for phospho-ROCK2, ROCK2, phospho-IRF4, and total IRF4. Data shown are the mean ± SEM (n = 3) with statistical significance assessed using two-way ANOVA. Statistical significance is indicated as ****p < 0.0001, ***p < 0.001, **p < 0.01, and “ns” for no significance. **H** Wt-MΦ and IRF4^⁻/⁻^ macrophages were infected with parasites for 12 h. Total cell lysates were subjected to immunoprecipitation using a IRF4 antibody, followed by immunoblotting with a phospho-IRF4 and phospho-ROCK2 antibody. **I** Wt-MΦ and IRF4^⁻/⁻^ macrophages were infected with parasites for 12 h. Total cell lysates were subjected to immunoblot using phospho-IRF4 and SAG1 antibody. **J** MΦ^β-cat-FL/FL^, and CD11b-MΦ^β-cat–/–^ macrophages were infected with RH for 12 h.p.i and nucleus (N) and cytoplasm (C) were purified and immunoblot was done with phospho-IRF4, and total IRF4 antibody. The purity of the nucleus and cytoplasm was ascertained using histone (17 kDa) and β-actin (42 kDa) antibody respectively.

Given the existing gap in understanding the precise mechanism of β-catenin’s role in IRF4 transcription, we ask: how does β-catenin regulate IRF4 expression, and how does its inhibition affect both IRF4 transcription and *T. gondii* replication in infected macrophages? Here, we have hypothesized that the activated β-catenin-TCF complex regulates IRF4 transcription, which plays a crucial role in parasites growth by significantly modulating molecular mechanisms and the immune response within infected macrophages (Fig. 3A). JASPAR open access database revealed multiple TCF-binding sequences at the IRF4 promoter (ENSG00000137265; Fig. 3B), with the binding motif GCACCTGC/GCACCTG/CGCACCT showing an alignment score of 77-88% (Fig. 3C). To test the functionality of these sites, we constructed the TCF4-MinP-pGL3 luciferase reporter vector. CD11b-MΦ^β-cat–/–^ cells, GCACCTGC/GCACCTG/CGCACCT promoter site-dependent IRF4 transcription was abrogated, as indicated by the absence of luciferase activity from TCF4-MinP-pGL3 alone, demonstrating that without β-catenin, cellular TCF4 could not bind to the IRF4 promoter sequences (Fig. 3D). Higher promoter activity of TCF4-MinP-pGL3 was observed during transient transfection with wild-type β-catenin and TCF4, but not with S552A-mutant-β-catenin and TCF4. The phospho-mimic β-catenin(S552D) with TCF4 induced the highest luciferase activity in presence of parasites infection. Additionally, a TCF4 dominant-negative mutant suppressed GCACCTGC/GCACCTG/CGCACCT-dependent IRF4 transcription (Fig. 3D). These findings demonstrate that phosphorylation of β-catenin(S552) is essential for IRF4 gene expression. To further confirm the role of β-catenin in IRF4 transcription, we checked IRF4 mRNA levels in *T. gondii*-infected CD11b-MΦ^β-cat–/–^ cells. We found that MΦ^β-cat-FL/FL^ had increased IRF4 mRNA expression during infection, whereas IRF4 transcription was abrogated CD11b-MΦ^β-cat–/–^ cells in the presence of infection (Fig. 3E). To further confirm the essentiality of β-catenin in IRF4 expression, we infected MΦ^β-cat-FL/FL^ and CD11b-MΦ^β-cat–/–^ with parasites at different time points. We observed increased phosphorylation of IRF4 (lanes 2-4, first panel Fig. 3F,i) along with native IRF4 protein levels (lanes 2-4, second panel Fig. 3F,ii). However, in CD11b-MΦ^β-cat–/–^, both phosphorylated IRF4 (lanes 6-8, first panel Fig. 3F,i) and native IRF4 (lanes 6-8, second panel Fig. 3F,ii) were abrogated during parasite infection due to the inhibition of β-catenin. We found that phosphorylated IRF4 facilitated parasite replication (lanes 2-4, third panel, Fig. 3F), while in the absence of phospho-IRF4, parasite replication was abrogated (lanes 6-8, third panel, Fig. 3F) due to β-catenin inhibition. Taken together, these findings highlight the essential role of phospho-β-catenin in regulating IRF4 expression and facilitating parasite survival.

### ROCK2 plays a crucial role in the phosphorylation of IRF4

Having established that phosphorylated IRF4 is essential for parasite replication, we next aimed to investigate the mechanisms underlying IRF4 phosphorylation. Rho-associated coiled-coil-containing protein kinase 2 (ROCK2) is crucial for activating IRF4, a transcription factor vital for regulating immune responses and macrophage differentiation. To investigate the impact of ROCK2 on IRF4 activation, here we silenced the expression of ROCK2 gene by using its specific siRNA in Wt-MΦ. During infection, we observed increased expression of both native (lanes 2-4, second panel, Fig. 3G) and phosphorylated ROCK2 (lanes 2-4, first panel, Fig. 3G) in Wt-MΦ infected with parasites. This led to a concomitant increase in IRF4 phosphorylation (lanes 2-4, third panel, Fig. 3G) and native IRF4 levels (lanes 2-4, fourth panel, Fig. 3G) during infection. siRNA-mediated inhibition of ROCK2 transcription in Wt-MΦ suppressed its expression (lanes 6-8, second panel, Fig. 3G) and phosphorylation (lanes 6-8, first panel, Fig. 3G) in the presence of parasites (Fig. 3Gi., ii). ROCK2 inhibition impeded IRF4 phosphorylation (lanes 6-8, third panel, Fig. 3G), demonstrating that ROCK2 phosphorylation is critical for IRF4 phosphorylation (Fig. 3Giii., iv). Therefore, we conclude that ROCK2 kinase-dependent IRF4 phosphorylation is essential for its activation. Furthermore, native IRF4 expression was increased (lanes 6-8, fourth panel, Fig. 3G) under infected conditions, even with ROCK2 inhibition. This increase is likely due to β-catenin phosphorylation enhancing native IRF4 expression, suggesting that ROCK2 is involved in the posttranslational modification (phosphorylation) of IRF4, which is required for downstream signaling activation. To prove ROCK2 is essential for activating IRF4 during infection, we tested direct interaction between phopsho-IRF4 and phopsho-ROCK2, we infected with parasites with both Wt-MΦ and IRF4^⁻/⁻^ macrophages and pulled down at 12 h.p.i. with native IRF4, which was found in phosphorylated form and bound with phospho-ROCK2 (Fig. 3H). Macrophages from IRF4^⁻/⁻^ mice lacked activated ROCK2 bound to phospho-IRF4, owing to the absence of endogenous IRF4 in these cells (Fig. 3H). To assess the functional relevance of phospho-IRF4, we compared parasite replication in WT and IRF4^⁻/⁻^ macrophages. Parasite growth was markedly impaired in IRF4^⁻/⁻^ macrophages, indicating that IRF4 is essential for supporting *T. gondii* replication within host cells (Fig. 3I). Transcriptomic analysis further revealed enhanced activation of IRF4-ROCK2 signaling in parasite-infected mice compared to uninfected controls, as indicated by increased mRNA expression levels of *Irf4* and *Rock2* (Fig. S4E). Administration of XAV939 (4 mg/kg body weight) significantly suppressed the expression of these genes, both in infected and uninfected mice (Fig. S4F–H). These findings highlight the essential role of ROCK2 in IRF4 phosphorylation for downstream signaling during *T. gondii* infection.

### β-catenin is essential for the nuclear translocation of phosphorylated IRF4

β-catenin is essential for the nuclear translocation of phosphorylated IRF4 Next, we investigated the role of phosphorylated IRF4 in downstream signaling. IRF4 is a well-established transcription factor [22, 45] that must translocate to the nucleus to exert its transcriptional activity. To examine this, we purified cytoplasmic and nuclear fractions from uninfected and infected MΦ^β-cat-FL/FL^ cells and analyzed the presence of phosphorylated IRF4 in these fractions. Our findings show that 12 h.p.i, phosphorylated IRF4 is capable of translocating from the cytoplasm to the nucleus (lanes 3-4, first panel, Fig. 3J). However, when β-catenin is abrogated in CD11b-MΦ^β-cat–/–^ macrophages, this translocation of phosphorylated IRF4 is blocked, even in the presence of 12 h.p.i. (lanes 7-8, first panel, Fig. 3J). Additionally, we observed no native IRF4 expression in CD11b-MΦ^β-cat–/–^ macrophages in both absence and presence of infection, as β-catenin inhibition disrupts the transcription of IRF4 (lanes 5-8, second panel, Fig. 3J).

### IRF4 regulates CYBB transcription during *T. gondii* infection

**Fig. 4:**
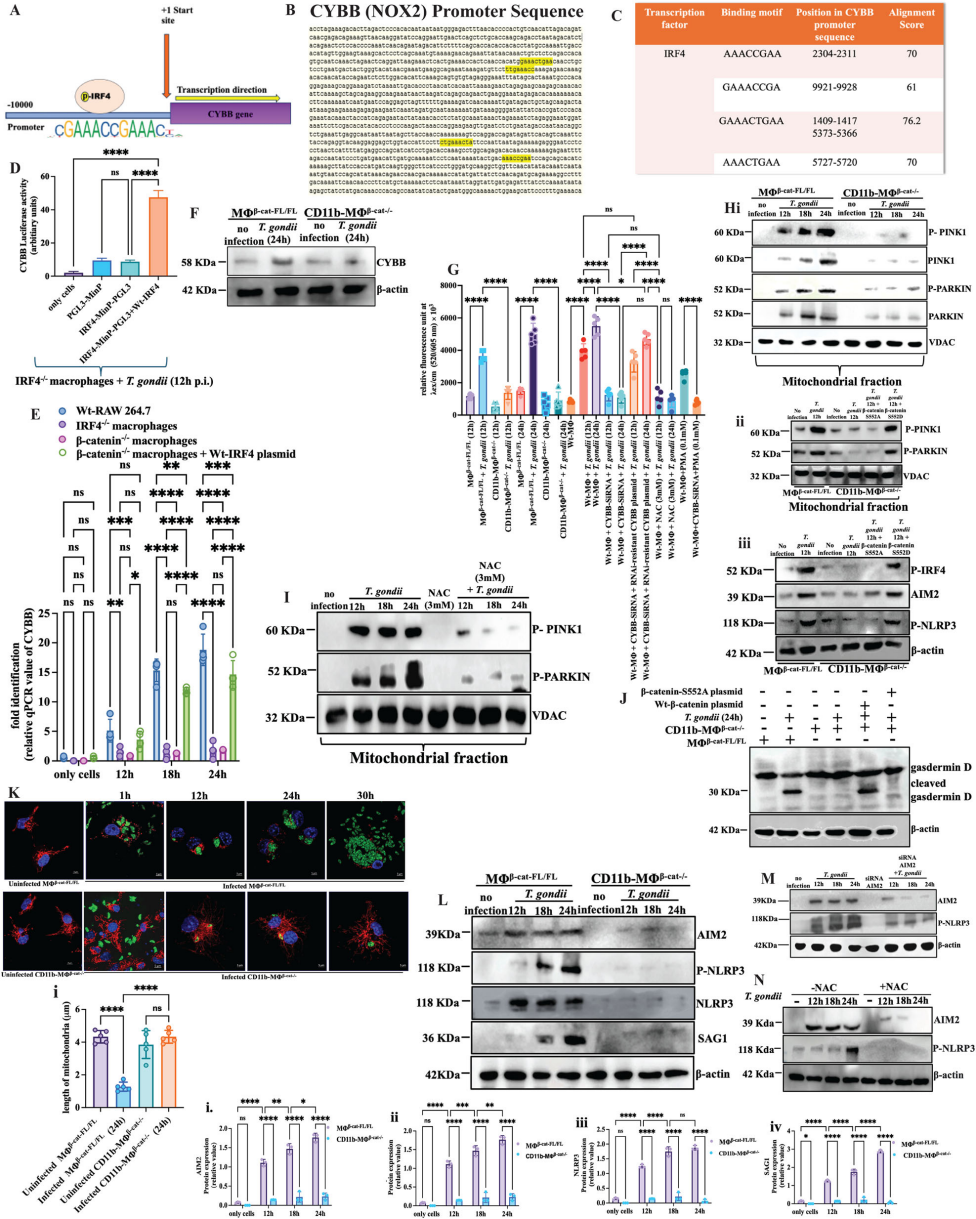
β-catenin-IRF4 reliant NOX2 (CYBB) expression determines ROS generation and aberrant mitophagy. **A** Model hypothesizing that IRF4 could bind at the promoter region of CYBB and regulate the transcription of CYBB. **B** CYBB promoter sequence showed multiple consensus sequences where IRF4 can bind. **C** There were several consensus sequences of IRF4 with their binding site, present on CYBB promoter site and the alignment score was calculated for individual consensus sequences. **D** IRF4-MinP-PGL3 is IRF4 binding site on CYBB promoter with minimal promoter in pGL3 luciferase plasmid was cloned. IRF4-MinP-PGL3 was transfected with Wt-IRF4, in IRF4^–/–^ macrophages. pGL3-minimal promoter (PGL3-MinP) luciferase plasmid was taken as negative control and pCMV-Renilla-Luc plasmid was transfected in each condition. At 12 h post infection, cells were harvested for dual luciferase assay of TCF4-MinP-PGL3. The firefly luciferase units were normalized against the Renilla units and expressed in percentage. Values were mean ± SEM from four similar experiments, statistical significance assessed using one-way ANOVA. Statistical significance is indicated as ****p < 0.0001 and “ns” for no significance. **E** Wt-RAW 264.7 cells, IRF4^–/–^, β-catenin^–/–^, and β-catenin^–/–^ macrophages overexpressing with wild-type IRF4 were infected with parasites. Cells were harvested and mRNA of CYBB was quantified by real-time PCR. Data shown are the mean ± SEM (*n* = 3) with statistical significance assessed using two-way ANOVA. Statistical significance is indicated as ****p < 0.0001, ***p < 0.001, **p < 0.01, *p < 0.05 and “ns” indicating no significance. **F** MΦ^β-cat-FL/FL^ and CD11b-MΦ^β-cat⁻/⁻^ cells were infected with parasites for 24 h, and total lysates were subjected to immunoblotting to detect CYBB and β-actin (loading control). **G** MΦ^β-cat-FL/FL^ and CD11b-MΦ^β-cat⁻/⁻^ cells were infected with parasites along with Wt-MΦ that were transfected with either scramble or CYBB-specific siRNA or with an siRNA-resistant CYBB construct in siRNA-knockdown cells. In parallel, Wt-MΦ were treated with PMA in the presence or absence of CYBB-specific siRNA for 12 h, and separately, cells were treated with NAC either with or without infection. After 12 and 24 h of infection, levels of ROS, measured as released H₂O₂, were quantified spectrophotometrically. Data represent mean ± SEM (*n* = 5), and statistical significance was evaluated using one-way ANOVA. Statistical significance is indicated as ****p < 0.0001 and expressed in the relative fluorescent unit (RFU), measured at λex/em 520/605 nm. **H**i MΦ^β-cat-FL/FL^ and CD11b-MΦ^β-cat⁻/⁻^ cells were infected with parasites for various durations. Followed by, Mitochondrial fractions were purified from total cell lysates, and total mitochondrial extracts were used for immunoblotting to detect phospho-PINK1, PINK1, phospho-PARKIN, PARKIN, and VDAC (loading control). (ii, iii) Immunoblot analysis of phospho-PINK1, phospho-PARKIN, phospho-IRF4, AIM2, and phospho-NLRP3 in macrophages derived from MΦ^β-cat-FL/FL^ and CD11b-MΦ^β-cat⁻/⁻^ mice under uninfected conditions or following *T. gondii* infection (12 h). β-catenin-deficient macrophages were reconstituted with either wild-type β-catenin, phosphorylation-deficient mutant (S552A), or phosphomimetic mutant (S552D). **I** Wt-MΦ were infected with parasites at different time points, with or without NAC treatment. Mitochondrial fractions were isolated from total cell lysates, and the resulting mitochondrial extracts were subjected to immunoblotting to detect phospho-PINK1, total PINK1, phospho-PARKIN, total PARKIN, and VDAC (used as a loading control). **J** MΦ^β-cat-FL/FL^ and CD11b-MΦ^β-cat⁻/⁻^ cells were infected with parasites for 12 h, with or without the presence of either the Wt-β-catenin plasmid or the phospho-mutant-β-catenin (S552A) plasmid. Following infection, the cells were harvested and subjected to immunoblotting to analyze gasdermin D cleavage for pyroptosis evaluation. **K** MΦ^β-cat-FL/FL^ and CD11b-MΦ^β-cat⁻/⁻^ cells were infected with the GFP-RH strain of *T. gondii* and, at specified time points, stained with MitoTracker (red) for mitochondria and DAPI (blue) for nuclei. Confocal microscopy was used for visualization, with scale bars indicating 5 μm. **i**. The length of each mitochondrion was measured using Fiji software, and the average mitochondrial length was quantified and presented as a bar graph based on data from five independent experiments. Data shown are the mean ± SEM (*n* = 5) with statistical significance assessed using two-way ANOVA. Statistical significance is indicated as ****p < 0.0001 and “ns” for not significant. **L** MΦ^β-cat-FL/FL^ and CD11b-MΦ^β-cat⁻/⁻^ cells were infected with parasites for different time points, and total cellular lysates were subjected to immunoblotting to detect AIM2, phospho-NLRP3, NLRP3, SAG1 and β-actin (loading control). i-iv. The average band intensities from three independent experiments were normalized to total β-actin and plotted as bar graphs for AIM2, phospho-NLRP3, total NLRP3, and SAG1. Data shown are the mean ± SEM (*n* = 3) with statistical significance assessed using two-way ANOVA. Statistical significance is indicated as ****p < 0.0001, ***p < 0.001, **p < 0.01, *p < 0.05 and “ns” for no significance. **M** Wt-MΦ transfected with either scramble or AIM2-specific siRNA were infected with parasites at different time points. Total cellular lysates were then collected and analyzed by immunoblotting to detect AIM2, phospho-NLRP3, and β-actin (used as a loading control). **N** Wt-MΦ cells were infected with parasites for different time points in the presence of NAC and total cellular lysates were subjected to immunoblotting to detect AIM2, phospho-NLRP3 and β-actin (loading control).

The cytochrome b-558 beta-subunit (CYBB; also known as NOX2- a subunit of the NADPH oxidase enzyme complex) is an enzyme complex involved in the production of ROS in phagocytes, playing a key role inflammatory diseases [25, 46]. Here, we aim to investigate how IRF4 as a transcription factor regulates CYBB gene expression during *T. gondii* infection and whether this process is independent of β-catenin signaling. This question directly addresses the mechanisms and dependencies observed in our experimental results, specifically regarding the role of IRF4 in regulating CYBB expression and its interaction with β-catenin signaling during the infection. We hypothesized that phosphorylated IRF4 (p-IRF4) might facilitate *T. gondii* replication by upregulating CYBB gene expression (Fig. 4A). Through the JASPAR open-access database, we identified several IRF4-binding sequences within the CYBB promoter region (ENSG00000165168; Fig. 4B), with the binding motifs AAACCGAA/GAAACCGA/AAACTGAA showing an alignment score of 60-80% (Fig. 4C). To evaluate the functionality of these sites, we constructed an IRF4-MinP-pGL3 luciferase reporter vector. In IRF4-deficient (IRF4^–/–^) macrophages, CYBB transcription at the AAACCGAA/GAAACCGA/AAACTGAA sites was abolished, as indicated by the absence of luciferase activity from the IRF4-MinP-pGL3 vector alone. This confirmed that without IRF4, CYBB transcription cannot be initiated (Fig. 4D). In contrast, transient transfection with wild-type IRF4 resulted in strong promoter activity, reinforcing the critical role of IRF4 in CYBB gene expression (Fig. 4D). To further verify the essentiality of β-catenin in IRF4-mediated CYBB expression, we infected Wt-RAW 264.7 macrophages, IRF4^–/–^ macrophages, β-catenin^–/–^ macrophages, and β-catenin^–/–^ macrophages overexpressing wild-type IRF4 (Wt-IRF4 plasmid) with *T. gondii* at various time points (Fig. 4E). After end of the infections, cells were harvested and CYBB mRNA levels were quantified using real-time PCR. While wild-type macrophages exhibited an increase in CYBB mRNA expression during infection, but absent in both IRF4^–/–^ and β-catenin^–/–^ macrophages. Importantly, β-catenin^–/–^ macrophages overexpressing Wt-IRF4 displayed significant CYBB transcription after infection, demonstrating that IRF4 is directly required for CYBB expression (Fig. 4E) during *T. gondii* infection, with phosphorylation of IRF4 facilitating this process.

While CYBB expression is independent of β-catenin signaling, β-catenin remains essential for IRF4 expression, highlighting a hierarchical regulatory mechanism. This is because β-catenin acts upstream of IRF4-CYBB activation. However, without β-catenin, IRF4 expression is reduced, resulting in the suppression of CYBB expression. Thus, in the absence of β-catenin, CYBB suppression naturally occurs unless IRF4 is externally overexpressed.

### β-catenin reliant CYBB expression augments ROS generation in cells

We would like to investigate how β-catenin, CYBB, and ROS interact to regulate the host immune response in phagocytic cells, such as macrophages, during *T. gondii* infection. The gap lies in understanding the exact mechanisms by which β-catenin regulates CYBB and ROS during *T. gondii* infection, including their roles in immune modulation, balancing ROS production, and their crosstalk in chronic infection. To decipher this, in the experimental setup, MΦ^β-cat-FL/FL^ and CD11b-MΦ^β-cat–/–^ macrophage cells were infected with parasites for 24 h and the total cell lysate was subjected to immunoblotting to detect CYBB. In MΦ^β-cat-FL/FL^ macrophages, the expression of CYBB significantly increased at 24 h.p.i (Fig. 4F). CYBB contributes to elevated ROS production. ROS levels were assessed using a spectrophotometric assay to measure released H₂O₂. For this MΦ^β-cat-FL/FL^ and CD11b-MΦ^β-cat–/–^ cells infected with parasites for 12 h and 24 h were subjected to measurement of ROS level. Following the photodynamic protocol, ROS levels in uninfected this, MΦ^β-cat-FL/FL^ cells remained at baseline at 12 h and remained stable through 24 h. While MΦ^β-cat-FL/FL^ cells showed enhanced ROS levels were detected as early as 12 h.p.i, peaking at 24 h.p.i. (Fig. 4G), When CYBB expression was markedly upregulated (Fig. 4F). In uninfected CD11b-MΦ^β-cat–/–^ macrophages, there was no increase in ROS production at either 12 or 24 h (Fig. 4G), due to suppressed CYBB expression (Fig. 4F). Subsequently, ROS levels in uninfected MΦ^β-cat-FL/FL^ cells remained at baseline at 12 h and remained stable through 24 h. However, in infected CD11b-MΦ^β-cat–/–^ macrophages, ROS levels were significantly lower at 12 and 24 h compared to infected MΦ^β-cat-FL/FL^ macrophages (Fig. 4G), which was also associated with reduced CYBB expression (Fig. 4F).

To investigate the functional role of CYBB in ROS generation during infection, we infected Wt-MΦ for 12 h and 24 h and observed a significant increase in ROS levels compared to uninfected controls in presence of scramble siRNA (tenth, eleventh bar, Fig. 4G). However, when CYBB expression was silenced using siRNA, infected macrophages failed to exhibit any increase in ROS production (twelfth, thirteen bar, Fig. 4G), however, re-expression of an siRNA-resistant CYBB construct in knockdown cells restored and enhanced ROS production at both 12 and 24 h.p.i. (fourteenth, fifteenth bar, Fig. 4G), indicating that CYBB is a key regulator of infection-induced ROS generation (Fig. 4G). Furthermore, treatment with N-acetylcysteine (NAC), a well-established ROS scavenger(52), effectively suppressed the infection-induced ROS burst (sixteenth, seventeenth bar, Fig. 4G), confirming that the observed ROS signal was specifically linked to oxidative stress. We also used PMA as a well-validated CYBB-dependent ROS inducer (positive control) [47] to directly stimulate ROS production in Wt-MΦ at 12 h of incubation, which caused a robust increase in ROS levels (eighteenth bar, Fig. 4G). Because PMA-driven ROS generation depends on CYBB, we knocked down CYBB using siRNA and then stimulated the cells with PMA. Under these conditions, PMA failed to elevate ROS levels (nineteenth bar, Fig. 4G), confirming that the loss of ROS induction resulted from CYBB suppression by siRNA. When this PMA-induced ROS level was compared with that of *T. gondii*-infected cells treated with CYBB siRNA, the marked reduction observed (twelfth, thirteen bar, Fig. 4G) confirmed that the decrease in ROS was specifically attributable to CYBB knockdown. These findings collectively demonstrate that CYBB is essential for ROS production in macrophages during infection, and that ROS accumulation can be pharmacologically attenuated, thereby validating the functional specificity of CYBB-mediated oxidative responses. These findings suggest that β-catenin drives the upregulation of CYBB expression, leading to increased ROS production, which plays a crucial role in mitophagy.

### Mitochondrial dysfunction leads to mitophagy

Elevated levels of ROS serve as a signal to trigger mitophagy, a process that selectively degrades damaged mitochondria within the cell [48]. The molecular processes driving the initiation and regulation of mitophagy are complex, involving a network of proteins and signaling pathways that facilitate the selective degradation of damaged mitochondria due to excess ROS generation. Notably, two core molecular players pivotal for mitophagy activation, PTEN-induced kinase 1 (PINK1) and PARKIN, are involved in this process. ROS promotes mitophagy by stabilising PINK1 on the outer mitochondrial membrane of phagocytes, leading to the recruitment of PARKIN, which then ubiquitinates mitochondrial proteins and facilitates mitophagosome formation. Subsequently, leading to the malfunction of mitochondria [49]. The exact mechanisms through which mitophagy contributes to *T. gondii* infection remain unclear. Specifically, it is not well understood how defective or dysregulated mitophagy influences parasite survival, immune responses, and host cell integrity during infection. To elucidate this, MΦ^β-cat-FL/FL^ and CD11b-MΦ^β-cat–/–^ macrophages were infected with *T. gondii* for varying durations, and mitochondrial fractions were purified and analyzed for the expression of mitophagy markers PINK1 and PARKIN. In the mitochondrial fraction of MΦ^β-cat-FL/FL^ macrophages, we observed a significant upregulation of phospho-PINK1 during parasite infection (lanes 2-4, first panel, Fig. 4Hi), accompanied by a concomitant increase in phospho-PARKIN expression (lanes 2-4, third panel, Fig. 4Hi). However, in the absence of β-catenin, the mitochondrial fraction of CD11b-MΦ^β-cat–/–^ macrophages showed no detectable phosphorylation of PINK1 (lanes 6-8, first panel, Fig. 4Hi) and only minimal expression of phospho-PARKIN during infection (lanes 6-8, third panel, Fig. 4Hi). To determine whether β-catenin phosphorylation is required for PINK1-PARKIN activation, we examined β-catenin-deficient macrophages reconstituted with phosphorylation mutants. Reconstitution with the phosphorylation-deficient S552A mutant failed to rescue PINK1 and PARKIN activation, whereas expression of the phosphomimetic S552D mutant restored PINK1-PARKIN signaling (Fig. 4Hii). These findings demonstrate that phosphorylation of β-catenin is essential for the induction of phospho-PINK1 and phospho-PARKIN during parasite infection, thereby promoting mitophagy in macrophages. The absence of β-catenin disrupts this pathway, underscoring its critical role in regulating mitochondrial quality control and host immune responses in infected cells. To clarify the upstream signals regulating mitophagy during *T. gondii* infection, we investigated whether CYBB-mediated ROS production is essential for PINK1 and PARKIN activation. Since ROS is known to trigger mitochondrial stress and initiate PINK1/PARKIN-dependent mitophagy [49, 50], we hypothesized that its inhibition would disrupt this pathway. Wt-MΦ macrophages were infected with *T. gondii* and treated with NAC, at various time points, and mitochondrial fraction was purified. NAC treatment markedly reduced phosphorylation of PINK1 and PARKIN, indicating that ROS is required for their activation (Fig. 4I). These results establish that CYBB-driven ROS is a key upstream signal for PINK1/PARKIN activation during infection.

### β-catenin is essential for mitophagy induction in *T.gondii-*infected macrophages

To determine whether β-catenin signaling regulates mitophagy during *T. gondii* infection, we performed confocal imaging using the mitophagy detection kit (Dojindo Molecular Technologies Inc.) [51], which labels mitophagic vesicles with a red fluorescent dye. Infected MΦ^β-cat-FL/FL^ macrophages displayed a strong mitophagy response at early time points (6 h.p.i.), as indicated by intense red fluorescence colocalizing with intracellular parasite vacuoles (green) and nuclei (blue) (Fig. S3C, left panel). However, by 12 h.p.i., this red signal noticeably declined, suggesting a progressive reduction in mitophagy activity over time. This temporal pattern implies that mitophagy is actively engaged during the early phase of infection to clear damaged mitochondria, but becomes insufficient as the infection progresses. In contrast, CD11b-MΦ^β-cat–/–^macrophages exhibited minimal red fluorescence at all time points, indicating a marked impairment of mitophagy in the absence of β-catenin signaling (Fig. S3C, right panel). These findings support the conclusion that β-catenin is essential for initiating mitophagy in response to *T. gondii* infection, particularly during the early stages.

### β-catenin phosphorylation at S552 is essential for gasdermin D-mediated pyroptosis in *T. gondii*-infected macrophages

Mitophagy is typically a protective mechanism that removes damaged mitochondria to maintain cellular homeostasis. However, when mitophagy appears insufficient to counter sustained mitochondrial damage and elevated ROS levels, then the cell switches to pyroptosis as a last-resort defense mechanism, promoting inflammation [52] However, the mechanisms connecting mitophagy and pyroptosis in *T. gondii* infection remain poorly understood. Additionally, it sought to determine how β-catenin-dependent signaling influences mitochondrial integrity, parasite replication, and cellular fate during the course of infection. This part of the study aims to link the importance of β-catenin to the gasdermin D-mediated pyroptosis signaling axis. To investigate this, MΦ^β-cat-FL/FL^ and CD11b-MΦ^β-cat–/–^ cells were infected with parasites for 12 h with or without the presence of either the Wt-β-catenin plasmid or the phospho-mutant-β-catenin (S552A) plasmid and immunoblotting for gasdermin D was performed. Western blot analysis revealed that, in MΦ^β-cat-FL/FL^ macrophages, *T. gondii* infection induced gasdermin D cleavage, indicative of pyroptosis (lanes 2, Fig. 4J). In contrast, CD11b-MΦ^β-cat–/–^ macrophages showed no gasdermin D cleavage upon infection, indicating an absence of pyroptosis (lanes 4, Fig. 4J). However, transfection of CD11b-MΦ^β-cat–/–^ cells with Wt-β-catenin plasmid restored gasdermin D cleavage following infection (lanes 5, Fig. 4J). Notably, transfection with the phospho-mutant β-catenin-S552A failed to induce gasdermin D cleavage in CD11b-MΦ^β-cat–/–^ cells upon infection (lanes 6, Fig. 4J). These findings entail that β-catenin is critical for gasdermin D-mediated pyroptosis in *T. gondii*-infected macrophages and that its phosphorylation at S552 is essential for this process. Further, we aim to understand how β-catenin-dependent signaling influences mitochondrial integrity and cellular fate in *T. gondii*-infected macrophages. We therefore investigated whether β-catenin in infected macrophages improves mitochondrial morphology by staining mitochondria (MitoTracker, red), the nucleus (DAPI, blue), and parasites (GFP), followed by confocal microscopy analysis. Confocal microscopy analysis revealed that in the early stages of infection (1 h), mitochondria were dispersed throughout the cytoplasm (Fig. 4K), exhibiting an elongated morphology with an average length of 4 ± 1 µm (Fig. 4Ki). By 6 h.p.i., mitochondria began clustering near regions where parasites initiated PV formation (Fig. 4K). Between 12 and 24 h.p.i., mitochondria localized around the periphery of PVs, adopting a rounded morphology with an average length of less than 1 ± 0.5 µm (Fig. 4Ki). At later stages of infection (24-30 h.p.i.), the continuous network of mitochondria previously observed surrounding the PV was presented as fragmented mitochondria (Video S1C). The cells overloaded with parasites ruptured, leading to mitochondrial disintegration while the nucleus remained intact, a hallmark of pyroptosis (Fig. 4K, Video S1C). Similar findings were observed through live imaging, where mitochondria were localized around the PV periphery during earlier stages and, at later stages, parasite egress was accompanied by mitochondrial disintegration and an intact nucleus (Video S1C). Whereas, in CD11b-MΦ^β-cat–/–^ cells, parasite infection did not compromise mitochondrial integrity (Fig. 4K), and the mitochondria retained their elongated structure with an average length of 4 ± 1 µm at 24 h.p.i. (Fig. 4Ki). GFP-tagged parasites were unable to multiply or form mature PVs (Fig. 4K). In summary, confocal microscopy and live imaging demonstrated that mitochondria clustered around PVs during early infection stages but disintegrated as cells ruptured in later stages with an intact nucleus, indicating pyroptosis. In CD11b-MΦ^β-cat–/–^ cells, mitochondrial integrity remained intact, and parasites were unable to multiply or form mature PVs. Thus, we can conclude that β-catenin phosphorylation is essential for gasdermin D-mediated pyroptosis in *T. gondii*-infected macrophages. Our data demonstrate that both mitophagy at early time points (Fig. 4H, I, S3C) and GSDMD-mediated pyroptosis at the late stage of infection (Fig. 4J, K, Video S1C) are activated in *T. gondii*-infected macrophages. This co-occurrence suggests a multi-phased host response, where mitophagy is initially engaged to alleviate mitochondrial stress and maintain cellular homeostasis, potentially creating a permissive environment for parasite replication within PVs (Video S1A). However, with persistent infection, virulent nature of RH strain, or excessive mitochondrial damage due to higher ROS, mitophagy becomes insufficient to contain cellular stress, leading to inflammasome activation and pyroptotic cell death. The induction of pyroptosis (Fig. 4K, 30 h.p.i.; Video S1C) may, in turn, facilitate parasite egress (Video S1A;24-30 h.p.i.) and dissemination. The simultaneous detection of mitophagy and pyroptosis within the same cells implies a temporal or threshold-dependent switch from a protective to a pro-inflammatory response, highlighting the dynamic nature of host-pathogen interactions during *T. gondii* infection.

### β-catenin promotes parasite growth by enhancing AIM2 and NLRP3 inflammasome activation

Up to this point, we have demonstrated that β-catenin promotes elevated ROS levels, which play a crucial role in initiating mitophagy. However, when mitophagy is impaired or overwhelmed by excessive mitochondrial damage, it can lead to pyroptosis, an inflammatory form of cell death (Fig. 4). Moreover, sustained mitochondrial stress, excessive ROS, and mitochondrial damage are also known to oxidize mitochondrial DNA and activate the AIM2/NLRP3 inflammasome [47, 53, 54] multiprotein complexes [55–57]. AIM2 is preferred when cytosolic DNA, including mtDNA, serves as the primary danger signal, whereas NLRP3 is activated in response to mitochondrial stress, metabolic stress, or ion flux changes [58]. We aim to investigate how β-catenin-mediated ROS production activates AIM2 and NLRP3 inflammasomes and the role of oxidized mitochondrial DNA in modulating immune responses during *T. gondii* infection. We hypothesize that enhanced ROS and a pyroptotic feedback loop contribute to this process. In infected cells, mtDNA becomes oxidized due to enhanced ROS. This oxidized mtDNA subsequently activates AIM2 which facilitates NLRP3-ASC-Caspase-1 inflammasome complex formation. As a result, caspase-1 is activated, leading to IL-1β processing. Our study aims to investigate how β-catenin modulates the activation of these inflammasome complexes, highlighting its critical role in infection and immune regulation. To test that, we infected both MΦ^β-cat-FL/FL^ and CD11b-MΦ^β-cat–/–^ macrophages and checked the fate of inflammasome complex. In MΦ^β-cat-FL/FL^ macrophages, we observed higher expression of AIM2 (lanes 2-4, first panel, Fig. 4L,i) and increased phosphorylation of NLRP3 (lanes 2-4, second panel, Fig. 4L,ii,iii), which supported parasite growth (lanes 2-4, fourth panel, Fig. 4L,iv). In contrast, CD11b-MΦ^β-cat–/–^ macrophages did not exhibit significant upregulation of AIM2 (lanes 6-8, first panel, Fig. 4L,i) or phosphorylation of NLRP3 (lanes 6-8, second panel, Fig. 4L,ii,iii). This was concomitant with the abrogation of parasite infection (lanes 6-8, fourth panel, Fig. 4L,iv) due to the absence of β-catenin. Hence, in summary the absence of β-catenin in CD11b-MΦ^β-cat–/–^ macrophages suppresses AIM2 activation, NLRP3 phosphorylation, and parasite growth, highlighting β-catenin’s role in AIM2 and NLRP3 reliant inflammasome activation supporting infection (Fig. 4L). To determine whether β-catenin phosphorylation is required for AIM2 activation and NLRP3 phosphorylation, we examined β-catenin-deficient macrophages reconstituted with phosphorylation mutants. Reconstitution with the phosphorylation-deficient S552A mutant failed to restore AIM2 expression, IRF4 activation, and optimal NLRP3 phosphorylation, whereas expression of the phosphomimetic S552D mutant rescued these signaling events (Fig. 4Hiii). These findings demonstrate that phosphorylation of β-catenin is essential for the induction of AIM2 and activation of NLRP3 during parasite infection, thereby promoting inflammasome assembly in macrophages. To investigate whether NLRP3 inflammasome activation depends on AIM2 during parasite infection, we performed siRNA-mediated knockdown of AIM2. Western blot analysis showed that AIM2 knockdown markedly reduced phosphorylated NLRP3 levels following infection, demonstrating that AIM2 activation is associated with optimal NLRP3 phosphorylation and subsequent inflammasome activation in this setting (Fig. 4M). Furthermore, since we propose that ROS plays an essential role in inflammasome activation, we suppressed ROS production using NAC. Inhibition of ROS significantly impaired AIM2 activation and NLRP3 phosphorylation after parasite infection, confirming that ROS is indispensable for AIM2 activation and subsequent inflammasome activation in this context (Fig. 4N).

### Oxidized mitochondrial DNA activates AIM2

After examining the role of β-catenin in ROS-driven AIM2 and NLRP3 inflammasome activation during *T. gondii* infection, we aimed to decipher the activation pathway of these inflammasomes and assess their impact on immune responses and parasite replication. To investigate the role of β-catenin in ROS-driven activation of AIM2 and NLRP3 inflammasomes during *T. gondii* infection and to understand how mtDNA oxidation influences immune responses and parasite replication, we conducted a series of experiments. To determine the source and extent of oxidative DNA damage induced by *T. gondii* infection, we quantified 8-hydroxy-2′-deoxyguanosine (8-OHdG) levels in cytoplasmic DNA (cDNA), nuclear DNA (nDNA), and mitochondrial DNA (mtDNA) isolated from infected and uninfected MΦ^β-cat-FL/FL^ and CD11b-MΦ^β-cat–/–^ macrophages. In MΦ^β-cat-FL/FL^ macrophages, infection for 12 and 24 h led to a pronounced increase in 8-OHdG within the cytoplasmic DNA fraction (Fig. S3D), attributable to the leakage of oxidized mtDNA from mitochondria into the cytoplasm (Fig. S3E), while the nuclear DNA fraction not found in cytoplasm (Fig. S3E). At 12 h.p.i., the presence of 8-OHdG was higher in the mitochondria than in the cytoplasm because the mitochondria had not yet undergone complete disintegration. As a result, fewer oxidized mtDNA traces were detectable in the cytoplasm at this early stage of infection. In contrast, at 24 h.p.i., the release of oxidized mtDNA into the cytoplasm increased substantially, coinciding with marked mitochondrial disintegration at the later stage of infection (Fig. S3D). This selective release of oxidized mtDNA may provide the necessary substrate for AIM2 inflammasome activation. In contrast, CD11b-MΦ^β-cat–/–^ macrophages exhibited no significant elevation of 8-OHdG in any DNA compartment, with both infected 12 and 24 h.p.i. and uninfected groups (Fig. S3D), maintaining uniformly low oxidative damage in cDNA, nDNA, and mtDNA (Fig. S3D), underscoring the protective role of β-catenin deletion in limiting *T. gondii*-induced mitochondrial oxidative stress.

To directly trace the release of mtDNA into the cytoplasm, we performed qPCR analysis of cytoplasmic DNA (Fig. S3E). Infected MΦ^β-cat-FL/FL^ macrophages showed significant enrichment of mitochondrial genes ND1 and ND5 compared to uninfected controls, whereas nuclear gene HK2 expression remained unchanged (Fig. S3E). Importantly, no significant amplification was detected with HK2 nuclear primers in the cytoplasmic fraction, confirming the absence of nuclear DNA contamination. This selective enrichment of mitochondrial sequences in the cytoplasm demonstrates their origin from mitochondria. By contrast, CD11b-MΦ^β-cat–/–^ macrophages displayed only negligible levels of cytoplasmic mtDNA following infection, further establishing that β-catenin signaling is essential for infection-driven release of oxidized mtDNA from mitochondria into the cytoplasm (Fig. S3D, E). Collectively, these findings establish that the oxidative DNA damage observed in infected wild-type macrophages arises predominantly from mitochondria and that β-catenin is a critical regulator of this process.

**Fig. 5:**
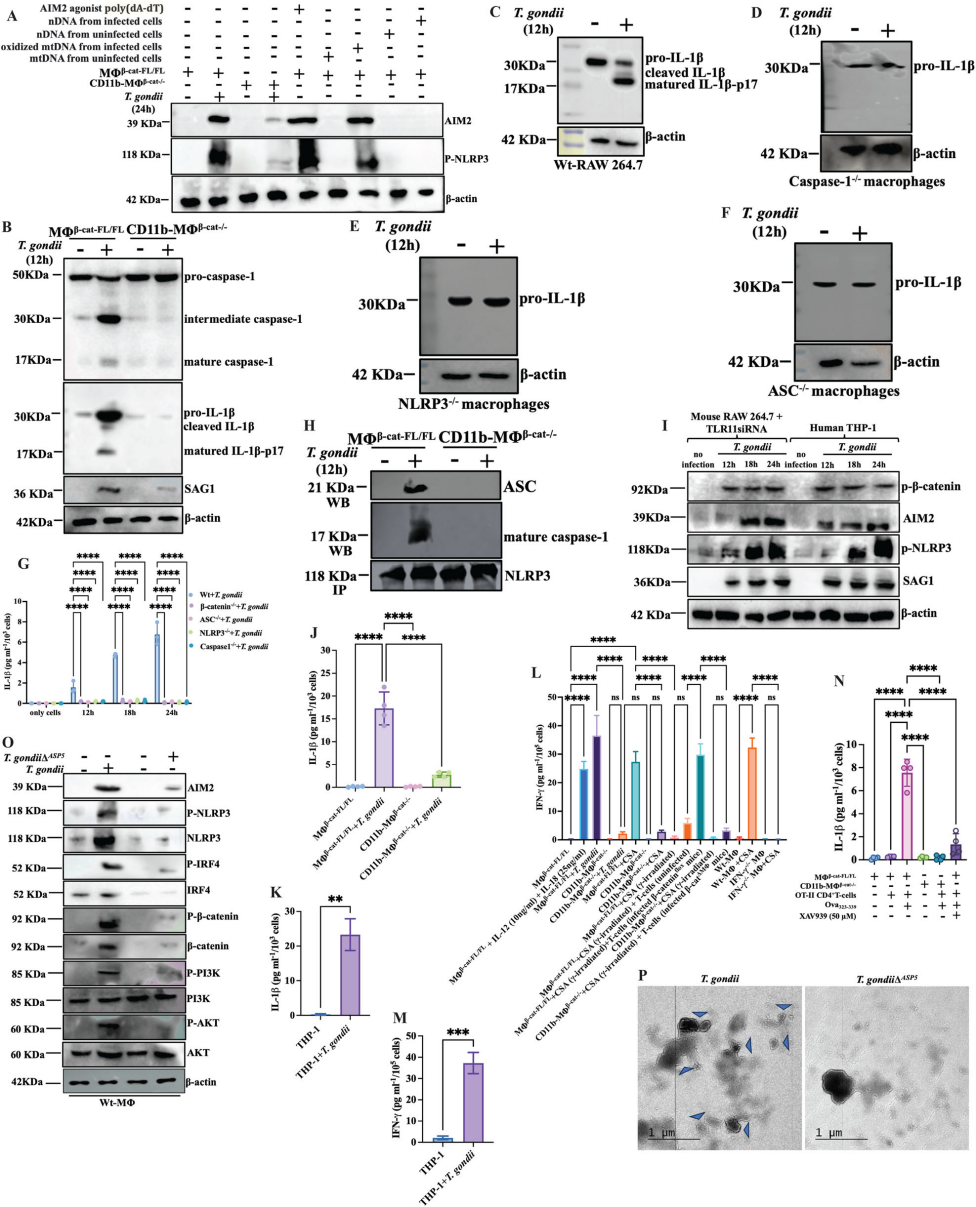
*T. gondii* infection induces β-catenin dependent AIM2-NLRP3-Casapse-1-IL1β inflammasome complex. **A** MΦ^β-cat-FL/FL^ and CD11b-MΦ^β-cat⁻/⁻^ cells were either infected with *T. gondii* for 24 h or transfected with nuclear or mitochondrial DNA purified from uninfected or infected MΦ^β-cat-FL/FL^ cells into Wt-MΦ. Total cellular lysates were then analyzed by immunoblotting to detect AIM2, phospho-NLRP3, and β-actin (loading control). **B** MΦ^β-cat-FL/FL^ and CD11b-MΦ^β-cat⁻/⁻^ cells were infected with parasites for 12 h, and the formation of mature caspase-1 and mature IL-1β, along with parasite growth (SAG1), was analyzed by immunoblotting**. C**–**F** Wild-type RAW 264.7 cells, along with caspase-1^–/–^, ASC^–/–^, and NLRP3^–/–^ variants, were infected, and the processing of IL-1β into its mature form was analyzed using immunoblotting. **G** Supernatants from infected wild-type RAW 264.7, β-catenin^–/–^, caspase-1^–/–^, ASC^–/–^, or NLRP3^–/–^ cells were collected, and bioactive IL-1β levels were quantified using ELISA. Data was represented by bar graph from three independent experiments. Error bars represent mean ± SEM, with statistical significance (two-way ANOVA) indicated as ****p < 0.0001. **H** Immunoprecipitation was performed using an NLRP3 antibody on MΦ^β-cat-FL/FL^ and CD11b*-*MΦ^β-cat⁻/⁻^ cells in the presence or absence of infection, followed by immunoblotting to detect ASC (21 kDa) and mature caspase-1 (17 kDa). **I** TLR11 knockdown mouse RAW 264.7 and human THP-1 macrophages were infected with *T. gondii* at different time points, and total cellular lysates were subjected to immunoblot analysis to detect phospho-β-catenin, AIM2, phospho-NLRP3, and β-actin (used as a loading control). **J, K** MΦ^β-cat-FL/FL^, CD11b-MΦ^β-cat⁻/⁻^, and THP-1 macrophages were cultured with or without parasites infection. Supernatants were then collected, and bioactive IL-1β levels (mouse and human separate kit) were measured using ELISA. Data was represented by bar graph from four independent experiments. Error bars represent mean ± SEM, with statistical significance (one-way ANOVA) indicated as ****p < 0.0001 and “ns” for not significant. **L, M** MΦ^β-cat-FL/FL^ and CD11b-MΦ^β-cat⁻/⁻^ macrophages were cultured in the presence or absence of parasites infection or complete soluble antigens (CSA) derived from *T. gondii* lysates. Additionally, MΦ^β-cat-FL/FL^ macrophages were incubated with a combination of IL-12 and IL-18 for 12 h as positive control. Following incubation, culture supernatants were collected, and bioactive IFN-γ levels were quantified by ELISA. Additionally, MΦ^β-cat-FL/FL^ and CD11b-MΦ^β-cat⁻/⁻^ macrophages, with CSA were further γ-irradiated and co-cultured with T-cells isolated from the splenocytes of either β-catenin^flox^ and β-cat^ΔMΦ^ -infected mice. In parallel, Wt-MΦ and BMDMs from IFN-γ^⁻/⁻^ mice were incubated with CSA, and THP-1 was infected with parasites for 12 h and culture supernatants were collected. Bioactive mouse and human-specific IFN-γ levels from supernatants were measured using ELISA. Data was represented by bar graph from four independent experiments. Error bars represent mean ± SEM, with statistical significance (one-way ANOVA) indicated as ****p < 0.0001 and “ns” for not significant. **N** IL-1β levels were quantified by ELISA in the supernatants of MΦ^β-cat-FL/FL^ and CD11b-MΦ^β-cat⁻/⁻^ macrophages co-cultured with splenic CD4⁺ T-cells from OTII mice and stimulated with the OVA_323-339_ peptide, with or without XAV939 treatment. Data shown are the mean ± SEM (*n* = 4) with statistical significance assessed using one-way ANOVA. Statistical significance is indicated as ****p < 0.0001. **O** WT-*T. gondii* and its *T. gondii*^*ΔASP5*^ mutant were used to infect wild-type macrophages (Wt-MΦ). Following infection, the cells were harvested, and total cell lysates were analyzed by immunoblotting to detect AIM2, phospho-NLRP3, total NLRP3, phospho-β-catenin, β-catenin, phospho-IRF4, IRF4, phospho-PI3K, PI3K, phospho-AKT, AKT and β-actin (loading control). **P** TgESPs (observed as arrow head) represents numerous vesicles (~100–160 nm in diameter) with the morphological characteristics of exosomes, as observed under the transmission electron microscope (scale bar, 200 nm).

To determine which DNA (nuclear or mitochondrial) alone is sufficient to activate AIM2 and downstream NLRP3 mediated inflammasome activation, we purified mitochondrial DNA (mtDNA) and nuclear DNA (nDNA) from both uninfected and *T. gondii* infected (24 h.p.i.) MΦ^β-cat-FL/FL^ cells and compared their ability to trigger inflammasome activation. Previous studies indicate that cell-free mtDNA preferentially activates AIM2 over nDNA due to its prokaryotic features, such as unmethylated CpG motifs and circular structure, which are recognized by pattern recognition receptors (PRRs) like AIM2 [59]. This report aligns with our observation as we observed that AIM2 activation was absent in uninfected MΦ^β-cat-FL/FL^ macrophages; however, upon *T. gondii* infection, these cells exhibited AIM2 activation (lane 2, first panel, Fig. 5A) along with significant NLRP3 phosphorylation (lane 2, second panel, Fig. 5A).

In contrast, CD11b-MΦ^β-cat–/–^ macrophages showed significantly reduced activation of AIM2 (lane 4, first panel, Fig. 5A) or p-NLRP3 (lanes 4, second panel, Fig. 5A) after 24 h.p.i., likely due to their resistance to infection stemming from the absence of β-catenin, reduced ROS generation, and the inability to form oxidize mitochondrial DNA (Fig. 4G, S3D). As AIM2 is not easily detectable in normal cells due to low expression, thus we treated with the AIM2 agonist poly(dA-dT), we observed significant activation of AIM2 (lanes 5, first panel, Fig. 5A) and p-NLRP3 (lanes 5, second panel, Fig. 5A). mtDNA preferentially activates AIM2 over nDNA because it retains prokaryotic features, such as unmethylated CpG motifs and circular structure, which are recognized by PRRs like AIM2. Additionally, during stress or infection, mtDNA is often released in an oxidized form due to ROS generation, enhancing its immunostimulatory properties. In contrast, nuclear DNA, even when released, is less immunogenic due to its modifications (e.g., methylation) and association with histones, which dampen its recognition by PRRs like AIM2 [60]. To validate this, we purified mtDNA from uninfected MΦ^β-cat-FL/FL^ cells failed to activate AIM2 (lane 6, first panel, Fig. 5A) and NLRP3 (lanes 6, second panel, Fig. 5A) after transfection in uninfected Wt-MΦ macrophages, whereas mtDNA isolated from *T. gondii*-infected MΦ^β-cat-FL/FL^ cells (24 h.p.i.) activated both AIM2 (lane 7, first panel, Fig. 5A) and NLRP3 (lane 7, second panel, Fig. 5A) after transfection in uninfected Wt-MΦ, likely due to oxidative damage to mtDNA caused from elevated ROS (Fig. 4G). However, purified nDNA from both uninfected and infected MΦ^β-cat-FL/FL^ cells failed to activate AIM2 (lanes 8 and 9, first panel, Fig. 5A) or induce NLRP3 phosphorylation after transfection in uninfected Wt-MΦ macrophages (lanes 8 and 9, second panel, Fig. 5A). In conclusion, AIM2 and NLRP3 activation in MΦ^β-cat-FL/FL^ macrophages during *T. gondii* infection is driven by ROS-induced oxidation of mitochondrial DNA, while CD11b-MΦ^β-cat–/–^ cells remain resistant due to the absence of β-catenin and ROS generation.

In summary, β-catenin-driven ROS production can oxidize mtDNA which activates AIM2 and NLRP3 inflammasomes, driving immune responses during *T. gondii* infection. The absence of β-catenin impairs ROS production, preventing inflammasome activation and hindering parasite replication.

### Relative mtDNA copy number as an indicator of mitochondrial dysfunction

We previously demonstrated that mitophagy is initiated at an early stage of infection (6 h.p.i.; Fig. S3C), whereas prolonged infection results in mitophagy dysfunction, a hallmark of inflammation [50, 61]. This mitophagy impairment, or the associated mitochondrial dysfunction, leads to a reduction in mitochondrial DNA copy number [62], which serves as an indicator of mitochondrial loss and compromised mitochondrial homeostasis. qPCR analysis of *MT-ND1* normalized to the nuclear gene *HK2* revealed significant differences in relative mitochondrial DNA copy number (mtDNAcn) among MΦ^β-cat-FL/FL^ macrophages upon infection with *T. gondii* (Fig. S3F). Infected MΦ^β-cat-FL/FL^ macrophages exhibited a markedly lower mtDNAcn compared to uninfected MΦ^β-cat-FL/FL^ macrophages (*p* < 0.01). However, infected CD11b-MΦ^β-cat–/–^ macrophages did not show any mitochondrial dysfunction as their copy numbers were comparable to uninfected MΦ^β-cat-FL/FL^ or CD11b-MΦ^β-cat–/–^ macrophages. The pronounced reduction in mtDNAcn in infected MΦ^β-cat-FL/FL^ macrophages suggests enhanced mitophagy or mitochondrial degradation, likely reflecting underlying mitochondrial dysfunction. Since mitochondrial copy number is a marker of mitochondrial abundance and biogenesis, the observed depletion indicates impaired mitochondrial maintenance mechanisms in infected MΦ^β-cat-FL/FL^ macrophages.

### *T. gondii* infection induces NLRP3-casapse-1-IL1β inflammasome complex

Knowing that oxidized mtDNA in *T. gondii*-infected cells activates AIM2 and NLRP3 signaling, we aimed to investigate the role of β-catenin in inflammasome complex formation during infection. Specifically, we examined how β-catenin regulates inflammasome complex formation involving NLRP3, caspase-1, and ASC. Excessive ROS and mitochondrial dysfunction induce conformational changes in AIM2 and NLRP3, leading to their oligomerization and recruitment of apoptosis-associated speck-like protein containing a CARD (ASC). ASC, in turn, facilitates the recruitment and autocatalytic activation of caspase-1, which processes pro-IL-1β into its bioactive form by cleaving it at the aspartate residue [63]. Western blot analysis revealed that caspase-1 cleavage, producing both intermediate and mature forms, was observed only in MΦ^β-cat-FL/FL^ macrophages upon infection with *T. gondii* (first panel, Fig. 5B). In contrast, caspase-1 cleavage was absent in infected CD11b-MΦ^β-cat–/–^ macrophages (first panel, Fig. 5B). Similarly, cleavage of IL-1β, yielding both cleaved and mature IL-1β, occurred exclusively in MΦ^β-cat-FL/FL^ macrophages following *T. gondii* infection, whereas no IL-1β cleavage was detected in CD11b-MΦ^β-cat–/–^ macrophages under the same conditions (second panel, Fig. 5B). These findings indicate that β-catenin is crucial for inflammasome complex formation and the subsequent production of mature IL-1β during *T. gondii* infection. The effector protease caspase-1, of all inflammasomes complex is largely responsible to produce bioactive IL-1β through its cleavage of pro-IL-1β at the aspartate residue [64]. To determine whether IL-1β production during parasite infection is dependent on NLRP3, caspase-1, or ASC, we utilized wild-type RAW 264.7 macrophages alongside caspase-1^–/–^, ASC^–/–^, and NLRP3^–/–^ variants. These variant cells were infected for 12 h, and IL-1β processing into its mature form was assessed via immunoblotting and ELISA. Results showed that in wild-type RAW 264.7 macrophage, *T. gondii* infection induced the cleavage and production of mature IL-1β (Fig. 5C, G). However, RAW 264.7 macrophages deficient in either caspase-1 (Fig. 5D, G), or NLRP3 (Fig. 5E, G), or ASC (Fig. 5F, G) failed to produce IL-1β in response to *T. gondii* infection. The results indicate that IL-1β production during *T. gondii* infection is dependent on the presence of NLRP3, caspase-1, and ASC. Similarly, β-catenin-deficient macrophages infected with *T. gondii* also exhibited an absence of IL-1β production (Fig. 5B, G) further highlighting the essentiality of β-catenin in inflammasome-mediated IL-1β maturation. To investigate the involvement of NLRP3, caspase-1, and the ASC in β-catenin-dependent inflammasome complex formation, we used MΦ^β-cat-FL/FL^ and CD11b-MΦ^β-cat–/–^ macrophages infected with parasites. Immunoprecipitation with an NLRP3 antibody revealed that caspase-1 and ASC form a complex with NLRP3 in MΦ^β-cat-FL/FL^ cells. However, this complex formation was disrupted in CD11b-MΦ^β-cat–/–^ cells following infection (Fig. 5H). These findings underscore the pivotal role of β-catenin in orchestrating trimeric inflammasome complex formation during *T. gondii* infection. We summarize that this trimeric complex of NLRP3, caspase-1, and the ASC is essential for activating downstream IL-1β pathway. caspase-1 and IL-1β cleavage were observed only in β-catenin-expressing macrophages, highlighting β-catenin’s role in inflammasome-mediated IL-1β production. The absence of β-catenin impaired inflammasome complex formation and IL-1β production, confirming its crucial role in immune modulation. These findings elucidate the molecular pathways through which β-catenin influences inflammatory responses during *T. gondii* infection. Furthermore, transcriptomic analysis revealed heightened activation of the AIM2 mediated NLRP3 signalosome in parasite-infected mice, evidenced by elevated mRNA levels of *aim2* and *nlrp3* (Fig. S4I). XAV939 treatment (4 mg/kg body weight) significantly reduced the expression of *aim2*, *nlrp3*, and mitophagy-pyroptosis-related genes such as *pink1*, *gsdmd*, *casp1*, and *IL-1b* in both infected and uninfected mice (Fig. S4J–L). These results underscore the essential role of β-catenin in regulating ROCK2-IRF4-CYBB-ROS signaling (Fig. 4), driving mitophagy due to ROS-induced mt-DNA oxidation (Fig. 4) and activating the AIM2 and NLRP3-Caspase-1-IL-1β inflammasome complex (Fig. 5A–H). Additionally, β-catenin was identified as a fundamental regulator of gasdermin D-mediated pyroptosis in *T. gondii*-infected macrophages, with S552 phosphorylation playing a key role (Fig. 4J). In conclusion, β-catenin drives mitophagy and pyroptosis during *T. gondii* infection, while its absence preserves mitochondrial fitness, impeding parasite replication and mature PV formation.

### TLR11/12-independent AIM2 activation in macrophages

A long-standing question in the field has been how apicomplexan parasites like *T. gondii* activate innate PRRs in humans, given the absence of profilin-specific TLR11 and TLR12 [65], which are essential for parasite recognition in mice. To address this, we infected both murine RAW 264.7 where TLR11 was knockdown by its siRNA and human THP-1 macrophages with *T. gondii* and examined inflammasome activation (Fig. 5I). Since THP-1 cells naturally lack TLR11/12, and RAW 264.7 cells exhibit the observed upregulation of AIM2 and phosphorylation of NLRP3 in response to infection regardless of the presence or absence of TLR11, these findings suggest activation through canonical TLR11/12-independent sensing pathways consistent with observations in murine models even when TLR11 is present (Fig. 4L). Importantly, the reduction in NLRP3 phosphorylation upon AIM2 knockdown (Fig. 4M) suggests that AIM2 functions upstream and is critical for full inflammasome activation, independent of TLR11/12 signaling. These findings point toward a host-intrinsic sensing mechanism in which oxidized mtDNA (Fig. S3D), released during infection-induced mitochondrial stress, serves as a host-associated molecular pattern (HAMP) that directly activates AIM2 and initiates downstream inflammatory responses (Fig. 5A). Supporting this, our earlier data demonstrate a strong link between β-catenin signaling and this pathway. Activation of β-catenin (Fig. 5I) leads to the generation of ROS (Fig. 4G), which in turn oxidize mtDNA. This oxidized mtDNA is then sensed by AIM2 (Fig. 5A), triggering inflammasome assembly (Fig. 5H) and subsequent NLRP3 activation. Together, these results reveal a novel innate sensing axis involving mtDNA, AIM2, and β-catenin that enables human macrophages to detect *T. gondii* in the absence of TLR11/12. To further validate this model, we monitored inflammasome activation in both RAW 264.7 and THP-1 cells following *T. gondii* infection. In THP-1 macrophages, we observed a clear time-dependent increase in the expression of key signaling components, including phospho-β-catenin, AIM2, and phospho-NLRP3, as shown by immunoblotting (Figs. 4I, 5I). This immune activation was accompanied by an increase in parasite burden, as indicated by enhanced SAG1 expression (Fig. 5I).

Together, these results demonstrate that *T. gondii* activates the AIM2 which facilitates NLRP3 inflammasome axis in both mice and human through a TLR11/12-independent mechanism. Mitochondrial dysfunction and ROS-driven release of oxidized mtDNA act as endogenous danger signals that trigger β-catenin-dependent AIM2 activation and followed by inflammatory responses. This work uncovers a previously unrecognized innate immune surveillance mechanism in humans, offering new insight into host innate immune recognition against *T. gondii* infection.

### β-catenin of macrophages enables IL-1β and IFN-γ secretion in parasite antigen response

The IL-1 family of cytokines are tightly regulated because of their highly inflammatory potential, excessive production of these cytokines is linked to many inflammatory and autoimmune disorders [66]. We aim to define how β-catenin in macrophages regulates antigen presentation through MHC to T-cells, and the role it plays in driving IL-1β production and inflammation in response to parasite antigens. When MΦ^β-cat-FL/FL^ macrophages were incubated with complete soluble antigens (CSA) derived from parasites for 12 h, we observed significant IL-1β secretion from macrophages (second bar, Fig. 5J). However, CD11b-MΦ^β-cat–/–^ macrophages lacking β-catenin were unable to induce substantial IL-1β production in response to CSA (fourth bar, Fig. 5J). To demonstrate that IL-1β activation is independent of TLR11/12, we assessed IL-1β release in THP-1 macrophages. We observed that *T. gondii* infection at 12 h induces IL-1β secretion (Fig. 5K), consistent with our findings in the mouse model. This suggests that the entry of live parasites triggers ROS production, leading to the release of oxidized mitochondrial DNA, which in turn activates AIM2 and inflammasome complex-mediated IL-1β release, an innate immune response that occurs independently of TLR11/12 signaling.

Similarly, MΦ^β-cat-FL/FL^ macrophages when incubated with IL-12 and IL-18 could release IFN-γ (second bar, Fig. 5L). When MΦ^β-cat-FL/FL^ macrophages were infected with parasites for 12 h, we observed significant IFN-γ secretion from macrophages (third bar, Fig. 5L). However, CD11b-MΦ^β-cat–/–^ macrophages lacking β-catenin were unable to induce substantial IFN-γ production in response to parasites infection (fifth bar, Fig. 5L).

### β-catenin dependent cognate interaction of macrophages with T-cells enables IFN-γ secretion

Cognate interaction refers to the direct antigen-specific interaction between macrophages (MHC) and T-cells (TCRs). To test this, we incubated MΦ^β-cat-FL/FL^ macrophages with complete soluble antigens (CSA) derived from parasites for 12 h, we observed similar significant IFN-γ secretion from macrophages (sixth bar, Fig. 5L). However, CD11b-MΦ^β-cat–/–^ macrophages lacking β-catenin were unable to induce substantial IFN-γ production in response to CSA (eighth bar, 5 L). Here, we would like to demonstrate that β-catenin in macrophages enhances their ability to present *T. gondii* CSA via MHC to antigen-primed T-cells, collected from spleen of infected mice, thereby facilitating IFN-γ secretion and promoting inflammation (eleventh bar, Fig. 5L), whereas unprimed T-cells from uninfected mice cannot induce to release IFN-γ (tenth bar, Fig. 5L). Furthermore, γ-irradiated MΦ^β-cat-FL/FL^ macrophages treated with CSA failed to secrete IFN-γ after 12 h of incubation due to macrophage inactivation (ninth bar, Fig. 5L). Interestingly, when these γ-irradiated MΦ^β-cat-FL/FL^ macrophages with CSA were co-cultured with purified splenic CD3^+^ T-cells isolated from β-catenin^flox^ mice at 10 days post-infection, enhanced IFN-γ production from those CD3^+^ T-cells was observed (eleventh bar, Fig. 5L). This enhanced secretion of IFN-γ from T-cells in the co-culture set up is attributed through antigen presentation via MHC and subsequent recognition by T-cells, as these T-cells were derived from infected β-catenin^flox^ mice. Conversely, T-cells isolated from β-cat^ΔMΦ^-infected mice, when incubated with γ-irradiated CD11b-MΦ^β-cat–/–^ macrophages treated with CSA, did not elicit IFN-γ secretion (thirteenth bar, Fig. 5L). This lack of IFN-γ production from T-cells from β-cat^ΔMΦ^-infected mice is likely due to the absence of β-catenin in macrophages, which rendered the β-cat^ΔMΦ^ mice resistant to infection, thereby preventing IFN-γ-mediated inflammation in their T-cells. It is known that T-cells undergo a stepwise process of early activation, expansion, and differentiation into effector cells through MHC-TCR interactions [64]. We also found that macrophages from IFN-γ^–/–^ mice were unable to release IFN-γ in presence of CSA (seventeenth bar, Fig. 5L). Here, we demonstrate that the “signal-three” hypothesis of antigen presentation is regulated by macrophage-intrinsic β-catenin signaling. Our findings indicate that in the presence of β-catenin, antigens-exposed macrophages are capable of effectively presenting antigens via MHC-TCR interactions to T cells, thereby promoting IFN-γ production and a pro-inflammatory response. In contrast, the absence of β-catenin disrupts this “signal-three” hypothesis, impairing IL-1β-mediated inflammatory signaling (Fig. 5J) and abrogating IFN-γ production from both macrophages and T cells (Fig. 5L). We conclude that β-catenin enables macrophages to efficiently present parasite antigens via MHC to T-cells, thereby promoting IL-1β and IFN-γ production and inflammation. In contrast, the absence of β-catenin impairs antigen presentation and inhibits IFN-γ-driven responses in T-cells. To determine whether IFN-γ release is independent of TLR11/12 signaling, we compared infected and uninfected THP-1 cells. The enhanced IFN-γ production observed in infected THP-1 cells suggests that parasite-induced IFN-γ secretion occurs independently of TLR11/12 (Fig. 5M).

To evaluate whether this effect extends beyond *T. gondii* antigens or to confirm that the cognate interaction is globally dependent to β-catenin, we examined IL-1β production in response to ovalbumin (OVA) antigen presentation [64]. For this, we used a well-defined cognate peptide, OVA₃₂₃-₃₃₉. We examined its presentation by MHCII of either MΦ^β-cat-FL/FL^ or CD11b-MΦ^β-cat–/–^ macrophages to OVA peptide-restricted TCR transgenic effector splenic CD4⁺ T-cells from OT-II mice. Notably, in co-cultures of OVA peptide-restricted OT-II TCR transgenic effector CD4^+^ T-cells with MΦ^β-cat-FL/FL^ macrophages, IL-1β production was observed only in the presence of the cognate OVA_323-339_ peptide (25 µM, third bar, Fig. 5N). In contrast, IL-1β secretion was significantly reduced in CD11b-MΦ^β-cat–/–^ macrophages co-cultured with OT-II TCR transgenic effector CD4^+^ T-cells and the OVA_323-339_ peptide (fifth bar, Fig. 5N), indicating that β-catenin in macrophages is essential for IL-1β production. Moreover, when MΦ^β-cat-FL/FL^ macrophages were co-cultured with OT-II TCR transgenic effector CD4^+^ T cells in the presence of OVA_323-339_ and the β-catenin inhibitor XAV939, IL-1β secretion was abrogated (sixth bar, Fig. 5N). These findings demonstrate that the β-catenin-dependent enhancement of MHC-TCR interaction avidity is a critical factor determining the level of IL-1β secretion demonstrating its broader immunomodulatory impact beyond *T. gondii* infection which may influence inflammatory immune responses.

### *T. gondii*’s aspartyl protease 5 (ASP5)-dependent parasite secretion is required for activation of the PI3K-AKT-β-catenin-IRF4-AIM2-NLRP3 axis in macrophages

After discovering that PI3K-AKT-β-catenin-IRF4-ROS mediated inflammasome activation occurs in macrophages during *T. gondii* infection, we sought to identify which *T. gondii* protein may contribute to trigger this response. *T. gondii* excretory/secretory proteins (TgESPs) are a group of proteins secreted by the parasite. We reasoned that identifying *T. gondii* TgESPs that activate the host β-catenin pathway could provide insights into β-catenin-NLRP3 axis activation. ASP5 is a golgi-resident, key component of the exported protein 2 (EXP2)-dependent secretion pathway in *T. gondii*. It plays a very critical role in parasite’s fitness and mutation in ASP5 impairs proteolytic processing, exhibits defective secretion leading to reduced parasite fitness and virulence [17]. Hence to address the role of ASP5-dependent secretory pathway in β-catenin signaling, we used *T. gondii* (wild type) and its ASP5 mutant, *T. gondii*^ΔASP5^ and infected in macrophages. Our findings revealed that ASP5 is required for PI3K-AKT-β-catenin-IRF4-AIM2 activation and NLRP3 signalosome formation (Fig. 5O). Specifically, *T. gondii*^ΔASP5^ mutant parasites failed to induce of PI3K-AKT-β-catenin-IRF4-AIM2-NLRP3 activation. These results suggest that ASP5-dependent processes are required for inflammasome actiavtion in macrophages.

We observed that TgESPs, extracted through centrifugation, also contained numerous vesicles (~100–160 nm in diameter) exhibiting the morphological characteristics of exosomes, as seen under transmission electron microscopy (Fig. 5P). Notably, the release of TgESPs was significantly impaired in *T. gondii*^ΔASP5^ mutant parasites. These data suggest that ASP5-dependent secretion is required for delivery of parasite-derived factors that activate host β-catenin signaling.

### Role of β-catenin in M1-M2 macrophage polarization in *T. gondii* infection

**Fig. 6:**
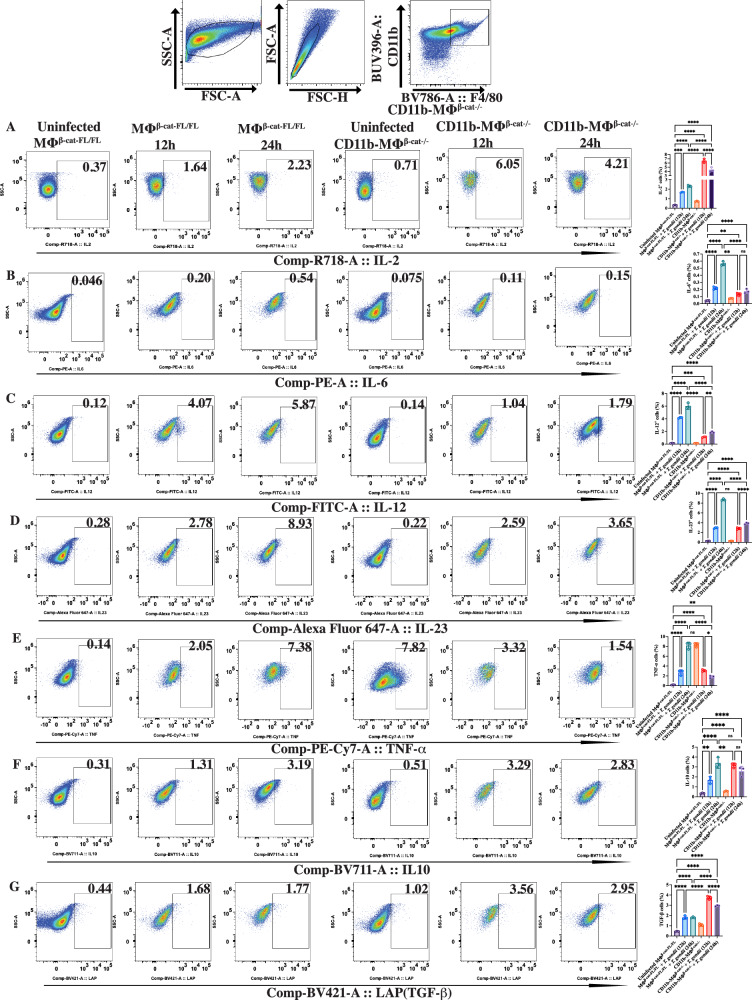
β-catenin regulates M1-M2 macrophage polarization following parasitic infection. **A–G** MΦ^β-cat-FL/FL^ and CD11b-MΦ^β-cat⁻/⁻^ macrophages were infected with parasites, and the expression of intracellular cytokines such as IL-2, IL-6, IL-12, IL-23, TNF-α, IL-10, and LAP (TGF-β) was assessed by flow cytometry at 12 and 24 h.p.i. The flow bar plots for each cytokine represent data from three independent experiments. Data shown are the mean ± SEM (*n* = 3) with statistical significance assessed using one-way ANOVA. Statistical significance is indicated as ****p < 0.0001, ***p < 0.001, **p < 0.01, *p < 0.05, and “ns” indicating no significance.

Our findings revealed that β-catenin orchestrates immune responses during *T. gondii* infection driving PI3K-AKT pathway activation and facilitates parasite replication (Fig. 1). It drives inflammasome activation through IRF4 transcription, CYBB activation, and ROS generation. Enhanced ROS oxidized mtDNA to activate AIM2 mediated NLRP3 signaling. Meanwhile, *T. gondii*’s ASP5 facilitates β-catenin-IRF4-AIM2 mediated NLRP3 activation and IL-1β production, which supports immune modulation and promotes parasite survival (Figs. 3–5). We hypothesize that NLRP3-ASC-caspase-1-IL-1β inflammasome complex plays a crucial role in fueling M1 macrophage polarization particularly during infections. In contrast, the absence of β-catenin inhibits the formation of inflammasome complexes while preserving mitochondrial integrity (Fig. 4K) which regulates M2 polarization (Fig. 6). To address our hypothesis, BMDMs were differentiated from β-catenin^flox^ and β-cat^ΔMΦ^ mice, and CD11b^+^F4/80^+^ double-positive cells were gated for cytokine analysis. IL-2, a pivotal cytokine supporting an anti-inflammasome environment by promoting CD4^+^ T-cell differentiation into the Treg lineage [67], was significantly reduced in infected MΦ^β-cat-FL/FL^ macrophages compared to infected CD11b-MΦ^β-cat–/–^ macrophages (Fig. 6A). Conversely, IL-6, a critical mediator of chronic inflammation in inflammatory diseases [68], was released in significantly higher amounts by infected MΦ^β-cat-FL/FL^ macrophages over time compared to infected CD11b-MΦ^β-cat–/–^ macrophages (Fig. 6B). The inflammasome activation in infected MΦ^β-cat-FL/FL^ macrophages amplified the production of pro-inflammatory cytokines, reinforcing the M1 phenotype and driving chronic inflammation. Cytokines like IL-12 and IL-23, known drivers of chronic inflammation [69], were also secreted at significantly higher levels by infected MΦ^β-cat-FL/FL^ macrophages in a time-dependent manner compared to infected CD11b-MΦ^β-cat–/–^ macrophages, indicating their dependence on β-catenin activation (Fig. 6C, D). While TNF-α is predominantly of myeloid origin during PRR-driven inflammation [64], our study demonstrates that mt-DNA-AIM2 activation (Fig. 5A) enhances TNF-α levels via β-catenin-dependent inflammasome activation (Fig. 5E). Ablating β-catenin not only restricted inflammasome activity but also impeded TNF-α secretion (Fig. 6E), limiting parasite growth (Fig. 2L). Furthermore, TNF-α and IL-10 are known to exhibit duelling duo roles [70]. Consistently, we observed elevated IL-10 secretion in infected CD11b-MΦ^β-cat–/–^ macrophages compared to MΦ^β-cat-FL/FL^ macrophages at both 12 and 24 h.p.i. (Fig. 6F). Treg cells require arming of not only IL-2, IL-10, but also TGF-β from monocytes for their sustained anti-inflammatory function [71]. Macrophages emerged as a major source of these cytokines, and infected CD11b-MΦ^β-cat–/–^ macrophages exhibited higher TGF-β (LAP) levels than MΦ^β-cat-FL/FL^ macrophages (Fig. 6G). These findings demonstrate that β-catenin plays a crucial role in regulating macrophage polarization, shifting them toward a pro-inflammatory M1 state that supports parasite growth. In contrast, the absence of β-catenin enhances the production of IL-2, IL-10, and TGF-β, suppresses inflammasome activity, and preserves mitochondrial fitness (Fig. 4K), thereby creating an anti-inflammatory environment. This M2-like state, characterized by reduced inflammasome activation and limited pro-inflammatory mediator release, effectively mitigates the infection (Fig. 2), and restricts parasite replication.

At 12 h post *T. gondii* infection, macrophages displayed distinct polarization patterns depending on β-catenin status. qRT-PCR analysis revealed that infected MΦ^β-cat-FL/FL^ macrophages significantly upregulated Glut1 and iNOS, canonical M1 markers, relative to uninfected controls (Fig. S5E). Flow cytometry confirmed a parallel increase in the surface marker CD80 (Fig. S5E, F). In contrast, CD11b-MΦ^β-cat–/–^ macrophages showed markedly reduced expression of Glut1, iNOS, and CD80 following infection, indicating that β-catenin is required for optimal M1 polarization. Conversely, markers of M2 polarization were regulated in the opposite direction. Infected MΦ^β-cat-FL/FL^ macrophages exhibited strong downregulation of arginase and CHI3L3 (Ym1), together with a reduced frequency of CD206⁺ cells (Fig. S5E, F). By contrast, CD11b-MΦ^β-cat–/–^ macrophages displayed a significant induction of arginase and CHI3L3, along with higher CD206 expression, compared with infected controls. Collectively, these results demonstrate that β-catenin signaling drives M1 polarization during early *T. gondii* infection, while its absence favors an M2-like program, suggesting a protective and tissue-repairing role of β-catenin deficiency against excessive pro-inflammatory activation.

### β-catenin-dependent metabolic reprogramming governs M1 and M2 macrophage responses during *T. gondii* infection

**Fig. 7:**
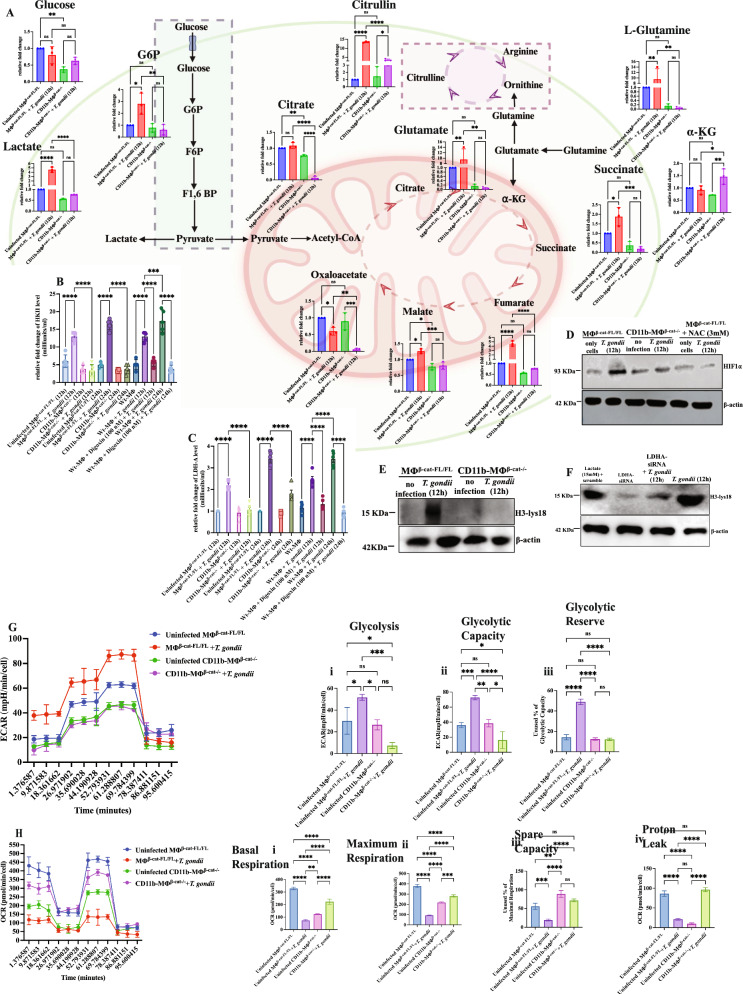
β-catenin regulates the metabolic profile of macrophages, influencing their polarization in response to parasitic infection. **A** Macrophages, MΦ^β-cat-FL/FL^ and CD11b-MΦ^β-cat⁻/⁻^, were infected with parasites for 12 h, and the relative fold changes of key glycolysis and TCA cycle metabolites were measured. The bar diagrams represent the average data from three experiments conducted within the same batch for each metabolite. Data shown are the mean ± SEM (*n* = 3) with statistical significance assessed using one-way ANOVA. Statistical significance is indicated as ****p < 0.0001, ***p < 0.001, **p < 0.01, *p < 0.05, and “ns” indicating no significance. Abbreviations: G6P, glucose-6-phosphate; F6P, fructose-6-phosphate; α-KG, α-ketoglutarate. Relative **B** hexokinase II (HKII) activity and **C** lactate dehydrogenase-A (LDH-A) activity in macrophages (MΦ^β-cat-FL/FL^ and CD11b-MΦ^β-cat⁻/⁻^) were measured following parasite infection at different time points. To evaluate the role of HIF1α in regulating hexokinase stability, macrophages were infected in the presence of the HIF1α inhibitor digoxin, and the levels of HKII and LDH-A were measured. The data are presented as mean ± SEM of biological replicates (*n* = 5), with statistical significance evaluated using one-way ANOVA (non-parametric or mixed). Statistical significance is indicated as ****p < 0.0001 and ***p < 0.001. Macrophages, MΦ^β-cat-FL/FL^ and CD11b-MΦ^β-cat⁻/⁻^, were infected with parasites and cells were harvested and total cellular lysates were subjected to immunoblotting to detect **D** HIF1α, and **E, F** lactylation of histone using specific antibodies, with β-actin serving as the loading control. NAC, ROS scavenger was used in uninfected and infected MΦ^β-cat-FL/FL^ macrophages and HIF1α expression was checked using its antibody. Lactate was used as positive control, and siRNA was used to impede the expression of LDH-A **G** ECAR profiles and glycolytic parameters of macrophages following infection were measured. Non-glycolytic acidification was recorded first, after which glucose was added to the cells, followed by oligomycin to inhibit ATP synthase, thereby inducing maximal glycolysis to compensate for ATP loss due to mitochondrial inhibition. Finally, 2-deoxyglucose (2-DG) was added to block glycolysis, confirming that the observed acidification was a result of glycolytic activity. The profiles and glycolytic parameters along with (i) glycolysis, (ii) glycolytic capacity and (iii) glycolytic reserve are presented with bar diagram and data are presented as mean ± SEM (*n* = 3), with statistical significance assessed using one-way ANOVA. Statistical significance is indicated as ****p < 0.0001, ***p < 0.001, **p < 0.01, *p < 0.05, and “ns” representing no significance. **H** Respiratory profiles (OCR) and (i-iv) respiratory parameters of macrophages were measured, after obtaining basal respiration, cells were subjected to oligomycin, which inhibits ATP synthase and demonstrates the mitochondrial ATP-linked OCR, followed by FCCP (cyanide-4-[trifluoromethoxy]phenylhydrazone), which uncouples mitochondrial respiration and maximizes OCR, and finally antimycin A and rotenone (AntiA and Rot), which inhibit complex III and I in the ETC, respectively, and shut down respiration. Profiles and respiratory parameters are representative of three independent experiments. Data shown are the mean ± SEM (*n* = 3) with statistical significance assessed using one-way ANOVA. Statistical significance is indicated as ****p < 0.0001, ***p < 0.001, **p < 0.01, *p < 0.05, and “ns” representing no significance.

In line with the above studies, our next aim was to investigate how β-catenin regulates metabolic reprogramming in immune cells by LC-MS/MS technique. Specifically, we examined its role in shaping M1 and M2 macrophage responses during *T. gondii* infection and how it influences immune modulation to determine parasite fate. M1 macrophages in an infected condition predominantly utilize aerobic glycolysis (Warburg metabolism) [72, 73], characterized by reduced TCA cycle activity and succinate accumulation, which drive pro-inflammatory responses. In contrast, M2 macrophages rely on lower glycolytic flux and enhanced oxidative phosphorylation, supported by fatty acid oxidation, to facilitate anti-inflammatory functions and tissue repair [74]. Here, we demonstrate that the glycolytic switch in infected M1 macrophages is downregulated in M2 macrophages in a β-catenin-dependent manner. To demonstrate this, bone-marrow differentiated CD11b^+^F4/80^+^ double-positive cells were sorted, isolated from β-catenin^flox^ and β-cat^ΔMΦ^ mice and infected with parasites for 12 h ex vivo, and samples were prepared for LC-MS/MS analysis. Mass spectrometry data revealed that glucose uptake varied between uninfected MΦ^β-cat-FL/FL^ and CD11b-MΦ^β-cat–/–^ macrophages; however, during infection, glucose uptake was comparable (Fig. 7A). Notably, glucose-6-phosphate levels were elevated in infected MΦ^β-cat-FL/FL^ macrophages compared to CD11b-MΦ^β-cat–/–^ (Fig. 7A). Consistent with the Warburg effect in inflammatory cells [72], infected MΦ^β-cat-FL/FL^ macrophages showed high lactate production, whereas CD11b-MΦ^β-cat–/–^ macrophages exhibited significantly reduced glucose-to-lactate catabolism (Fig. 7A), indicating an enhanced Warburg effect in M1 macrophages post-*T. gondii* infection. M1 macrophages demonstrated increased ROS (Fig. 4G), citrate efflux (Fig. 7A), and stabilized succinate production (Fig. 7A), which activated HIF1α (Fig. 7D) [74, 75]. Elevated citrate and citrulline levels were observed, essential for pro-inflammatory responses and M1 polarization, respectively (Fig. 7A) [76, 77]. Our data confirmed heightened citrate and succinate conversion in infected MΦ^β-cat-FL/FL^ macrophages (Fig. 7A), which are M1 in nature as they release a spectrum of inflammatory cytokines (Fig. 6B–E). Additionally, a metabolic break was identified at the isocitrate-to-α-ketoglutarate conversion, explaining TCA cycle fragmentation in M1 macrophages (Saroj P. Mathupala et al., 1995; Mills et al., 2016). Succinate and malate conversions were elevated, while α-ketoglutarate, fumarate, and oxaloacetate levels were reduced in infected MΦ^β-cat-FL/FL^ macrophages, stating TCA mediated metabolic break in infected MΦ^β-cat-FL/FL^ macrophages which is a signature marker of M1 polarization [78]. In contrast, M2 macrophages, which rely on oxidative phosphorylation, exhibited stable levels of TCA cycle intermediates such as citrate, malate, succinate, and oxaloacetate without any significant fluctuations, promoting anti-inflammatory and reparative functions [74, 79]. This metabolic profile enhances mitochondrial respiration, meeting the energy demands of tissue repair and immune regulation. Similarly, our data indicated that infected CD11b-MΦ^β-cat–/–^ macrophages are M2 in nature and they release battery of regulatory cytokines (Fig. 6A, F, G) due to stable catabolism of TCA cycle intermediates, including citrate, malate, succinate, and oxaloacetate (Fig. 7A), supporting an anti-inflammatory environment to mitigate infection (Fig. 2). In summary, β-catenin regulates glucose metabolism in infected macrophages, driving a pro-inflammatory M1 phenotype through Warburg metabolism and TCA cycle disruption to supports parasites growth. While absence of β-catenin promotes an anti-inflammatory M2 state via OXPHOS. This metabolic shift in M2 macrophages shapes regulatory immune responses for mitigating pathogen burden.

### β-catenin-driven ROS-HIF-1α-HKII-LDH-A axis mediates metabolic reprogramming, Warburg effect, and histone lactylation in M1 polarization during parasite infection

To decipher the β-catenin-driven metabolic reprogramming pathways underlying macrophage polarization, we examined the key metabolic and epigenetic regulators involved during parasite infection. Our findings indicate that β-catenin regulates macrophage metabolism, driving M1 polarization through Warburg metabolism and TCA cycle disruption (Fig. 7A). Hexokinase II (HKII) is central to the Warburg effect, enhancing glycolysis and lactate production [31, 80]. Hence, to investigate the role of the Warburg effect in glucose metabolism during M1 polarization, we measured HKII enzymatic activity. Infected MΦ^β-cat-FL/FL^ macrophages showed significantly higher HKII activity at both 12 h.p.i. and 24 h.p.i. (second and sixth bars, Fig. 7B), whereas infected CD11b-MΦ^β-cat–/–^ macrophages, characterized by a stable TCA cycle (Fig. 7A), exhibited significantly reduced HKII activity (fourth and eighth bars, Fig. 7B).

Based on the results presented above, we demonstrated that elevated ROS levels (Fig. 4G) contribute to mitochondrial disintegration (Fig. 4K) during infection. Previous studies have reported that increased ROS also plays a crucial role in stabilizing hypoxia-inducible factor 1-α (HIF-1α) in cells [81]. We hypothesize here that ROS-mediated HIF-1α can regulate HKII activity. To further investigate this, we infected MΦ^β-cat-FL/FL^ macrophages with *T. gondii* for 12 h and observed a significant upregulation of HIF-1α expression, as confirmed by immunoblot analysis (lane 2, Fig. 7D). However, in CD11b-MΦ^β-cat–/–^ macrophages, where ROS is low due to ablation of β-catenin, *T. gondii* infection failed to induce HIF-1α expression (lane 4, Fig. 7D), suggesting that β-catenin reliant ROS is essential for HIF-1α upregulation. To assess whether ROS directly contribute to HIF-1α stabilization, we treated MΦ^β-cat-FL/FL^ macrophages with NAC, and infecting them with *T. gondii*. NAC treatment resulted in a further reduction of HIF-1α expression even in presence of β-catenin after infection (lane 6, Fig. 7D), confirming that *T. gondii* infection enhances ROS levels, which in turn stabilize HIF-1α to drive downstream signaling. Previous studies demonstrated that HIF-1α stabilization upregulates HKII, driving glycolysis and metabolic reprogramming [29]. To confirm that HKII activity is regulated by HIF-1α, we treated infected CD11b^+^F4/80^+^ Wt-MΦ cells with digoxin, a specific inhibitor of HIF-1α, and assessed HKII activity. Inhibition of HIF-1α with digoxin significantly suppressed HKII activity in infected CD11b^+^F4/80^+^ Wt-MΦ (ninth to thirteenth bars, Fig. 7B), confirming the essential role of HIF-1α in HKII stabilization. Another key enzyme that reinforces the Warburg effect is lactate dehydrogenase A (LDH-A) which drives the conversion of glucose-derived pyruvate into lactate, a key step in maintaining glycolysis under aerobic conditions [73, 82]. We found infected MΦ^β-cat-FL/FL^ macrophages showed significantly higher LDH-A activity at both 12 h.p.i. and 24 h.p.i. (second and sixth bars, Fig. 7C), whereas infected CD11b-MΦ^β-cat–/–^ macrophages after 12 hp.i. and 24 h.p.i exhibited significantly reduced LDH-A activity (fourth and eighth bars, Fig. 7C), highlight essential role of β-catenin in Warburg effect. Inhibition of HIF-1α with digoxin significantly reduced LDH-A activity (ninth to thirteenth bars, Fig. 7C) by suppressing HKII activity (Fig. 7B) in infected CD11b^+^F4/80^+^ Wt-MΦ, highlighting the essential role of the HIF-1α-HKII-LDH-A metabolic axis in driving the Warburg effect and M1 polarization. Beyond metabolism, lactate also functions as an epigenetic regulator. When intracellular lactate levels rise, histone lactylation sustains the transcription of inflammatory and glycolytic genes, reinforcing the M1 phenotype during infection [83]. Elevated lactate levels with higher LDH-A activity in infected MΦ^β-cat-FL/FL^ (Fig. 7A, C), a byproduct of glycolysis, drive histone lactylation in presence of parasite infection which is absent in infected CD11b-MΦ^β-cat–/–^ macrophages (Fig. 7E), a novel post-translational modification linking metabolism to gene regulation. When LDH-A was knocked down using siRNA, histone lactylation was notably suppressed during *T. gondii* infection (lane 3, Fig. 7F), indicating a critical role for LDH-A-derived lactate in this modification. In contrast, exogenous lactate treatment of Wt-MΦ in the absence of infection was sufficient to induce histone lactylation (lane 1, Fig. 7F), confirming that lactate itself is a key driver of this epigenetic mark. Furthermore, removal of siRNA restored LDH-A expression in infected Wt-MΦ, allowing histone lactylation to occur at 12 h post-infection (lane 4, Fig. 7F). These findings collectively demonstrate that LDH-A-dependent lactate production is essential for *T. gondii*-induced histone lactylation in macrophages, underscoring the link between host metabolic reprogramming and parasite-driven epigenetic regulation.

qRT-PCR analysis revealed that infected MΦ^β-cat-FL/FL^ macrophages significantly upregulated Glut1 and iNOS, canonical M1 markers, relative to uninfected controls (Fig. S5E). Flow cytometry confirmed a parallel increase in the surface marker CD80 (Fig. S5E, F). In contrast, CD11b-MΦ^β-cat–/–^ macrophages showed markedly reduced expression of Glut1, iNOS, and CD80 following infection, indicating that β-catenin is required for optimal M1 polarization. Conversely, markers of M2 polarization were regulated in the opposite direction. Infected MΦ^β-cat-FL/FL^ macrophages exhibited strong downregulation of arginase and CHI3L3 (Ym1), together with a reduced frequency of CD206⁺ cells (Fig. S5E, F). By contrast, CD11b-MΦ^β-cat–/–^ macrophages displayed a significant induction of arginase and CHI3L3, along with higher CD206 expression, compared with infected controls. Collectively, these results demonstrate that β-catenin signaling drives M1 polarization during early *T. gondii* infection, while its absence favors an M2-like program, suggesting a protective and tissue-repairing role of β-catenin deficiency against excessive pro-inflammatory activation.

To further determine whether lactate is essential for M1 macrophage polarization, we inhibited lactate production by silencing LDH-A using siRNA. qRT-PCR analysis showed that Wt-MΦ macrophages displayed a robust increase in Glut1, iNOS and IL-1β expression at 12 h.p.i. following *T. gondii* infection (Fig. S3G–I). However, LDH-A silencing markedly blunted the expression of both Glut1, iNOS and IL-1β in wild-type macrophages at the same time point. Together, these results indicate that lactate production via LDH-A is critical for sustaining the glycolytic program (Glut1 and iNOS) and pro-inflammatory cytokine output (IL-1β), thereby supporting M1 macrophage polarization during infection (Fig. S3G–I). Although Glut1 functions upstream of LDHA in the glycolytic pathway, its expression during M1 polarization is strongly influenced by LDHA activity. When LDHA is inhibited, glycolysis slows due to a lack of NAD⁺ regeneration, leading to reduced lactate-HIF-1α signaling and a compensatory drop in both Glut1 and iNOS expression.

Transcriptomic analysis revealed heightened activation of glycolysis and the Warburg effect, along with disruption of the TCA cycle, in splenocytes of parasite-infected mice. This was characterized by increased expression of several glycolysis-related genes like *Pkm* (pyruvate kinase M), *Tpi1*, (triosephosphate isomerase 1), *Pgk1* (phosphoglycerate kinase 1), *Pgam1* (phosphoglycerate mutase 1), *Pdha1* (pyruvate dehydrogenase-A1), *Ldha* (lactate dehydrogenase-A), *Hk2*, (hexokinase 2), *Hif1a* (hypoxia-inducible factor-1), *Gapdg* (glyceraldehyde-3-phosphate dehydrogenase) *Eno2* (enolase 2), and *Aldoa*, (fructose-bisphosphate aldolase A) (Fig. S4M). However, we found suppression of transcription of multiple TCA cycle-related genes like *Sdhb* (succinate dehydrogenase B), *Sdha* (succinate dehydrogenase A), *Ogdh* (2-oxoglutarate dehydrogenase), *Mdh1* (malate dehydrogenase 1), *Idh3g* (isocitrate dehydrogenase), and *Idh1/2* (NADP(+)-dependent isocitrate dehydrogenase-1/2), as evidenced by encumbered mRNA levels (Fig. S4M). To support the transcriptomic data (Fig. S4M), LC-MS/MS analysis revealed lower expression levels of oxaloacetate, fumarate, and α-ketoglutarate in parasite-infected macrophages (Fig. 7A). Meanwhile, several TCA cycle-related genes, such as Idh3a (isocitrate dehydrogenase (NAD( + )) 3 catalytic subunit alpha), Dlst (dihydrolipoamide S-succinyltransferase), and Dld (dihydrolipoamide dehydrogenase), exhibited elevated mRNA expression (Fig. S4M). This was further supported by LC-MS/MS data, which showed increased levels of citrate, succinate, and malate in macrophages infected with *T. gondii* (Fig. 7A). The transcriptomic analysis (Fig. S4M), along with metabolomics data (Fig. 7A), indicates a disruption of the TCA cycle infi*T.* *gondii* infection. Treatment with XAV939 (4 mg/kg body weight) significantly downregulated the expression of glycolysis-related genes in both infected and uninfected mice (Fig. S4N–P) which also supports mass-spectrometry data where CD11b-MΦ^β-cat–/–^ macrophages showed lower expression of several metabolites (glucose-6-phosphate and lactate) related to glycolysis (Fig. 7A). In summary, infected MΦ^β-cat-FL/FL^ macrophages exhibit elevated ROS-HIF-1α-driven HKII and LDH-A activity, promoting the Warburg effect, lactate production, and histone lactylation, which are absent in infected CD11b-MΦ^β-cat–/–^ macrophages. These findings underscore the pivotal role of β-catenin in regulating the HIF-1α-HKII-LDH-A metabolic axis, which drives metabolic reprogramming and M1 polarization during parasite infection.

### β-catenin modulates glycolytic and mitochondrial pathways to influence macrophage immunometabolism landscape

We then assessed glycolytic (ECAR) and mitochondrial (OCR) function to elucidate the role of β-catenin in regulating redox balance during 12 h *T. gondii* infection. Using a Seahorse XF extracellular flux analyzer (Agilent), we analyzed the metabolic profiles of MΦ^β-cat-FL/FL^ and CD11b-MΦ^β-cat–/–^ macrophages under infection conditions. Enhanced glycolysis, characteristic of the Warburg effect, promotes pro-inflammatory responses in M1 macrophages by driving the efflux of TCA intermediates such as citrate and succinate (Fig. 7A), which support basal extracellular acidification rates (ECAR) and proinflammatory cytokine production [84]. Our findings indicate that β-catenin plays a critical role in regulating glycolysis. Uninfected MΦ^β-cat-FL/FL^ macrophages exhibited a higher basal ECAR compared to uninfected CD11b-MΦ^β-cat–/–^ macrophages (Fig. 7G), consistent with increased glucose uptake (Fig. 7A). Upon infection, ECAR was further elevated in MΦ^β-cat-FL/FL^ macrophages compared to infected CD11b-MΦ^β-cat–/–^ macrophages (Fig. 7G). Additionally, glycolysis, glycolytic capacity, and glycolytic reserve were significantly higher in infected MΦ^β-cat-FL/FL^ macrophages than in their CD11b-MΦ^β-cat–/–^ counterparts (Fig. 7G i-iii).

Oxygen consumption rate (OCR), an essential indicator of mitochondrial integrity and oxidative phosphorylation efficiency [85], was markedly reduced in infected MΦ^β-cat-FL/FL^ macrophages compared to infected CD11b-MΦ^β-cat–/–^ macrophages (Fig. 7H). This reduction suggests mitochondrial dysfunction, as evidenced by mitophagy in infected MΦ^β-cat-FL/FL^ macrophages (Fig. 4H, K). Infected CD11b-MΦ^β-cat–/–^ macrophages with higher OCR (Fig. 7H) preserved mitochondrial fitness, supporting a stable TCA cycle and maintaining key intermediates (Fig. 7A). Moreover, basal respiration, maximal respiration, spare capacity, and proton leak were significantly lower in infected MΦ^β-cat-FL/FL^ macrophages than in infected CD11b-MΦ^β-cat–/–^ macrophages (Fig. 7H i-iv). These findings highlight the crucial role of β-catenin in orchestrating macrophage metabolic reprogramming during *T. gondii* infection. By promoting glycolysis at the expense of mitochondrial respiration, β-catenin drives a metabolic shift favoring a pro-inflammatory M1-like state, while the enhanced mitochondrial fitness in CD11b-MΦ^β-cat–/–^ macrophages supports an M2 microenvironment, crucial for effective infection control and tissue repair.

### Macrophage-intrinsic β-catenin activation modulates inflammatory response and cytokine expression in CD4^+^ and CD8^+^ T-cell subsets during infection

Till now, we have seen that β-catenin plays an essential role in orchestrating macrophage metabolic reprogramming during *T. gondii* infection. The metabolic shift determines the differentiation pattern of macrophages, crucial for determining the fate of infection. Here we hypothesise that β-catenin activation in macrophages leads to a shift in cytokine production, which in turn influences the differentiation and functional polarisation of CD4⁺ and CD8⁺ T-cell subsets to support infection.

**Fig. 8:**
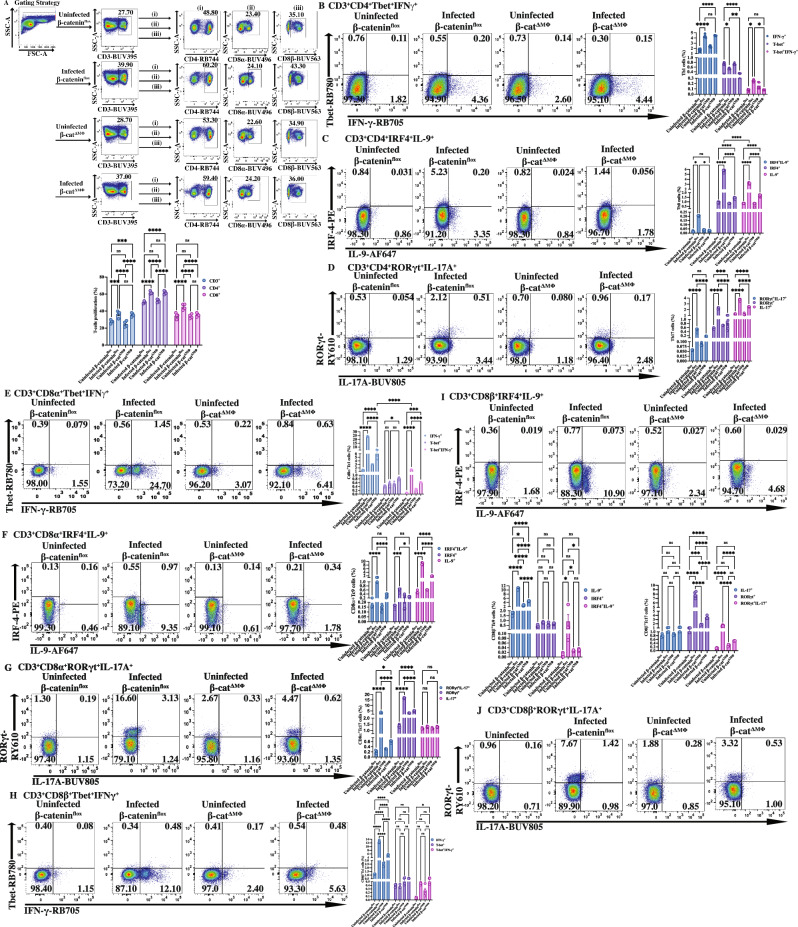
Macrophage-intrinsic β-catenin activation regulates paradigm shift of inflammatory T-cells differentiation, and cytokines profile in CD4^+^ and CD8^+^ T-cell subsets during infection. Macrophages were always collected from uninfected β-catenin^flox^ or β-cat^ΔMΦ^ mice and incubated with CSA for 6 h. Subsequently, macrophages and T-cells were co-cultured under similar conditions. For example, when macrophages were derived from β-catenin^flox^ mice, the splenic CD3⁺ T-cells were also obtained from β-catenin^flox^ mice, either uninfected or infected. After 6 h of co-culture, followed by a 4-hour BFA incubation, the cells were subjected to surface and intracellular staining. Transcription factors and intracellular cytokines were then analyzed using flow cytometry. **A** The gating strategy of CD3⁺CD4⁺ (helper T-cells), CD3⁺CD8α⁺ (cytotoxic T-cells), and CD3⁺CD8β⁺ (cytotoxic T-cells) collected from β-catenin^flox^ and β-cat^ΔMΦ^ mice, either uninfected or infected. The CD3^+^, CD4^+^, and CD8^+^ T-cells proliferation was presented by flow bar plots. Data shown are the mean ± SEM (*n* = 4) with statistical significance assessed using two-way ANOVA. Statistical significance is indicated as ****p < 0.0001, ***p < 0.001, **p < 0.01, *p < 0.05, and “ns” indicating no significance. **B–J** T-cells were categorized based on their surface markers, utilizing a gating strategy of CD3⁺CD4⁺, CD3⁺CD8α⁺, and CD3⁺CD8β⁺ to identify intracellular cytokines and their corresponding transcription factors. These included T-bet⁺IFN-γ⁺, IRF4⁺IL-9⁺, and RORγt⁺IL-17⁺, T-cells from uninfected and infected β-catenin^flox^ or β-cat^ΔMΦ^ mice. The average data of each type of cytokines along with their transcription factors were presented by flow bar plots. Data shown are the mean ± SEM (*n* = 6) with statistical significance assessed using two-way ANOVA. Statistical significance is indicated as ****p < 0.0001, ***p < 0.001, **p < 0.01, *p < 0.05, and “ns” indicating no significance.

To test our hypothesis that macrophage-intrinsic β-catenin is pivotal in driving the generation of pathogenic Th1/Tc1, Th9/Tc9, Th17/Tc17 lineages, as well as regulatory or prophylactic CD4⁺ Treg, CD8⁺ Treg, Th2, and Tc2 lineages, we utilized β-catenin^flox^ and β-cat^ΔMΦ^ mice, infecting them with parasites for 10 days through i.p. BMDMs, identified as CD11b⁺F4/80⁺ double-positive cells, were differentiated separately from both uninfected β-catenin^flox^ and β-cat^ΔMΦ^ mice and treated with CSA. Purified CD3⁺ T-cells were then isolated from the splenocytes of uninfected and infected β-catenin^flox^ and β-cat^ΔMΦ^ mice and co-cultured with these macrophages in 5:1 ratio for 6 h. After an additional 4-hour incubation with Brefeldin A, the cells were harvested for analysis of surface markers, transcription factors, and intracellular cytokine profiles. Initially, the gating strategy for the CD3^+^ subsets of T-cells is described to identify specific sub-populations of CD4, CD8α, and CD8β based on their surface markers across four groups of mice (Fig. 8A). The analysis distinguished CD4^+^ helper T-cells (Th), CD8α^+^ cytotoxic T-cells (Tc), and subsets expressing CD8β also known as cytotoxic T-cells (Tc). This stratification provided approach ensured precise characterization of immune responses in the study. We initially evaluated T-cell proliferation in both uninfected and infected mice, examining the effects of β-catenin presence or absence. Following infection, significant proliferation of CD3⁺, CD4⁺, and CD8⁺ T-cells was observed in T-cells of β-catenin^flox^ infected mice (Fig. 8A bar diagram). Notably, comparable levels of proliferation were detected in T-cells from infected β-cat^ΔMΦ^ mice co-cultured with CD11b⁺F4/80^+^ macrophages deficient in β-catenin in the presence of CSA. Cytokines act as molecular signals that guide T-cells paradigm, ensuring an appropriate immune response tailored to different pathogens, inflammatory conditions, and tissue environments. Hence, we used a cytokine diversity detection approach to ensure precise characterization of immune responses in the study. IFN-γ is a well-established signature cytokine of CD4⁺ T-helper 1 (Th1) and α/β CD8⁺ T-cytotoxic 1 (Tc1) cells, with T-bet serving as a critical transcription factor required for optimal IFN-γ production by both CD4⁺ and α/β CD8⁺ T-cells. Our results revealed a significant increase in CD4⁺IFN-γ⁺ T-cells in both infected β-catenin^flox^ and β-cat^ΔMΦ^ mice (second vs fourth bars, Fig. 8B). However, analysis of CD4⁺T-bet⁺IFN-γ⁺ double-positive T-cells showed higher expression in infected β-catenin^flox^ mice compared to β-cat^ΔMΦ^ infected mice (tenth vs twelfth bars, Fig. 8B). The elevated T-bet expression further reinforces the pro-inflammatory Th1 response, indicating that β-catenin-driven metabolic reprogramming enhances IFN-γ production, thereby promoting effective immune activation. IL-9 is a pleiotropic cytokine primarily produced by Th9 cells, which plays a crucial role in immune responses, influencing inflammation. We observed a significant increase in CD4⁺IL-9⁺ T-cells in infected β-catenin^flox^ mice compared to infected β-cat^ΔMΦ^ mice (tenth and twelfth bars, Fig. 8C). IRF4, an essential transcription factor for IL-9 production [36], was found to be expressed at higher frequencies in IRF4⁺IL-9⁺ double-positive CD4⁺ T-cells in infected β-catenin^flox^ mice than in β-cat^ΔMΦ^ mice (second vs fourth bars, Fig. 8C). The increased presence of CD4⁺IL-9⁺ and IRF4⁺IL-9⁺ T-cells in infected β-catenin^flox^ mice suggests that β-catenin signaling enhances Th9 polarization, potentially contributing to inflammation. IL-17 is a pro-inflammatory cytokine primarily produced by Th17 cells, playing a vital role in autoimmune and inflammatory diseases. Here we found, CD4⁺IL-17⁺ T-cells were significantly more abundant in infected β-catenin^flox^ mice compared to infected β-cat^ΔMΦ^ mice (tenth vs twelfth bars, Fig. 8D). Retinoic acid-related orphan receptor gamma t (RORγt), the critical transcription factor for IL-17 production [37], was expressed at higher frequencies in RORγt⁺IL-17⁺ double-positive CD4⁺ T-cells in β-catenin^flox^ mice than in β-cat^ΔMΦ^ mice (second vs fourth bars, Fig. 8D). Our results demonstrated that CD4⁺IL-17⁺ T-cells were significantly more abundant in infected β-catenin^flox^ mice compared to infected β-cat^ΔMΦ^ mice (tenth vs twelfth bars, Fig. 8D). RORγt⁺IL-17⁺ double-positive CD4⁺ T-cells was expressed at higher frequencies in β-catenin^flox^ mice than in β-cat^ΔMΦ^ mice (second vs fourth bars, Fig. 8D). These findings suggest that β-catenin signaling enhances Th17 differentiation by promoting IL-17 production and RORγt expression, thereby influencing inflammatory immune responses during infection. We also examined CD8α⁺ T-cell subsets and found that IFN-γ⁺CD8α⁺ Tc1 cells were more abundant in infected β-catenin^flox^ mice than in infected β-cat^ΔMΦ^ mice (second and fourth bars, Fig. 8E). Further analysis revealed that CD8α⁺IL-9⁺ Tc9 cells showed similar trends, with significantly higher frequencies in β-catenin^flox^ mice compared to β-cat^ΔMΦ^ mice (tenth vs twelfth bars, Fig. 8F). However, the frequencies of CD8α⁺IL-17⁺ Tc17 cells were comparable between the two groups. Further analysis revealed that CD8α⁺T-bet⁺IFN-γ⁺ Tc1 cells (tenth vs twelfth bars, Fig. 8E), CD8α⁺IRF4⁺IL-9⁺ Tc9 cells (second vs fourth bars, Fig. 8F), and CD8α⁺RORγt⁺IL-17⁺ Tc17 cells (second vs fourth bars, Fig. 8G) were significantly more frequent in infected β-catenin^flox^ mice than in infected β-cat^ΔMΦ^ mice. Similarly, we analyzed CD8β⁺IFN-γ⁺ Tc1 cells and found that their frequencies were higher in infected β-catenin^flox^ mice compared to CD8β⁺ T-cells from infected β-cat^ΔMΦ^ mice (second vs fourth bars, Fig. 8H). Additionally, CD8β⁺IL-9⁺ Tc9 cells were also more abundant in infected β-catenin^flox^ mice than in β-cat^ΔMΦ^ mice (second vs fourth bars, Fig. 8I). However, the frequencies of CD8β⁺IL-17⁺ Tc17 cells were comparable between the two groups (second vs fourth bars, Fig. 8J). Interestingly, while the frequencies of CD8β⁺T-bet⁺IFN-γ⁺ Tc1 cells were similar in infected β-catenin^flox^ and β-cat^ΔMΦ^ mice (tenth vs twelfth bars, Fig. 8H), the frequencies of CD8β⁺IRF4⁺IL-9⁺ Tc9 cells (tenth vs twelfth bars, Fig. 8I) and CD8β⁺RORγt⁺IL-17⁺ Tc17 double positive cells (tenth vs twelfth bars, Fig. 8J) were significantly higher in infected β-catenin^flox^ mice compared to infected β-cat^ΔMΦ^ mice. Our findings highlight the critical role of macrophage-intrinsic β-catenin in shaping T-cell responses during infection. β-catenin^flox^ mice exhibited significantly higher frequencies of pro-inflammatory Th1/Tc1 (IFN-γ⁺, T-bet⁺IFN-γ⁺), Th9/Tc9 (IL-9⁺, IRF4⁺IL-9⁺), and Th17/Tc17 (IL-17⁺, RORγt⁺IL-17⁺) subsets compared to β-cat^ΔMΦ^ mice. This suggests that β-catenin enhances lineage differentiation by modulating cytokine signaling and transcription factor expression. The comparable frequencies of CD8β⁺IL-17⁺ cells indicate a selective role of β-catenin in Th/Tc polarization. Overall, these results underscore β-catenin-driven immune modulation, reinforcing its impact on infection-induced T-cell responses.

### Ablation of macrophage-intrinsic β-catenin shifts towards anti-inflammatory regulatory T-cells lineage commitment to mitigate infection

**Fig. 9:**
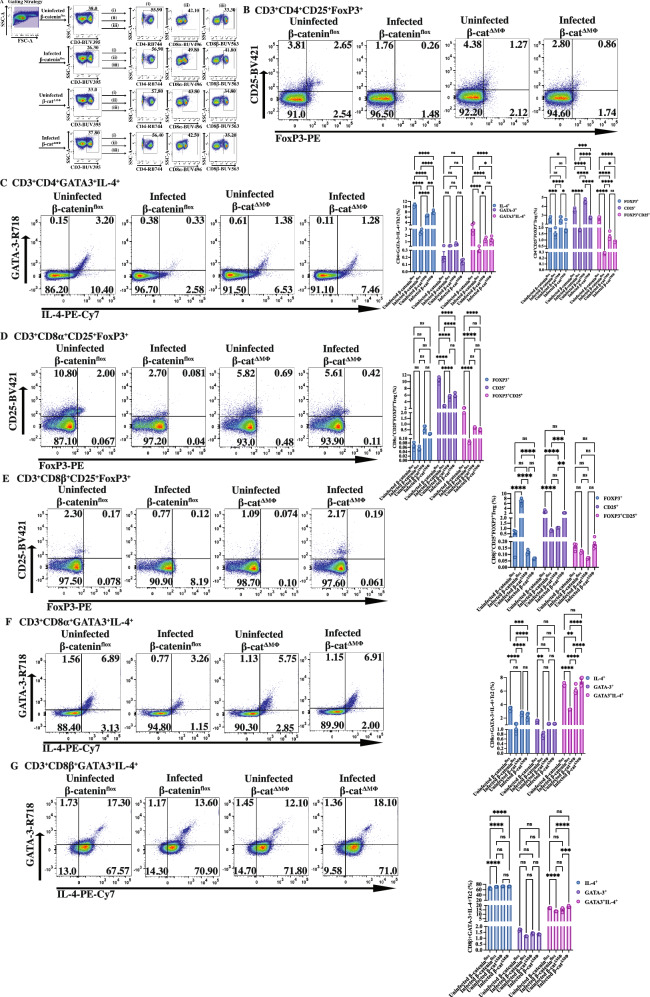
Ablation of macrophage-intrinsic β-catenin reprograms T-cell dynamics toward anti-inflammatory and regulatory lineages to provide protection against infection. Macrophages were always collected from uninfected β-catenin^flox^ or β-cat^ΔMΦ^ mice and incubated with CSA for 6 h. Subsequently, macrophages and T-cells were co-cultured in 1:5 ratio under similar sources and conditions. For example, when macrophages were derived from β-catenin^flox^ mice, the splenic CD3⁺ T-cells were also obtained from β-catenin^flox^ mice, either uninfected or infected. After 6 h of co-culture, followed by a 4-hour BFA incubation, the cells were subjected to surface and intracellular staining. Transcription factors and intracellular cytokines were then analyzed using flow cytometry. **A** The gating strategy was applied to identify CD4^+^, CD8α⁺, CD8β⁺ T-cells collected from β-catenin^flox^ and β-cat^ΔMΦ^ mice either uninfected or infected. **B**–**G** T-cells were categorized further based on their surface markers, utilizing a gating strategy of CD3⁺CD4⁺ (helper T-cells), CD3⁺CD8α⁺ (cytotoxic T-cells), and CD3⁺CD8β⁺ (cytotoxic T-cells) to identify intracellular cytokines and their corresponding transcription factors. These included CD25⁺FoxP3⁺, and GATA3⁺IL-4⁺ T-cells from uninfected and infected β-catenin^flox^ or β-cat^ΔMΦ^ mice. The average data of each type of cytokines along with their transcription factors were presented by flow bar plots. Data shown are the mean ± SEM (*n* = 6) with statistical significance assessed using two-way ANOVA. Statistical significance is indicated as ****p < 0.0001, ***p < 0.001, **p < 0.01, *p < 0.05, and “ns” indicating no significance.

The differentiation of T-cells into distinct inflammatory, anti-inflammatory and regulatory subsets in response to *T. gondii* infection is driven by the need to balance pathogen control and immune regulation to prevent excessive inflammatory damage. As shown previously, during *T. gondii* infection, the immune system mounts a strong inflammatory response, which leads to the differentiation of pro-inflammatory Th1/Tc1, Th9/Tc9, and Th17/Tc17 T-cell subsets. Immune regulation is essential to prevent immunopathology, especially in chronic *T. gondii* infection as it controls excessive immune activation. Anti-inflammatory and regulatory immune cells, including regulatory T cells (Tregs) and regulatory macrophages (M2) produce specific cytokines that help suppress excessive immune responses and maintain immune homeostasis. Regulatory cytokines and their signature transcription factors, such as IL-4 with GATA3 and IL-2 with FoxP3, are primarily involved in immune suppression, tissue repair, and the resolution of inflammation. These factors play a crucial role in protecting individuals from inflammatory diseases [86]. We analyzed the CD3^+^ T-cell subsets by differentiating them based on surface markers CD4, CD8α, and CD8β across four groups of mice. The gating strategy was applied to identify distinct populations within the CD3^+^ T-cell compartment (Fig. 9A). This approach allowed us to evaluate variations in T-cell subsets among experimental groups. The analysis highlights differential T-cell distribution, providing insights into immune modulation. The analysis of regulatory T-cell populations revealed that the frequency of CD4⁺CD25⁺FoxP3⁺ regulatory T-helper (Th-reg) cells was significantly increased in infected β-cat^ΔMΦ^ mice, where β-catenin was ablated, compared to infected β-catenin^flox^ mice (tenth vs twelfth bars, Fig. 9B). In a seminal study demonstrated that GATA3 is both necessary and sufficient for the expression of Th2 cytokines, including IL-4, IL-5, and IL-13, and highlighted its pivotal role in Th2 lineage commitment [87]. We observed the frequencies of both IL-4-secreting cells and GATA3⁺IL-4⁺ double-positive cells were markedly higher in infected β-cat^ΔMΦ^ mice compared to infected β-catenin^flox^ mice (second vs fourth and tenth vs twelfth bars, Fig. 9C). In contrast, no significant differences were observed in the frequencies of CD8α⁺CD25⁺FoxP3⁺ (Fig. 9D) or CD8β⁺CD25⁺FoxP3⁺ (Fig. 9E) regulatory cytotoxic T cells (Tc-reg) between infected β-catenin^flox^ and β-cat^ΔMΦ^ mice. However, the frequencies of CD8α⁺GATA3⁺IL-4⁺ (tenth vs twelfth bars, Fig. 9F) and CD8β⁺GATA3⁺IL-4⁺ (tenth vs twelfth bars, (Fig. 9G) cells were significantly elevated in infected β-cat^ΔMΦ^ mice compared to infected β-catenin^flox^ mice. These findings highlight the critical role of macrophage-intrinsic β-catenin in modulating T-cell differentiation during infection. The presence of β-catenin in macrophages drives pathogenic Th1/Tc1, Th9/Tc9, and Th17/Tc17 lineages. In contrast, β-catenin ablation shifts the immune response toward regulatory Th-reg as well as anti-inflammatory Th2 and Tc2 lineages, characterized by increased FoxP3⁺ Tregs and GATA3⁺IL-4⁺ Th2/Tc2 cells. These results underscore the dual role of β-catenin in shaping the immune response by influencing both inflammatory and regulatory T-cell lineages and determining the fate of infection.

### Tregs from β-cat^ΔMΦ^ mice retain suppressive function during acute *T. gondii* infection

To assess whether CD4⁺CD25⁺FoxP3⁺ T-cells from *T. gondii*-infected β-cat^ΔMΦ^ mice retain their suppressive function, we performed an ex vivo proliferation assay (Fig. S3J). As illustrated in the bar graph, conventional CD4⁺CD25⁻FoxP3⁻ T-cells isolated from uninfected β-catenin^flox^ mice showed robust proliferation upon stimulation with plate-bound anti-CD3 and anti-CD28 antibodies, which has been considered as 100% for comparative analysis. In contrast, CD4⁺CD25⁻Foxp3⁻ T-cells co-cultured with CD4⁺CD25⁺Foxp3⁺ Treg cells sorted from infected β-cat^ΔMΦ^ mice exhibited markedly reduced proliferation (~35%) (Fig. S3J). This significant reduction in proliferative capacity suggests that Tregs from β-catenin-deficient mice retain their suppressive phenotype even during acute *T. gondii* infection. The data imply that the Tregs from β-cat^ΔMΦ^ mice (Fig. 9, S3J), which are relatively resistant to infection (Fig. S1), contribute to an anti-inflammatory environment that enhances host protection and enhances survivability of mice (Fig. S1).

### Macrophage-intrinsic β-catenin ablation shifts T-cells dynamics from inflammatory to regulatory lineages to balance immune response to mitigate infection

To further understand in-depth how β-catenin ablation in macrophages influences immune balance, driving a shift from inflammatory to regulatory T-cell lineages between uninfected and infected groups, uniform manifold approximation and projection (UMAP) analysis was performed. UMAP provides a powerful visualization of T-cell subset dynamics during *T. gondii* infection by identifying inflammatory, anti-inflammatory and regulatory populations, highlighting shifts in T-cell composition between infected and uninfected groups, and correlating cytokine expression with transcription factors. It effectively captures cellular heterogeneity, revealing rare or transitional states that conventional methods might miss. Here, UMAP analysis was done for visualization of expanded T-cell subsets, highlighting (i) inflammatory and (ii) anti-inflammatory cytokines along with their associated transcription factors (Fig. S5A). The results interpreted that, in comparison to uninfected mice (Figs. S5B, i(a–c) and S5Di), the Th1-like Tbet^+^IFNγ⁺ responses are dominated by CD4^+^ T-cells (~20%), along with almost equal contributions from CD8α^+^ (~21%) and CD8β^+^ (~19%) in infected β-catenin^flox^ mice (Figs. S3B, ii(a–c) and S5Dii). This indicates a prominent inflammatory role of both CD4^+^ and CD8^+^ T-cells in parasite multiplication, contrasting with uninfected mice, where Th1-like populations were less pronounced.

Conversely, IRF4^+^IL-9^+^ expression was relatively balanced across T-cell subsets however, CD4^+^ T-cells showing the highest IRF4^+^IL-9^+^ population (~25.5%) (Figs. S5B, ii(g–i) and Dii). While IRF4^-^IL-9^+^ expression was also slightly higher in CD4^+^ (~26.8%) compared to CD8^+^ subsets (~21-22%) (Fig. S5B, ii(j–l) and Dii). This suggests that CD4⁺ T-cells play a dominant role in IL-9-mediated immune responses during infection.

Otherwise, CD8^+^ subsets (CD8α^+^ ~15%, CD8β^+^ ~14.5%) contribute more to double positive RORγt^+^IL-17^+^ expression than CD4^+^ (~13.6%) (Fig. S5B, ii(m-o) and Dii). However, only IL-17^+^ cells negative of RORγt are higher in CD4^+^ (~21%) compared to CD8^+^ (~15-16%) (Fig. S5B, ii(p–r) and Dii). This suggests the existence of distinct inflammatory mechanisms regulating IL-17 expression, which may be independent of RORγt in these subsets. For anti-inflammatory T-cell responses in the infected β-catenin^flox^ mice, CD4^+^ T-cells lead to the regulatory response (~6.2%), while CD8^+^ subsets show minimal regulatory activity (~2.8-5%) (Fig. S5C, ii(a–f) and S2Dii). CD8β^+^ cells show the highest GATA3^+^IL-4^+^ expression, indicating a stronger type-2 immune response in these subsets (Figs. S5C, ii(g–l) and Dii). Hence, CD4^+^ and CD8^+^ T-cells are the main contributors to both inflammatory and regulatory responses, with CD8^+^ subsets playing significant roles in IL-17-driven inflammation. The paradigm between inflammatory (Tbet^+^IFNγ⁺^+^, IRF4^+^IL-9^+^, RORγt^+^IL-17^+^) and anti-inflammatory (CD25^+^FoxP3^+^, GATA3^+^IL-4^+^) cytokines suggests a pro-inflammatory immune environment in the infected β-catenin^flox^ mice.

Further, we have found that the IRF4^+^IL-9^+^ expression is dominated by CD8β^+^ T-cells, where CD4^+^ T-cells contribute less (~15%) in infected β-cat^ΔMΦ^ mice (Figs. S5B, iv(g–l) and S5Div). This indicates an enhanced IL-9-driven inflammation predominantly mediated by CD8^+^ T-cells. Similarly, CD8β^+^ T-cells dominate IL-17 expression (~25%), with CD4^+^ contributing similarly (~25%) (Figs. S5B, iv(m–r) and S5Div) in infected β-cat^ΔMΦ^ mice. But what takes over the precedence are CD4^+^ T-cells, significantly dominating the anti-inflammatory response (~50–55%), with CD8β^+^ contributing (~35%), indicating stronger regulatory immune regulation in infected β-cat^ΔMΦ^ mice.

Analogously, CD8β^+^ T-cells also dominate GATA3^+^IL-4^+^ (~35%), reflecting a shift from inflammation to a regulatory anti-inflammatory milieu in infected β-cat^ΔMΦ^ mice (Fig. S5C, iv(g–l) and Div) to mitigate infection. In infected β-cat^ΔMΦ^ mice, CD4^+^ T-cells primarily drive anti-inflammatory regulation through FoxP3^+^ Tregs (Fig. S5Civ(a–f) and Div), and the immune response shifts towards CD8β^+^ T-cells, which primarily dominate type-2 modulation while also contributing to inflammatory cytokine expression (IFNγ, IL-17, IL-9), albeit to a lesser extent. This reflects that the ablation of macrophage-intrinsic β-catenin indicates a shift in the anti-inflammatory response, with CD8^+^ T-cells becoming the primary drivers in β-cat^ΔMΦ^ mice, where CD4^+^ T-cells are the main contributors to the inflammatory response in β-catenin^flox^ mice. Elevated levels of IFNγ⁺, IL-9⁺, and IL-17⁺ T-cells in β-catenin^flox^ mice highlight the critical role of β-catenin in driving inflammatory responses. In contrast, the higher frequencies of Threg and GATA3⁺IL-4⁺ Th2/Tc2 cells in β-cat^ΔMΦ^ mice indicate a shift toward anti-inflammatory or regulatory pathways. These findings underscore the dual role of β-catenin in orchestrating the balance between inflammatory and regulatory T-cell responses, promoting pathogen clearance while minimizing excessive inflammation.

### Macrophage-intrinsic β-catenin shapes metabolic rewiring in CD4^+^ and CD8^+^ T-cells during ***T. gondii*** infection

During infection, macrophages undergo metabolic reprogramming, switching between glycolysis (pro-inflammatory, M1-like) and OXPHOS (anti-inflammatory, M2-like) states. This shift profoundly influences T-cell metabolism and function through cytokine signaling and metabolite exchange. At the erstwhile section, we observed that M1 macrophages (Fig. 6) drive a glycolytic metabolism (Fig. 7A–C) which fuels inflammatory T-cells differentiation (Th1, Th17, Tc1, Tc17, Th9, Tc9) (Fig. 8) through ROS-HIF-1α stabilization (Fig. 7D), enhanced HKII activity and lactate accumulation (Fig. 7B, C). While M2 macrophages support OXPHOS-dependent anti-inflammatory, regulatory T-cells (Tregs, Th2, Tc2), maintaining immune homeostasis (Figs. 6, 9). While both CD4⁺ and CD8⁺ T-cells undergo metabolic reprogramming during infection, they exhibit distinct metabolic preferences based on their function, activation, and differentiation states. CD4^+^ T-cells primarily rely on glycolysis for rapid energy production and effector functions, particularly during activation after infection, but transition to oxidative phosphorylation in memory states [88, 89]. CD8^+^ T-cells also utilize glycolysis during activation but engage the TCA cycle and oxidative phosphorylation more extensively to support sustained energy demands and cytotoxic activity [90, 91]. To test the hypothesis that macrophage-intrinsic β-catenin regulates key metabolic pathways like glycolysis and the TCA cycle in CD4^+^ and CD8^+^ T-cells, we used β-catenin^flox^ and β-cat^ΔMΦ^ mice, infecting them with *T. gondii* for 10 days. BMDMs, identified as CD11b⁺F4/80⁺ cells, were differentiated from both β-catenin^flox^ and β-cat^ΔMΦ^ mice and treated with CSA. Subsequently, purified CD3⁺ T-cells were isolated from the splenocytes of uninfected and infected β-catenin^flox^ and β-cat^ΔMΦ^ mice and co-cultured with these macrophages for 6 h. Then, CD4^+^ and CD8^+^ T-cells were sorted from co-culture using surface markers and analyzed for metabolites via LC-MS/MS (Fig. S6). In CD4^+^ T-cells of infected β-catenin^flox^ mice, elevated levels of glycolytic intermediates, including glucose-6-phosphate, fructose-1,6-bisphosphate, 3-phosphoglycerate, and lactate, compared to β-cat^ΔMΦ^ mice, suggest an enhanced Warburg effect (Fig. S6A). This heightened glycolytic activity in CD4^+^ T-cells correlates with increased inflammation (Fig. 8B–D). Additionally, higher levels of citrate, succinate, glutamate, and phosphoenol pyruvate coupled with lower levels of α-ketoglutarate, fumarate and malate (Fig. S6A), indicate a disruption in the TCA cycle, or a “broken TCA cycle” in CD4^+^ T-cells of infected β-catenin^flox^ mice (Fig. S6A). In contrast, the metabolic profile of CD4⁺ T-cells from infected β-cat^ΔMΦ^ mice reveals a distinct shift compared to β-catenin^flox^ counterparts, indicating a reduced glycolytic and steady state of TCA cycle (Fig. S6A). The lower levels of glycolytic intermediates like F1,6BP, 3-PG and lactate suggest dampened glycolysis and suppressed Warburg effect in infected β-cat^ΔMΦ^ mice. This shift suggests that CD4⁺ T-cells rely less on glycolysis for energy production, leading to decreased inflammatory potential. Heightened citrate, α-ketoglutarate (α-KG), succinate, fumarate, and malate in CD4⁺ T-cells of infected β-cat^ΔMΦ^ mice suggest a stable state of oxidative metabolism. Along with this, L-glutamine levels were found to be significantly decreased, suggesting lower glutaminolysis. Decreased glutamate but increased α-KG implies the source of α-KG is glucose, which further supports metabolic rewiring towards oxidative metabolism in CD4⁺ T-cells of infected β-cat^ΔMΦ^ mice. Hence, CD4⁺ T-cells from infected β-cat^ΔMΦ^ mice exhibited reduced glycolysis and intact TCA cycle activity, reflecting a metabolic shift away from pro-inflammatory Warburg metabolism. This suggests a shift towards a more regulatory state, potentially contributing to the mitigation of disease (Fig. 9B, C). In summary, β-catenin promotes a pro-inflammatory metabolic state in CD4⁺ T-cells by enhancing glycolysis (Warburg effect) and redirecting TCA cycle intermediates for biosynthesis, fueling rapid expansion and cytokine production in β-catenin^flox^ mice. In contrast, its ablation in β-cat^ΔMΦ^ mice suppresses glycolysis, enhances oxidative phosphorylation, and reduces glutaminolysis, shifting CD4⁺ T-cells toward a regulatory state, potentially favoring a regulatory immune microenvironment and chronic infection control. This metabolic reprogramming underscores β-catenin’s crucial role in inflammation by promoting the Warburg effect to support parasite growth. This metabolic rewiring likely supports effector T-cell activation, and this adaptation facilitates rapid proliferation and effector functions, such as the release of inflammatory cytokines, but compromises energy efficiency. While β-catenin’s ablation fosters an anti-inflammatory environment through OXPHOS, balancing immune activation and abrogation of *T. gondii* infection.

In contrast, CD8^+^ T-cells from infected β-catenin^flox^ mice exhibited elevated levels of both glycolytic intermediates (e.g., glucose-3-phosphate, fructose-1,6-bisphosphate, phosphoenolpyruvate, lactate, and 3-phosphoglycerate) and TCA cycle metabolites (e.g., citrate, succinate, fumarate, malate, oxaloacetate, and phosphoenol pyruvate), as well as amino acids like glutamate and glutamine, compared to CD8^+^ T-cells from β-cat^ΔMΦ^ infected mice (Fig. S6B). This metabolic profile reflects a dual engagement of glycolysis and mitochondrial respiration, indicative of heightened activation. The increased glycolytic flux supports rapid ATP generation and biosynthesis, while TCA cycle engagement, sustained by anaplerotic inputs like glutamine, fuels oxidative phosphorylation and long-term energy production. This metabolic state enables CD8^+^ T-cells to sustain inflammation with higher citrulline in β-catenin^flox^ mice (Fig. S6B), as evidenced by the release of pro-inflammatory cytokines such as IFN-γ, IL-9, and IL-17 (Fig. 8E–J). Elevated lactate levels further confirm active glycolysis, while high TCA intermediates in CD8^+^ T-cells of infected β-catenin^flox^ mice support mitochondrial ATP production and the biosynthesis of macromolecules essential for proliferation and effector functions. The elevated levels of glutamate and glutamine in CD8^+^ T-cells of β-catenin^flox^ mice (Fig. S6B) suggest enhanced glutaminolysis, a critical process that replenishes the TCA cycle. Intermediates like α-ketoglutarate maintain redox balance and ensure robust mitochondrial metabolic phenotype, enabling CD8+ T-cells to meet both immediate and sustained functional demands during activation and effector phases. The metabolic profile of CD8⁺ T-cells from infected β-cat^ΔMΦ^ mice, like CD4⁺ T-cells from infected β-cat^ΔMΦ^ mice, exhibits lower levels of key glycolytic intermediates such as F1,6BP, 3PG, and lactate, suggesting a dampened glycolytic flux with intact OXPHOS state, indicating reduced metabolic flexibility and lower inflammatory potential. Decreased lactate production indicates a shift away from the Warburg effect, reducing rapid ATP generation and biosynthetic precursor supply. This suggests that CD8⁺ T-cells in β-cat^ΔMΦ^ mice rely less on glycolysis for energy and effector function, leading to diminished inflammatory capacity. While the steady-state of citrate, α-KG, fumarate and malate indicates stable OXPHOS and increased mitochondrial energy production in CD8⁺ T-cells of infected β-cat^ΔMΦ^ mice. Overall, β-catenin deletion in macrophages impairs metabolic reprogramming in CD8⁺ T-cells, leading to reduced glycolysis and weakened inflammatory responses, which enforce their ability to combat infection effectively. Thus, macrophage-intrinsic β-catenin modulates T-cell metabolism during *T. gondii* infection, with CD4^+^ T-cells exhibiting a Warburg effect and disrupted TCA cycle, supporting inflammatory cytokine release. Conversely, CD8^+^ T-cells show dual engagement of glycolysis and OXPHOS, driven by glutaminolysis, to sustain prolonged effector functions and inflammation. β-catenin signaling drives a pro-inflammatory metabolic state in both CD4⁺ and CD8⁺ T-cells by enhancing glycolysis and mitochondrial respiration, supporting energy production and cytokine responses. β-catenin^flox^ T-cells maintain metabolic flexibility, while β-cat^ΔMΦ^ T-cells exhibit reduced glycolysis and intact TCA cycle activity, shifting towards a regulatory state. This highlights β-catenin’s role in balancing immune activation during *T. gondii* infection. In summary, these findings emphasize that macrophage-intrinsic β-catenin signaling is associated with distinct metabolic alterations in CD4^+^ and CD8^+^ T-cells, supporting the inflammatory response triggered by parasitic infection.

### PCA and PLS-DA plots reveal β-catenin-dependent metabolic reprogramming in macrophages and T-cells during *T. gondii* infection

To assess the systemic impact of *T. gondii* infection and β-catenin on the metabolite profile of sorted CD4^+^, CD8^+^ T-cells and CD11b^+^/F4-80^+^ macrophage from uninfected β-catenin^flox^, infected β-catenin^flox^, uninfected β-cat^ΔMΦ^, infected β-cat^ΔMΦ^ groups of mice, principal component analysis (PCA) and partial least squares discriminant analysis (PLS-DA) analysis were performed. A comprehensive LC-MS/MS-based metabolomic analysis detected over 1795 metabolites across the different groups. Multivariate analysis was then conducted, starting with PCA to observe differences among groups. The PCA and PLS-DA score plots showed samples of uninfected β-catenin^flox^, infected β-catenin^flox^, uninfected β-cat^ΔMΦ^, and infected β-cat^ΔMΦ^ groups were well separated in CD4^+^ (Fig. S7A(i, ii)), CD8^+^ (Fig. S8A(i–iii)) T-cells and CD11b^+^/F4-80^+^ macrophage (Fig. S9A(i–iii)) cells. To further refine sample differentiation, orthogonal partial least squares discriminant analysis (OPLS-DA) was applied, maximizing group separation. The PCA and OPLS-DA score plots for CD4^+^ (Fig. S7B(i–vi)), CD8^+^ (Fig. S8B(i–vi)) and CD11b^+^/F4-80^+^ macrophages (Fig. S9B(i-vi)) confirmed distinct clustering, revealing significant separation among them. The model’s predictive ability (Q² > 0.7) and classification accuracy (R² > 0.99) indicated strong reliability across all groups.

### Hierarchical clustering reveals β-catenin-dependent metabolic remodeling across immune cell populations during *T. gondii* infection

Hierarchical clustering heatmaps of CD4⁺ T cells (Fig. S7C, D), CD8⁺ T cells (Fig. S8C, D), and CD11b⁺F4/80⁺ macrophages (Fig. S9C, D) revealed distinct metabolic signatures across experimental groups. Uninfected β-catenin^flox^ cells (orange) and uninfected β-cat^ΔMΦ^ (green) represent baseline metabolic states, whereas infected β-catenin^flox^ (pink) and β-cat^ΔMΦ^ (purple) groups reflect infection-induced remodeling in the presence or absence of macrophage β-catenin. In CD4⁺ T cells (Fig. S7D), infection markedly altered the metabolic profile compared to uninfected β-catenin^flox^ controls. Notably, uninfected β-cat^ΔMΦ^ cells displayed a distinct baseline metabolic state, indicating that macrophage β-catenin deletion shapes the metabolic environment even prior to infection. Clustering of uninfected and infected β-cat^ΔMΦ^ groups suggests that β-catenin deficiency is associated with a persistent shift in metabolic state, with infection further modifying this profile. In CD8⁺ T cells (Fig. S8D), both β-catenin^flox^ and β-cat^ΔMΦ^ groups exhibited reduced metabolite abundance upon infection, indicating a global infection-induced metabolic shift. However, differences between genotypes in both uninfected and infected conditions suggest that macrophage β-catenin status influences baseline and infection-associated metabolic states of CD8⁺ T cells. In CD11b^+^/F4-80^+^ macrophages (Fig. S9D), CD11b-MΦ^β-cat-FL/FL^ and CD11b-MΦ^β-cat–/–^ macrophages exhibited distinct basal metabolic profiles. Upon infection, CD11b-MΦ^β-cat-FL/FL^ macrophages showed increased metabolite abundance, consistent with a metabolically active state, whereas CD11b-MΦ^β-cat–/–^ macrophages displayed a comparatively suppressed metabolic profile. Clustering further highlighted genotype-specific metabolic adaptations following infection. Overall, these data demonstrate that β-catenin status shapes both basal and infection-associated metabolic states across immune cell populations. Importantly, the observed changes in T-cell metabolism are consistent with microenvironmental modulation driven by macrophage β-catenin status, rather than direct T-cell-intrinsic metabolic reprogramming. Significant metabolites were defined by VIP > 1.5 and *p* < 0.05.

### Metabolic pathway enrichment analysis reveals macrophage-intrinsic β-catenin-dependent regulation of energy metabolism in CD4^+^ T-cells during *T. gondii* infection

Identified and annotated metabolites in the data were subjected to metabolic pathway and pathway enrichment analysis to identify and elucidate the majorly impacted metabolic pathways among different groups of mice. To identify related metabolic pathways, we used the resources of the KEGG, Human Metabolome Database, and MetaboAnalyst 6.0 (https://www.metaboanalyst.ca/). MetaPathway enrichment analysis was conducted by selecting the “pathway analysis” module, inputting the list of metabolites KEGG IDs, and setting the pathway library to “*Mus musculus* (KEGG)” within the “Mammals” category. The top 25 enriched biological process pathways ranked by *p*-value for CD4^+^ T-cells (Fig. S7E(i–ii)), CD8^+^ T-cells (Fig. S8D(i–iii)) and CD11b^+^F4/80^+^ macrophages (Fig. S9D(i–iii)) were obtained. The results demonstrated that the potential target biological processes were primarily associated with central carbon metabolism, including glycolysis/gluconeogenesis, citrate cycle (TCA cycle) and amino acid metabolism across immune populations, as detailed in Fig. S6C (cut-off impact factor <0.05).

Furthermore, the pathway enrichment analysis was performed comparing metabolic pathways in CD4^+^ T-cells from uninfected β-catenin^flox^ vs infected β-catenin^flox^ (Group 1), uninfected β-cat^ΔMΦ^ vs infected β-cat^ΔMΦ^ (Group 2) and infected β-catenin^flox^ vs infected β-cat^ΔMΦ^ (Group 3) groups of mice based on β-catenin status and *T. gondii* infection.

In Group 1 (Fig. S7E(i)), infection induced a glycolysis-dominant metabolic program, with increased pyruvate metabolism, pentose phosphate pathway activity, and amino acid catabolism. These changes support rapid ATP generation, redox balance, and biosynthetic demands, consistent with an inflammatory effector phenotype. Despite enhanced glycolysis, partial disruption of the TCA cycle suggests a shift toward anabolic metabolism rather than full oxidative phosphorylation. Overall, infected β-catenin^flox^ CD4^+^ T-cells exhibit amino acid utilization, Warburg effect, favoring glycolysis while maintaining partial TCA cycle activity for biosynthesis and immune signaling. This metabolic shift supports rapid energy production, proliferation, and an inflammatory immune response during infection.

In Group 2 (Fig. S7E(ii)), enriched amino acid metabolism (phenylalanine, tyrosine, tryptophan), displayed a restrained metabolic profile, suggests immune regulation and inflammation control. CD4⁺ T cells are characterized by reduced glycolytic activity and greater reliance on mitochondrial and amino acid-dependent pathways. Enhanced TCA cycle engagement and oxidative metabolism, along with altered tryptophan and glutathione pathways, are consistent with a more immunoregulatory or tolerant state. Hence, we can infer that β-catenin ablation in macrophages modulates the metabolic state of CD4^+^ T-cells from a highly active, glycolysis-driven inflammatory phenotype (Group 1) to a restrained, immunosuppressive state (Group 2) by impairing glycolytic efficiency and steady-state of TCA cycle activity, leading to greater reliance on alternative metabolic pathways.

In Group 3 (Fig. S7E(iii)), disrupted amino acid metabolism shifts tryptophan metabolism (IDO pathway) toward immune suppression, aiding *T. gondii* immune evasion. Direct comparison further revealed that β-catenin deficiency in macrophages is associated with reduced carbohydrate metabolism, altered amino acid utilization (including tryptophan and arginine pathways), and a shift toward oxidative metabolism. These changes may limit NO production and nucleotide availability, thereby compromising T-cell effector function and favoring parasite persistence. Overall, β-catenin deletion shapes metabolic state, reducing glycolysis, encouraging TCA cycle activity, and NO production, thereby abrogating parasite infection. This shift fosters *T. gondii* survival by promoting an immunosuppressive tolerant phenotype, emphasizing the critical role of ablation of β-catenin in host defense.

Collectively, these data indicate that macrophage-intrinsic β-catenin signaling modulates the metabolic state of CD4⁺ T cells within the infected microenvironment, promoting a glycolysis-driven inflammatory phenotype under β-catenin-sufficient conditions and a more oxidative, restrained state upon β-catenin loss. Importantly, these findings reflect microenvironmental modulation rather than direct T cell-intrinsic metabolic reprogramming.

### Metabolic pathway enrichment analysis reveals macrophage-intrinsic β-catenin-dependent regulation of energy metabolism in CD8^+^ T-cells during *T. gondii* infection

Furthermore, the pathway enrichment analysis was performed comparing metabolic pathways in CD8^+^ T-cells from uninfected β-catenin^flox^ vs infected β-catenin^flox^ (Group 1), uninfected β-cat^ΔMΦ^ vs infected β-cat^ΔMΦ^ (Group 2) and infected β-catenin^flox^ vs infected β-cat^ΔMΦ^ (Group 3) groups of mice based on β-catenin status and *T. gondii* infection. To assess the influence of macrophage β-catenin, we compared metabolic profiles across infection and genotype conditions. In Group 1 (Fig. S8D(i)), CD8⁺ T cells from infected β-catenin^flox^ mice, infection induced a hybrid metabolic program, characterized by increased glycolysis alongside active TCA cycle and oxidative phosphorylation. Enhanced amino acid metabolism and redox pathways support ATP production, biosynthesis, and effector function, consistent with an activated immune state. Overall, infection drives CD8^+^ T-cells toward glycolysis, along with dependence on oxidative metabolism. The metabolic shift, including altered lipid metabolism and increased amino acid utilization, supports immune activation and pathogen multiplication.

In contrast, in Group 2 (Fig. S8D(ii)), CD8⁺ T cells from β-cat^ΔMΦ^ mice exhibited a reorganized metabolic profile, with increased reliance on glycolysis (Warburg effect) and the pentose phosphate pathway, coupled with reduced engagement of oxidative metabolism. This shift supports biosynthetic and redox demands but suggests altered integration of mitochondrial metabolism.

Direct comparison (Group 3; (Fig. S8D(iii)) further revealed that macrophage β-catenin deficiency is associated with altered carbohydrate utilization, amino acid metabolism, and lipid remodeling pathways, alongside changes in mitochondrial function. These features are consistent with modified metabolic fitness that may impact T-cell proliferation, persistence, and immune responsiveness. Collectively, these findings indicate that macrophage-intrinsic β-catenin signaling modulates CD8⁺ T-cell metabolic states within the infected microenvironment, shaping their bioenergetic and functional programs without implying direct T cell-intrinsic metabolic reprogramming.

### Metabolic pathway enrichment analysis reveals macrophage-intrinsic β-catenin-dependent regulation of energy metabolism in macrophages during *T. gondii* infection

Next, the pathway enrichment analysis was performed comparing metabolic pathways in macrophages from uninfected CD11b-MΦ^β-cat-FL/FL^ vs infected CD11b-MΦ^β-cat-FL/FL^ (Group 1), uninfected CD11b-MΦ^β-cat–/–^ vs infected CD11b-MΦ^β-cat–/–^ (Group 2) and infected CD11b-MΦ^β-cat-FL/FL^ vs infected CD11b-MΦ^β-cat–/–^ (Group 3) based on β-catenin status and *T. gondii* infection.

In Group 1 (Fig. S9D(i)), pathway enrichment analysis in macrophages reveals extensive metabolic reprogramming in response to infection. In β-catenin^flox^ macrophages, infection induced a glycolysis-dominant metabolic program, accompanied by enhanced amino acid metabolism (e.g., arginine, tyrosine) and redox pathways. Increased glycolysis and lipid remodeling are consistent with a pro-inflammatory, metabolically active phenotype, supporting rapid energy production and immune signaling. In Group 2 (Fig. S9D(ii)), pathway enrichment analysis in CD11b-MΦ^β-cat–/–^ macrophages exhibited a shift toward oxidative metabolism, characterized by reduced glycolysis and increased TCA cycle activity, mitochondrial function, and redox balance. Enhanced amino acid catabolism and lipid remodeling suggest a metabolically adaptive state that may influence immune regulation and host-pathogen interactions. Direct comparison (Group 3; Fig. S9D(iii)) further highlighted β-catenin-dependent differences in metabolic pathway utilization. β-catenin^flox^ macrophages showed increased engagement of glycolysis, amino acid metabolism, and lipid pathways, whereas CD11b-MΦ^β-cat–/–^ macrophages displayed greater reliance on mitochondrial oxidative metabolism and associated redox pathways. These differences underscore distinct metabolic configurations associated with β-catenin status. Collectively, these findings indicate that β-catenin signaling orchestrates macrophage metabolic states during infection, promoting a glycolysis-associated inflammatory profile in β-catenin-sufficient cells and a more oxidative metabolic configuration in its absence.

## Discussion

Our findings partially align with previous research suggesting that *T. gondii* manipulates host signaling pathways, particularly the PI3K-AKT-β-catenin axis, to promote its replication and survival [11, 92]. Prior studies have shown that *T. gondii* effector proteins activate the host PI3K-AKT pathway, resulting in the suppression of ROS production through CYBB inhibition [10]. Nevertheless, our data present a comprehensive and contrasting effect, demonstrating that the activation of the PI3K-AKT-β-catenin pathway (Figs. 1, 2) through TLR11/12 independent fashion by *T. gondii* (Fig. 5I) increases ROS production (Fig. 4G), mediated by CYBB activation (Fig. 4F). This elevated ROS contributes to mitophagy dysfunction at later time points (Figs. 4I and S3D–F) and a heightened NLRP3-mediated inflammatory response, indicating a more complex host-parasite interaction. In this study, we confirm and extend these observations, showing that *T. gondii* induces phosphorylation of PI3K (Tyr458), AKT (Ser473), and β-catenin (Ser552), which are critical for parasite growth. Inhibition of PI3K (using copanlisib), AKT (capivasertib), or β-catenin (XAV939) disrupts this cascade, impairing parasite replication and mature PVs formation, highlighting a hierarchical PI3K-AKT-β-catenin signaling axis. Notably, inhibition of β-catenin alone, despite active PI3K and AKT, is sufficient to impede *T. gondii* replication, underscoring its central role in host-parasite interaction. Our data are consistent with earlier studies [93, 94], showing that *T. gondii* hijacks host signaling pathways to maintain its intracellular niche. We further demonstrate that phosphorylation of β-catenin at Ser552 is essential for *T. gondii* replication in macrophages (Fig. 2A), whereas β-catenin-deficient macrophages (CD11b-MΦ^β-cat–/–^) exhibit impaired replication, reinforcing its functional importance. Inhibition of β-catenin via XAV939 destabilizes host β-catenin (Fig. S2), promotes UPS-mediated degradation (Fig. 2F), and reduces parasite burden (Fig. 2D, E). Consistently, CD11b-MΦ^β-cat–/–^ macropahges display enhanced resistance to *T. gondii* (Fig. 2L), highlighting β-catenin as a potential therapeutic target, further supported by reduced infection-associated pathology. Mechanistically, our study further extends on β-catenin transcriptional activity by identifying a β-catenin-IRF4-CYBB signaling axis regulating ROS production. The β-catenin-TCF4 complex drives IRF4 transcription, which in turn upregulates CYBB (NOX2), leading to ROS generation and modulation of mitochondrial dynamics. This axis promotes mitophagy at early stages (6 h.p.i., Fig. S3C), while sustained ROS contributes to mitochondrial dysfunction at later stages (Fig. S3D–F), facilitating parasite persistence. Genetic ablation of β-catenin disrupts this axis, abolishing ROS production and impairing *T. gondii* replication. Collectively, our findings establish β-catenin as a central regulator linking PI3K-AKT signaling, ROS generation, and mitochondrial dynamics, thereby coordinating immune modulation and parasite survival.

We observed phosphorylation-dependent activation of PINK1 and PARKIN in *T.* *gondii-*infected macrophages, consistent with initiation of mitophagy at early time points. Notably, PINK1/PARKIN activation persisted at later stages (Fig. 4H, I); however, mitophagy markedly declined by 12–24 h.p.i. (Fig. S3C), indicating a defect in downstream mitophagic flux. This bottleneck, likely exacerbated by sustained ROS accumulation, permits the persistence of damaged mitochondria, leading to mtDNA oxidation (Fig. S3D), cytosolic release of mtDNA at later stages of infection (24 h.p.i.) (Fig. S3E), and depletion of mtDNA copy number (Fig. S3F). These changes are accompanied by bioenergetic collapse, as evidenced by reduced oxygen consumption rate (OCR), elevated extracellular acidification rate (ECAR) (Fig. 7G, H), and disruption of TCA cycle intermediates (Fig. 7A). By 24-30 h.p.i., unresolved mitochondrial stress and oxidized mtDNA are associated with inflammasome activation and gasdermin D mediated pyroptosis (Fig. 4J), marking a temporal shift from mitochondrial quality control to inflammatory cell death. Together, these findings support a model in which β-catenin-dependent signaling coordinates a dynamic mitochondrial stress response during *T. gondii* infection. In the early phase of infection, CYBB-driven ROS promotes activation of the PINK1/PARKIN pathway, leading to induction of mitophagy. This likely represents an adaptive mechanism to eliminate damaged mitochondria and maintain cellular homeostasis in infected macrophages. However, as infection progresses, sustained ROS production and increasing mitochondrial damage appear to exceed the capacity of the PINK1/PARKIN-mediated quality control system. This is reflected by reduced mitophagy, accumulation of fragmented mitochondria, and subsequent activation of gasdermin D-mediated pyroptosis. These observations are consistent with a threshold-dependent transition from a protective to a pro-inflammatory response, rather than a discrete regulatory switch. While our data establish a link between mitochondrial stress, impaired mitophagy, and pyroptotic cell death, the precise molecular mechanisms governing this transition remain to be fully defined. In particular, the potential roles of LC3-dependent autophagic flux and parasite-derived effector proteins in modulating mitophagy at later stages warrant further investigation. Such studies, including the use of parasite mutants and targeted functional analyses, are beyond the scope of the present work but represent important directions for future research.

The absence of β-catenin in CD11b-MΦ^β-cat–/–^ cells may disrupt mitochondrial recruitment and metabolic support, leading to reduced parasite growth. The disintegration of mitochondria during the release of tachyzoites (24–30 h.p.i.) proposes a parasite-induced alteration of mitochondrial integrity, likely mediated by oxidative stress or parasite effector proteins. This aligns with prior reports showing that *T. gondii* infection induces mitochondrial dysfunction as part of its egress mechanism [95]. Our findings reveal a critical role for β-catenin in modulating host-pathogen interactions during *T. gondii* infection, linking ROS to mitochondrial damage in parasitic infections. β-catenin also regulates inflammasome activation, a key component of the immune response to pathogens [96]. We show that *T. gondii* exploits β-catenin to drive AIM2 activation, followed by NLRP3 inflammasome activation, leading to caspase-1 activation, IL-1β secretion, and gasdermin D-mediated pyroptosis. Crucially, phosphorylation of β-catenin at S552 is essential for this process. Mitochondrial clustering around PVs facilitates parasite replication, while their subsequent disintegration during egress releases oxidized mtDNA, amplifying AIM2-NLRP3 inflammasome signaling in a TLR11/12 independent fashion signifying a novel innate immune sensing mechanism. Despite mitochondrial damage, the intact nucleus in pyroptotic cells implies selective cellular disintegration, creating a microenvironment that favors infection progression and immune evasion. In the absence of β-catenin, inflammasome activation, pyroptosis, and parasite growth are significantly suppressed, highlighting its critical role in linking metabolic and immune responses. These findings underscore how *T. gondii* manipulates β-catenin to subvert host defenses, providing insights into the intersection of mitochondrial dynamics, inflammasome activation, and immune regulation during parasitic infections. In addition to modulating immune responses, β-catenin plays a central role in mouse macrophage polarization during *T. gondii* infection. Using THP-1 cells as a TLR11/12-deficient human model, we demonstrate that AIM2-mediated NLRP3 activation is independent of TLR11/12 signaling. While the mechanistic ROS-mtDNA-AIM2 pathway is defined in mouse macrophages, future studies in primary human systems will be required to fully establish its conservation.

Oxidized DNA was detected exclusively in the cytoplasmic fraction of infected cells (Fig. S3E), absent in nuclear fractions and uninfected controls, confirming a mitochondrial origin. At 24 h post-infection, mitochondrial disintegration (Video S1C) coincided with elevated cytoplasmic oxidized mtDNA, supporting AIM2 activation. Consistently, PINK1 and PARKIN were enriched in mitochondrial fractions, while phosphorylated NLRP3 localized to the cytosol. NAC treatment suppressed PINK1/PARKIN activation (Fig. 4I), AIM2 activation, and NLRP3 phosphorylation (Fig. 4N), and AIM2 knockdown further reduced p-NLRP3 (Fig. 4M), establishing a causal link between ROS-driven mitochondrial damage, oxidized mtDNA release, and AIM2-mediated optimal NLRP3 inflammasome activation. Together, these findings support a model in which β-catenin phosphorylation and ROS signaling converge to regulate AIM2, which in turn contributes to NLRP3 inflammasome activation. Importantly, although AIM2 knockdown reduced NLRP3 phosphorylation, residual activation persisted, indicating that NLRP3 is regulated by multiple parallel pathways [24, 53] during *T. gondii* infection. In this context, NLRP3 activation may also occur through AIM2-independent mechanisms driven by infection-associated cellular stress signals, including mitochondrial dysfunction or ROS generation. Notably, the more pronounced activation of NLRP3 in the presence of intact AIM2 suggests that AIM2 provides a secondary or amplifying effect rather than functioning as a primary upstream regulator.

Our findings also identify β-catenin phosphorylation at Ser552 as a critical regulatory event linking inflammasome activation with mitochondrial quality control during *T*. *gondii* infection. While initial observations suggested an association between β-catenin activation and downstream signaling, our rescue experiments using phosphorylation-deficient (S552A) and phosphomimetic (S552D) mutants establish a causal requirement for S552 phosphorylation. Specifically, reconstitution of β-catenin-deficient macrophages with the S552D mutant restored inflammasome signaling as well as PINK1-PARKIN activation, whereas the S552A mutant failed to rescue these pathways. These findings demonstrate that S552 phosphorylation is essential for coordinating both inflammasome responses and mitophagy during infection. Although S552 phosphorylation has been associated with β-catenin nuclear localization, our data support its role as a key regulatory modification required for β-catenin-dependent transcriptional and signaling functions, rather than defining it as the sole driver of nuclear translocation. Collectively, our study positions β-catenin S552 phosphorylation as a central node integrating immune and mitochondrial signaling pathways in macrophages during parasite infection.

Our findings further demonstrate that mitophagy, pyroptosis, and inflammation are temporally interconnected in *T. gondii*-infected macrophages, with β-catenin as a key regulator. Early in infection (6 h p.i.), β-catenin-dependent ROS impairs mitochondrial health, triggering protective mitophagy to limit ROS and maintain homeostasis, thereby sustaining a niche for parasite replication (Fig. S3A). By 12-24 h.p.i., mitophagy becomes dysfunctional, coinciding with mitochondrial fragmentation (Fig. 4K, Video S1C), mtDNA loss (Fig. S3F), and metabolic collapse, reflected by reduced OCR, elevated ECAR (Fig. 7G, H), and depletion of TCA intermediates (Fig. 7A). This metabolic reprogramming amplifies ROS (Fig. 4G) and drives oxidative mtDNA damage (Fig. S3D). By 30 h.p.i., unresolved stress and oxidized mtDNA accumulation activate inflammasomes and gasdermin D-mediated pyroptosis (Fig. 4K, Video S1C), marked by mitochondrial disintegration, intact nuclear morphology, and parasite egress.

Together, these results reveal a threshold-dependent transition from protective mitophagy to pro-inflammatory pyroptosis, linking mitochondrial quality control and metabolic state to cell fate, and highlighting β-catenin’s dual role in sustaining parasite replication early and promoting host immune activation later in infection.

Our findings reveal that IL-1β production in macrophages is critically dependent on β-catenin-mediated antigen presentation. This is underscored by the markedly reduced IFN-γ production from T-cells when β-catenin is absent in macrophages (Fig. 5L), indicating a failure in effective T cell activation. In co-cultures of OT-II TCR transgenic T-cells with MΦ^β-catFL/FL^ macrophages pulsed with the OVA_323-339_ peptide, we observed robust IL-1β secretion (Fig. 5N). In contrast, both IL-1β and IFN-γ secretion were significantly impaired when β-catenin was genetically ablated (CD11b-MΦ^β-cat–/–^) or pharmacologically inhibited with XAV939 (Fig. 5J, L, N), confirming that β-catenin is indispensable for initiating effective antigen-specific immune responses. These observations highlight the dual role of β-catenin in modulating inflammasome activation and facilitating cognate antigen presentation required for T cell effector function. Specifically, the absence of β-catenin compromises macrophage capacity to present antigen effectively, resulting in reduced MHC-TCR interaction avidity and diminished downstream IL-1β and IFN-γ responses. Notably, β-catenin-deficient macrophages failed to activate antigen-primed T cells, as shown by the lack of IFN-γ production in co-cultures with γ-irradiated, CSA-treated macrophages (Fig. 5L), indicating a failure in signal delivery necessary for T cell polarization. Collectively, these results position β-catenin as a central regulator of innate-adaptive immune crosstalk. By enhancing MHC-TCR interaction strength, β-catenin facilitates both inflammasome complex assembly (via IL-1β release) and T cell activation (via IFN-γ production). Importantly, our data support the “signal three” hypothesis, wherein cytokine-mediated cues that determine T cell fate are strongly influenced by the β-catenin status in macrophages. This places β-catenin at a pivotal checkpoint linking the quality of antigen presentation to the strength and specificity of downstream inflammatory and adaptive immune responses.

Our findings demonstrate that LDHA inhibition suppresses Glut1, iNOS, and IL-1β expression (Fig. S3G–I), thereby impairing M1 polarization during infection (Fig. S5E). This highlights lactate as a critical metabolite sustaining the M1 phenotype and supporting parasite growth. Furthermore, β-catenin-driven lactate production emerges as a key determinant of M1 polarization, fueling T-cell-dependent inflammation and parasite replication. Conversely, β-catenin depletion promotes an M2-like anti-inflammatory phenotype with reduced inflammasome activity and preserved mitochondrial integrity, limiting tissue damage but potentially compromising host defense. These findings underscore the pivotal role of nuclear phospho-β-catenin as a transcription factor in M1 polarization, contrasting earlier studies highlighting cytosolic β-catenin in M2 polarization, and establishing its role in chronic inflammation during *T. gondii* infection [97]. We further demonstrate that β-catenin regulates macrophage metabolic reprogramming, governing the shift between M1 and M2 states. M1 macrophages rely on aerobic glycolysis via the CYBB-ROS-HIF1α-HKII axis (Warburg effect), exhibiting elevated succinate and citrate levels that drive inflammasome activation and cytokine production [75]. In contrast, M2 macrophages depend on oxidative phosphorylation and fatty acid oxidation to support anti-inflammatory functions [98]. The absence of β-catenin in CD11b-MΦ^β-cat–/–^ macrophages reduces glycolysis and preserves mitochondrial integrity, favoring an anti-inflammatory state. Seahorse XF analysis confirms that β-catenin enhances glycolysis and impairs mitochondrial pathways in M1 macrophages, whereas its absence promotes mitochondrial fitness and M2 polarization. β-catenin also critically regulates T-cell differentiation during *T. gondii* infection. It promotes pro-inflammatory Th1/Tc1, Th9/Tc9, and Th17/Tc17 responses producing IFN-γ, IL-9, and IL-17 [99, 100]. Although IFN-γ-mediated GTPase activation facilitates PV lysis [101], parasite egress (Video S1A) enables reinfection and systemic dissemination. This process is further amplified by cytokines including TNF-α, IL-12, IL-6, and IL-23 from macrophages, and IFN-γ, IL-9, and IL-17 from T-cell subsets (Figs. 7, 9). In contrast, β-catenin depletion favors regulatory T-cells, increasing Th-reg/Tc-reg and Th2/Tc2 populations, thereby promoting an anti-inflammatory response [87].

Mechanistically, β-catenin interacts with IRF4 to regulate T-cell differentiation. In Th17/Tc17 cells, β-catenin enhances IRF4 and RORγt activation [102, 103], while in Th1/Tc1 cells, it cooperates with T-bet to drive IFN-γ production [104, 105]. IRF4 also supports Th9/Tc9 differentiation and IL-9 production [106, 107]. This β-catenin-IRF4 axis is fundamental in immune regulation and inflammation. Additionally, AIM2 interacts with IRF4 to modulate T-cell response [108]. β-catenin further regulates T-cell metabolism: CD4⁺ T-cells from β-catenin^flox^ mice display a pronounced Warburg effect with disrupted TCA cycle, whereas CD8⁺ T-cells maintain balanced glycolysis and mitochondrial respiration, supporting cytotoxic function [89, 91]. Collectively, these findings establish β-catenin as a central regulator of immune polarization, metabolic reprogramming, and T-cell differentiation, driving parasite survival and persistence in toxoplasmosis. Finally, a key future goal is to elucidate how *T. gondii*-induced neuroinflammation, oxidative stress, and mitochondrial dysfunction contribute to cognitive impairment resembling Alzheimer’s disease, particularly through dysregulated mitophagy. To enhance translational relevance, we plan to extend these findings to human-relevant systems, including humanized immune models such as NSG (NOD SCID gamma) mice engrafted with human hematopoietic stem cells. These models will enable evaluation of host-pathogen interactions and neuroimmune responses in a setting that more closely reflects human physiology. In parallel, we will assess the therapeutic potential of targeting β-catenin using pharmacological inhibitors such as XAV939. Given that current evidence for XAV939 is largely limited to murine preclinical studies, future work will focus on evaluating its efficacy, safety, and pharmacokinetic properties in humanized systems. While targeting β-catenin represents a promising host-directed strategy to modulate infection-associated inflammatory and metabolic pathways, its central role in tissue homeostasis warrants careful consideration of potential off-target effects and systemic toxicity. Therefore, therapeutic approaches will likely require precise delivery strategies and controlled dosing regimens, such as short-term or intermittent inhibition, to achieve an optimal balance between efficacy and safety.

## Materials and methods

### Reagents

The following chemicals were obtained from commercial sources and used as indicated: (i) AMBMP hydrochloride (Tocris, 6043), a Wnt agonist used as a positive control for β-catenin activation, was reconstituted to a final concentration of 10 mM in 2 μl DMSO, which did not exhibit any cell toxicity. (ii) Copanlisib (Sigma, A199526), a pan-PI3K inhibitor, was used at a concentration of 100 nM in DMSO. (iii) UCL-TRO-1938 (Sigma, A2305184), a PI3K agonist, was used at 30 µM in 1.8 μl DMSO, with no observed cell toxicity. (iv) XAV939 (Tocris, 3748; Sigma, X3004) was reconstituted to a final concentration of 50 μM in 2.6 μl DMSO for in vitro culture. For in vivo experiments, XAV939, Wnt-C59 (Tocris, 5148), and JW55 (Tocris, 4514) were administered intraperitoneally in mice at a final concentration of 4mg/kg body weight, with the final volume adjusted to 100μl in PBS. (v) recombinant murine M-CSF (Peprotech, 31502). (vi) chicken egg albumin peptide OVA_323-339_ (Invivogen; vac-isq) was dissolved in H_2_O to a final concentration of 10 μM. (vi) N-acetyl-L-cysteine (NAC, Sigma, A7250), vii. Phorbol 12-myristate 13-acetate (PMA, Sigma, P8139).

### Parasites

The tachyzoite stage RH parasites were transfected with a plasmid containing the cDNA of the 2-methylisocitrate lyase gene (*Tg*p*rpB*) with a 3’ fusion to the GFP gene. Expression of *TgprpB-gfp* was driven by plasmid-encoded *T. gondii* beta-tubulin promoter [68]. A plasmid encoding the *dhfr* selection cassette was co-transfected along with the *TgprpB-gfp* plasmid, and selection was done with pyrimethamine. Clonal isolates were obtained from the stable population by the limiting dilution technique and used in further studies. GFP-tagged *T. gondii* tachyzoites (Type-I RH strain) were propagated following established protocols [18]. The RHΔku80asp5-3Ty strain, which was expressed with Ct-ASP-Lox-P-GFP-HXGPRT linearized AvrII, was transfected with 5’ASP5-pTub8-loxP-KillerRed-Ble linearized XhoI to create the RHΔku80loxPasp5-3Ty strain. 40 μg of pTub5-Cre was transfected in this strain to obtain RHΔku80Δasp5 (*T. gondiiΔ*^*ASP5*^) [109, 110], which was sensitive to antimicrobials such as MPA-Xa and Pyrimethamine. The parasites were cultured in human foreskin fibroblast cells and purified by differential centrifugation at 3000 × g for 10 min. The resulting pellet was resuspended in phosphate-buffered saline (PBS), and parasite counts were determined using a hemocytometer. For ex vivo or in vitro infections, parasites were applied at a multiplicity of infection (MOI) of 3 across different cell types. For purification of cysts of *T. gondii* ME49, brains were harvested from chronically infected (8 weeks post-infection) C57BL/6 mice under sterile conditions. Each brain was homogenized in 1 mL cold PBS using a 16 G needle, followed by serial passage through 18 G and 20 G needles to ensure mechanical dissociation. The homogenate was filtered through a 70 µm cell strainer to remove debris. Cysts were enriched by low-speed centrifugation (50 × *g* for 5 min at 4 °C) and washed twice with PBS. For further purification, Percoll gradient (30%/90%) centrifugation at 1200 × *g* for 15 min was used, and the cyst-containing interface was collected. Purified cysts were washed and counted under a phase-contrast microscope. To determine whether the macrophage-intrinsic mechanisms identified in vitro translate to in vivo infection and influence adaptive immune responses, we extended our analysis to mouse models and subsequent T-cell metabolic profiling to understand how macrophage β-catenin-dependent signals shape T-cell plasticity.

### Mice

The RH strain tachyzoites, a virulent Type I strain, were used in all experiments. Infected mice were euthanized on specified days for tissue analyses, with organs harvested for various cell-based assays. All animal procedures were approved by the Institutional Animal Ethics Committee (IAEC#586/21 & IAEC/DS/PN/2023/199). For in vivo *and* ex vivo infection experiments, we used C57BL/6 J wild-type (WT) mice, B6.OT-II (JAX:004194) mice, IFN-γ knockout (JAX:002287) mice, and IRF4 knockout (JAX:031834) mice, all originally obtained from the Jackson Laboratory and maintained in the National Institute of Immunology core mouse breeding facility. *ctnnb1*^flox/flox^ mice (β-catenin^flox^) were originally gifted from IIT, Kanpur, and was maintained in B6 background. β-catenin^flox^ mice were crossed with transgenic mice (B6.129P2-Lyz2tm1-cre-Ifo/J) expressing Cre recombinase under the control of a lysozyme 2 gene (Lyz2) promoter, to generate mice lacking β-catenin in monocytes, mature macrophages and granulocytes (β-cat^ΔMΦ^). Successful cre-mediated deletion was confirmed by polymerase chain reaction (PCR) and protein expression analyses. Mice were allocated randomly to experimental groups, and no blinding was done. For in vivo studies, 50 tachyzoites of the RH strain were infected through i.p. in Wt, β-catenin^flox^ and β-cat^ΔMΦ^ mice. Control mice received PBS alone through i.p. Similarly, mice were also orally infected with 100 cysts of the *T. gondii* ME49 strain to induce acute infection. Cysts were isolated from the brains of chronically infected donor mice, homogenized in sterile PBS, and counted microscopically using a coverslip-based wet mount. Each mouse received 100 cysts in 200 µL sterile PBS via oral gavage using a blunt-end feeding needle [40, 111]. Animals were monitored daily for clinical symptoms, body weight loss, and overall health status. The experiment was terminated at day 15 post-infection. At the endpoint, mice were euthanized, and tissues, including the spleen, were collected for downstream analyses. Parasite growth was monitored through immunoblotting using an antibody specific to *T. gondii* (SAG1) and by qPCR with SAG1-targeted primers [18]. To determine the most effective β-catenin pathway inhibitor for enhancing resistance to *T. gondii* infection and reducing infection-associated weight loss, three compounds, XAV939, JW55, and Wnt-C59, were selected based on their known ability to modulate Wnt/β-catenin signaling. Mice were infected, and drug treatment was initiated on the 2nd day (i.e., 1 day post-infection). Each inhibitor was administered via the i.p. route once daily for seven consecutive days at a dose of 4 mg/kg body weight per day, determined from their respective LC₅₀ values.

### Preparation of bone marrow-derived macrophages (BMDMs)

Following erythrocyte lysis, bone marrow cells from Wt, β-catenin^flox^ and β-cat^ΔMΦ^ mice were isolated and enriched for CD11b^+^ cells using the Mouse CD11b^+^ Selection Kit II (18970, Stemcell Technology). The enriched CD11b^+^ cells were then resuspended in RPMI1640 medium supplemented with 10% heat-inactivated FBS and 100 ng/ml recombinant mouse M-CSF (complete medium) at a density of 1 × 10^6^ cells/ml and cultured for 7 days. These adherent macrophages' characterization and sorting were performed using flow cytometry, where CD11b-BUV395 (BD Biosciences, 563553) was used for the initial gating, followed by F4/80-BV786 (BD Biosciences, 744340) for the secondary gating, as surface markers. CD11b^+^F4/80^+^ sorted macrophages from Wt mice were referred to as Wt-MΦ, while those derived from β-catenin^flox^ mice were designated as MΦ^β-cat-FL/FL^, and CD11b^+^F4/80^+^ cells from β-catenin conditional knockout (β-cat^ΔMΦ^) mice were termed CD11b-MΦ^β-cat–/–^, which are absent of *ctnnb1* gene. In all ex vivo and in vitro experiments, whether explicitly mentioned or not, CD11b^+^F4/80^+^ double-positive cells were consistently differentiated and sorted from the bone marrow of Wt, β-catenin^flox^, and β-cat^ΔMΦ^ mice. Similarly, BMDMs were isolated from IFN-γ knockout (JAX:002287) and IRF4 knockout (JAX:031834) mice and after 7 days of differentiation, adherent BMDMs were harvested for experiments.

### Cell culture and infection

Mouse macrophages (Wt-RAW 264.7), Wt-MΦ, MΦ^β-cat-FL/FL^, and CD11b-MΦ^β-cat–/–^ were used for infection at an MOI of 3. *T. gondii* RH strains were grown in human foreskin fibroblast cells and purified by differential centrifugation (3000 × *g*, 10 min) [18, 110]. Parasites were suspended in phosphate-buffered saline (PBS), counted in a hemocytometer under a microscope, and used for infection in different cells at an MOI of 3. The parasite growth was measured by both immunoblot using *T.* *gondii-*specific antibody (SAG1) as well as qPCR using specific primers. mCherry was tagged endogenously with hexokinsase of *T. gondii* and was grown in the presence of pyrimethamine.

### Plasmids and transfection study

The FLAG-tagged wild-type (Wt)-β-catenin, phospho-mutant S552A-β-catenin, and constitutively active-S552D-β-catenin constructs were kind gifts from Zhimin Lu, University of Texas M. D. Anderson Cancer Center, USA [112]. CD11b-MΦβ-cat–/– cells were transfected with Effectene transfection reagent (Qiagen; 301425) following the manufacturer’s instructions. Unless otherwise stated, 0.8 μg plasmid was used for transfection per 5 × 10^5^ cells in each 4 cm^2^ well of the plate. After 8 h post-transfection, cells were infected with *T. gondii* at an MOI of 3.

### Reporter assay

To prepare the minimal promoter (MinP) construct containing the TCF/LEF-binding site of the IRF4 promoter (GCACCTGC), a nonspecific 2600 bp PCR product was cloned into the pGL3 plasmid between NheI and HindIII sites to generate MinP-PGL3. The MinP sequence (32 bp) was then inserted 68 bp upstream of the luciferase gene by inverse PCR. Clones were confirmed by the ~2 kb fragment generated upon restriction with EcoRI and BamHI, since the EcoRI site is present in the MinP sequence and the BamHI site is in the vector backbone. The confirmed vector (MinP-PGL3) was used for insertion of the 73 bp of TCF/LEF binding sites (GCACCTGCgggtaGCACCTGCgggtaGCACCTGCgggtaGCACCTGCgggtaGCACCTGCgggtaGCACCTGC) at two different positions, i. 50 bp upstream of MinP sequence ii. 2500 bp upstream of MinP sequence by inverse PCR. Here, TCF/LEF binding sites are mentioned in CAP letters, and a spacer in lower case, separating each copy of the TCF/LEF site. These two insertions were cloned by blunt-end ligation, and the final clone was confirmed by restriction digestions. M50 Super 8x Top-Flash (Top-Flash, addgene-12456), where seven TCF/LEF binding sites are present at upstream of firefly luciferase reporter, and M51 Super 8x Fop-Flash (Top-Flash mutant, addgene-12457), where mutated TCF/LEF binding sites are present at upstream of luciferase reporter, were used for luciferase reporter assay. [18]

To prepare the MinP-PGL3 construct containing the IRF4-binding site of the CYBB promoter, the above-mentioned MinP-PGL3 construct was used for insertion of the 79bp of IRF4 binding site (GAAACTGAAgggtaGAAACTGAAgggtaGAAACTGAAgggtaGAAACTGAAgggtaGAAACTGAAgggtaGAAACTGAA) at two different positions, i. 50bp upstream of the MinP sequence, ii. 2500 bp upstream of the MinP sequence by inverse PCR. Here, IRF4 binding sites are mentioned in CAP letters, and a spacer in lower case, separating each copy of the IRF4 site. These two insertions were cloned by blunt-end ligation, and the final clone was confirmed by restriction digestions. RAW-264.7 cells were seeded in 2 cm2 well of a plate, and were transfected with 50 ng of the luciferase reporter plasmid together with a total of 200 ng of various expression plasmids or empty control plasmids and pCMV-Renilla-Luc vectors (50 ng). The Renilla luciferase plasmid served as an internal control in all luciferase assays. After 8 h of transfection, *T. gondii* was infected for 12 h, and then the luciferase activity in the total cell lysate was measured by using the Dual-Luciferase Reporter Assay kit (Promega). All experiments were performed in triplicate.

### Inflammation-related host gene targeting in RAW 264.7 cells

Mouse Ctnnb1 (Gene ID: 12387; chromosome location 9F4) targeting CRISPR/Cas9 knock-out plasmid was designed to disrupt gene expression of Wt-RAW 264.7 by causing a double-strand break (DSB) in a 5′ constitutive exon within the CTNNB1 gene. The β-catenin CRISPR/Cas9 KO plasmid kit (Santacruz; sc-419477-KO-2) consists of a pool of 3 plasmids, each encoding the Cas9 nuclease and a target-specific 20 nt guide RNA (gRNA) designed for maximum knockout efficiency. The β-catenin HDR plasmid (h) (Santacruz, sc-419477-HDR-2) was used for the selection of cells containing a DSB induced by β-catenin CRISPR/Cas9 KO Plasmid to get β-catenin^–/–^ macrophages. The mouse IRF4 (Entrez Gene ID: 16364; chromosome location 13A3.2) was also targeted CRISPR/Cas9 KO plasmid was designed in a similar manner (Santacruz, sc-421148, & sc- 421148-HDR). At 12 h post-transfection, IRF4^–/–^ macrophages were positively selected in puromycin (2 μg/ml) for 72 h, and the culture was maintained in the presence of puromycin. The deletion of the gene in both β-catenin^–/–^ and IRF4^–/–^ macrophages was confirmed by both immunoblotting and PCR. We generated ASC1^–/–^, Caspase-1^–/–^, and NLRP3^–/–^ macrophages from Wt-RAW 264.7 cells using CRISPR/Cas9 knockout and HDR plasmids obtained from Santa Cruz (ASC1: sc-425141, sc-425141-HDR; Caspase-1: sc-419461-KO-2, sc-419461-HDR-2; NLRP3: sc-432122, sc-40027-HDR).

### siRNA-mediated gene silencing and parasite infection in Wt-MΦ

For gene knockdown, Wt-MΦ were transfected with 50-100 nM of mouse-specific siRNAs (Santa Cruz Biotechnology) using Lipofectamine RNAiMAX (Thermo Fisher Scientific), following the manufacturer’s protocol. The siRNAs used were: PI3K p110α (sc-39128), AKT1 (sc-29196), CCL19 (sc-60002), Rock-2 (sc-36433), CYBB (sc-35504), AIM2 (sc-140968), TLR11 (sc-61694), and LDH-A (sc-45898). Complexes were prepared in Opti-MEM, incubated for 15-20 min, and added to cells for 6 h. For TLR11 silencing, Wt-RAW 264.7 cells were transfected with TLR11 siRNA (sc-61694). Cells were then cultured in fresh medium for 24-48 h before infection with *T. gondii* tachyzoites at the indicated MOI. Knockdown efficiency was validated by qRT-PCR. All experiments were performed in triplicate and independently repeated.

### Immunoprecipitation (IP) and immunoblot

For immunoprecipitation, after transfection in 9 cm^2^ well of the plate, and followed by infection, cells were lysed in lysis buffer containing 20 mM HEPES (pH 7.4), 50 mM NaCl, 1.5 mM MgCl_2_, 2 mM DTT, 2 mM EGTA, 10 mM NaF, 12.5 mM β-glycerophosphate, 1 mM Na3VO_4_, 5 mM Na_4_P_2_O_7_, 0.2% (v/v) Triton X-100, and protease inhibitors (Thermo-Fischer Scientific, 78425). Cell lysates, containing 400 μg of total protein, were precleared with mouse immunoglobulin G (IgG) agarose (Sigma) in IP buffer for 1 h, and then incubated overnight with IP-specific antibodies, followed by protein A/G beads for 1 h with rotation. After incubation, the beads were washed with 1 Å~ PBS, and protein complexes were eluted by adding 40 μl of 2 Å~ sample buffer to each IP reaction and heating at 50 °C for 15 min. For immunoblot, cells were lysed in the 1.5 Å~ Laemmli sample buffer containing protease inhibitor cocktails and for phospho-antibody, phosphatase inhibitors cocktail (Cell Signaling Technology, 5870) was additionally used. After sonication, samples were heated at 95 °C, and an equal amount of proteins was analyzed on denaturing SDS-polyacrylamide gels after estimation of protein concentration. The proteins were transferred to PVDF blotting membrane (Amersham Hybond, 10600029) and probed with specific primary antibody, followed by horseradish peroxidase (HRP) conjugated secondary antibody. Bands were visualized by a based detection system (Amersham) using SuperSignal West Femto/Pico Plus substrate (Thermo-Fischer Scientific). The intensity of protein bands was quantified using ImageJ software, NIH, USA, and β-actin was used as a control for normalization. Statistical bar diagram of each immunoblot was generated from three or more repeat experiments after normalizing with the β-actin band. For immunoblotting and immunostaining, the following antibodies were used. From Cell Signaling Technology, we employed: I. phospho-PI3K (Tyr458, Cat. No. 4228), II. PI3K (4292), III. phospho-AKT (Ser473, 9271), IV. pan-AKT (4691), V. phospho-β-catenin (Ser552, 9566), VI. β-catenin (9562), VII. β-actin (4970), VIII. NLRP3 (13158), IX. AIM2 (63660), X. phospho-PINK1 (Ser228, 89010), XI. cleaved Gasdermin D (36425), XII. ubiquitin (3936), XIII. cleaved IL-1β (63124), and XIV. cleaved Caspase-1 (89332). From Abcam, we used XV. *T. gondii* SAG1/p30 (ab8313) and XVI. histone H3 (1791). From Sigma, XVII. FLAG (F3165) was used. From Santa Cruz Biotechnology, the following antibodies were obtained: XVIII. tankyrase-1/2 (sc-365897), XIX. ASC (sc-514414), XX. mouse secondary-HRP (sc-2031), and XXI. rabbit secondary-HRP (sc-2030). From Thermo Fisher Scientific, we used XXII. phospho-NLRP3 (Ser295, PA5-105071), XXIII. phospho-IRF4 (Tyr122/Tyr125, PA5-105214), XXIV. CYBB/NOX2 (PA5-79118), XXV. PINK1 (PA1-16604), XXVI. phospho-PARKIN (Ser65, PA5-114616), XXVII. PARKIN (702785), and XXVIII. VDAC (4866). Finally, from Cloud-clone, XXIX. IRF4 (PAB755Mu01) was included.

### Ubiquitination assay

To analyze the ubiquitination of β-catenin, CD11b-MΦβ-cat^–/–^ cells were transfected with FLAG-tagged plasmids encoding wild-type β-catenin (FLAG-Wt-β-catenin), the S552A mutant (FLAG-β-catenin-S552A), or the S552D mutant (FLAG-β-catenin-S552D). After 12 h of transfection, the cells were pre-treated with the proteasome inhibitor MG132 (10 μM) for 4 h, followed by infection with parasites for 6 h in the presence or absence of XAV939, while maintaining MG132 treatment. MG132 concentration used in our study did not have any detectable effect on parasite viability. Cell lysates were then immunoprecipitated using an anti-FLAG antibody, and the immunoprecipitated samples were analyzed by immunoblotting with an anti-ubiquitin antibody.

### Quantitative real-time PCR

Total RNA from macrophages was isolated using TRI Reagent (Sigma), and the concentration was measured. The purity and integrity of RNA were checked after DNase treatment. cDNA was made from 1μg of RNA using the Revert Aid First Strand cDNA Synthesis Kit (Thermo Fisher Scientific). For qPCR, the PCR mixture (total volume 6 μl) was prepared containing 3 μl of SYBR AmpliTaq Gold DNA Polymerase (ABI), 1 μl of cDNA, forward and reverse primers (0.2 μl each) and DEPC water (1.6 μl). The primers used for qPCR are listed below. For β-catenin- Fwd: AGAACCCCTTGGATATCGCC, Rev: TGGCCACCCATCTCATGTTC, IRF4- GTGGAAACACGCGGGCAAGC, and rev, GGCTCCTCTCGACCAATTCCTCA, CYBB- Fwd: TGGCGATCTCAGCAAAAGGTGG, Rev: GTACTGTCCCACCTCCATCTTG, NLRP3- Fwd: TCACAACTCGCCCAAGGAGGAA, Rev: AAGAGACCACGGCAGAAGCTAG, AIM2- Fwd: AGGCTGCTACAGAAGTCTGTCC, Rev: TCAGCACCGTGACAACAAGTGG, HIF1α- Fwd: T CCTGCACTGAATCAAGAGGTTGC, Rev: CCATCAGAAGGACTTGCTGGCT, HKII- Fwd: TGATCGCCTGCTTATTCACGG, Rev: AACCGCCTAGAAATCTCCAGA, LDH-A- Fwd: ACGCAGACAAGGAGCAGTGGAA, Rev: ATGCTCTCAGCCAAGTCTGCCA, MT-ND1- Fwd: GTTGGTCCATACGGCATTTT, Rev:TGGGTGTGGTATTGGTAGGG, ND5- Fwd: CGGAGACATCGGATTCATTT, Rev: GAGGCCAAATTGTGCTGATT, HK2- Fwd: GCCAGCCTCTCCTGATTTTAGTGT, Rev: GGAACACAAAAGACCTCTTCTGG, Glut1 (Slc2a1)-Fwd: GCTGTGCTTATGGGCTTCTC, Rev: AGGGTAGAGTCAGGGAGGAG, iNOS (Nos2)- Fwd: CAGCTGGGCTGTACAAACCTT, Rev: CATTGGAAGTGAAGCGTTTCG, Arginase-1- Fwd: CTCCAAGCCAAAGTCCTTAGAG, Rev: AGGAGCTGTCATTAGGGACATC, CHI3L3-Fwd: CTGAATGAAGGAGCCACTGA, Rev: CCAGCTGGTACAGCAGACAA, IL-1β- Fwd: TGGACCTTCCAGGATGAGGACA, Rev: GTTCATCTCGGAGCCTGTAGTG and GAPDH for, TGTGTCCGTCGTGGATCTGA, and rev, CCTGCTTCACCACCTTCTTGAT. The comparative Ct (ΔΔCt) expression of target genes using Real-Time PCR (ABI ViiA7; Applied Biosystems, USA), and β-actin was used as the internal calibrator. The fold change in expression was used as a relative measure of gene expression. For determining the growth of *T. gondii*, qPCR was performed using primer pair, against the SAG1 gene conserved in all *T. gondii* strains: SAG1- Fwd: ATCGCCTGAGAAGCATCACTG, Rev: CGAAAATGGAAACGTGACTGG. Primers for *the T. gondii* SAG1 gene did not generate a product when the genomic DNA was used as a template derived from uninfected cells; β-actin was used as a control.

### Molecular docking and structural analysis of mammalian and ***T. gondii*** PI3K, AKT, and TNKS Homologs

The human TNKS1-XAV939 co-crystal structure (PDB: 3KR8) was used as a reference protein-ligand complex. The mouse TNKS1 sequence was obtained from UniProt (ID: Q6PFX9), and the homologous *T. gondii* TNKS1 sequence (UniProt ID: A0A086L1P2) was identified via BLAST against the NCBI non-redundant protein database. Due to the absence of experimental structures for the mouse and *T. gondii* proteins, AlphaFold-predicted structures were utilized. Active site residues were identified by multiple sequence alignment of the human, mouse, and *T. gondii* TNKS1 sequences using Clustal Omega, and structurally validated within the AlphaFold-predicted models.

Protein preparation was performed using the Protein Preparation Wizard in Maestro (Schrödinger, v12.8.117). Protonation states were assigned at pH 7.0 using PROPKA, and hydrogen atoms were added accordingly. The structures were energy-minimized using the OPLS4 force field with convergence set to an RMSD of 0.3 Å for heavy atoms.

The structure of XAV939 was sketched in 2D using ChemDraw (v22.2) and saved in SDF format. The molecule was converted to its 3D conformation using the 3D Builder module in Maestro. Ligand preparation was conducted with LigPrep (Schrödinger) to enumerate possible tautomers and ionization states at physiological pH (7.0 ± 0.5) using the Epik module. Known stereochemistry was retained, and energy minimization was carried out with the OPLS4 force field. The optimized ligand conformers were exported in SDF format for docking studies.

Receptor grids were generated using the Receptor Grid Generation panel in Glide, with the grid centered on the identified active site residues and a cubic box of 15 Å. Molecular docking was performed using the Extra Precision (XP) mode in Glide. The resulting ligand poses were ranked by docking score and evaluated by visual inspection for key molecular interactions. Protein-ligand interactions were analyzed and visualized using PyMOL.

The human PI3K-copansilib co-crystal structure (PDB: 5G2N) was used as a reference protein-ligand complex. The mouse PI3K sequence was obtained from UniProt (ID: Q9JHG7), and the homologous *T. gondii* PI3K sequence (UniProt ID: A0A086PJ86) was identified via BLAST against the NCBI non-redundant protein database. Due to the absence of experimental structures for the mouse and *T. gondii* proteins, AlphaFold-predicted structures were utilized. Active site residues were identified by multiple sequence alignment of the human, mouse, and *T. gondii* PI3K sequences using Clustal Omega, and structurally validated within the AlphaFold-predicted models.

Protein preparation was performed using the Protein Preparation Wizard in Maestro (Schrödinger, v12.8.117). Protonation states were assigned at pH 7.0 using PROPKA, and hydrogen atoms were added accordingly. The structures were energy-minimized using the OPLS4 force field with convergence set to an RMSD of 0.3 Å for heavy atoms.

The structure of Copansilib was sketched in 2D using ChemDraw (v22.2) and saved in SDF format. The molecule was converted to its 3D conformation using the 3D Builder module in Maestro. Ligand preparation was conducted with LigPrep (Schrödinger) to enumerate possible tautomers and ionization states at physiological pH (7.0 ± 0.5) using the Epik module. Known stereochemistry was retained, and energy minimization was carried out with the OPLS4 force field. The optimized ligand conformers were exported in SDF format for docking studies.

Receptor grids were generated using the Receptor Grid Generation panel in Glide, with the grid centered on the identified active site residues and a cubic box of 15 Å. Molecular docking was performed using the Extra Precision (XP) mode in Glide. The resulting ligand poses were ranked by docking score and evaluated by visual inspection for key molecular interactions. Protein-ligand interactions were analysed and visualized using PyMOL.

The human AKT-capivacertib co-crystal structure (PDB: 4GY1) was used as a reference protein-ligand complex. The mouse AKT sequence was obtained from UniProt (ID: P31750), and the homologous *T. gondii* AKT sequence (UniProt ID: A0A139Y3Z4) was identified via BLAST against the NCBI non-redundant protein database. Due to the absence of experimental structures for the mouse and *T. gondii* proteins, AlphaFold-predicted structures were utilized. Active site residues were identified by multiple sequence alignment of the human, mouse, and *T. gondii* AKT sequences using Clustal Omega, and structurally validated within the AlphaFold-predicted models.

Protein preparation was performed using the Protein Preparation Wizard in Maestro (Schrödinger, v12.8.117). Protonation states were assigned at pH 7.0 using PROPKA, and hydrogen atoms were added accordingly. The structures were energy-minimized using the OPLS4 force field with convergence set to an RMSD of 0.3 Å for heavy atoms.

The structure of capivacertib was sketched in 2D using ChemDraw (v22.2) and saved in SDF format. The molecule was converted to its 3D conformation using the 3D Builder module in Maestro. Ligand preparation was conducted with LigPrep (Schrödinger) to enumerate possible tautomers and ionization states at physiological pH (7.0 ± 0.5) using the Epik module. Known stereochemistry was retained, and energy minimization was carried out with the OPLS4 force field. The optimized ligand conformers were exported in SDF format for docking studies.

Receptor grids were generated using the Receptor Grid Generation panel in Glide, with the grid centered on the identified active site residues and a cubic box of 15 Å. Molecular docking was performed using the Extra Precision (XP) mode in Glide. The resulting ligand poses were ranked by docking score and evaluated by visual inspection for key molecular interactions. Protein-ligand interactions were analysed and visualized using PyMOL.

### Microscopic study

Both MΦ^β-cat-FL/FL^ and CD11b-MΦ^β-cat–/–^ macrophages were cultured on glass-bottom dishes and infected with GFP-tagged *T. gondii* tachyzoites (MOI: 3:1). Live imaging was performed using an LSM980 NLO confocal microscope equipped with an environmental chamber (37 °C, 5% CO₂). Images were acquired using a 63x oil-immersion objective at specified time points post-infection to capture intracellular dynamics. Time-lapse imaging was conducted with z-stack acquisition and minimal photobleaching using optimal laser settings. For fixed imaging, macrophages were further seeded on glass coverslips and infected with GFP-parasites (MOI: 3:1). Post-infection, cells were incubated with MitoTracker Red to label mitochondria, followed by fixation with 4% paraformaldehyde. Permeabilization was performed using 0.1% Triton X-100, and samples were blocked with 5% BSA. Coverslips were mounted with Prolong Gold antifade medium containing DAPI (Thermo-Fischer Scientific, P36931). Imaging was conducted using an LSM980 NLO confocal microscope with a 63x oil-immersion objective. Z-stacks were acquired to assess mitochondrial morphology and PV formation. For localization of targeted protein, cells were incubated with primary antibodies against β-catenin and IRF4, followed by Alexa Fluor-conjugated secondary antibodies. Nuclei were stained with DAPI. Coverslips were mounted on slides using an antifade mounting medium. Confocal imaging was conducted using a confocal laser scanning inverted microscope (Carl-Zeiss, LSM980 NLO) with a 63x oil-immersion objective. Z-stack images were acquired to determine protein localization in the cytoplasm, nucleus and mitochondria and analyzed using Zen Blue software. All data were processed with Ziess Zen 3.8 software for quantitative analysis, ensuring accuracy in tracking host-pathogen interactions and cellular responses. For confocal quantitative imaging, five fields of view were captured per biological replicate, with a total of three biological replicates.

### Purification of nuclear, cytoplasmic fractions, mtDNA, and genomic DNA

Cells were transfected with FLAG-tagged plasmids encoding wild-type β-catenin (FLAG-Wt-β-catenin), phospho-mutant β-catenin (FLAG-β-catenin-S552A), or constitutively active β-catenin (FLAG-β-catenin-S552D). After 12 h of transfection, the cells were infected with *parasites*. At various time points post-infection, cells were harvested, and nuclear and cytoplasmic fractions were isolated using the NE-PER nuclear and cytoplasmic extraction reagents kit (Thermo Fisher Scientific, 78833). Additionally, phosphorylated IRF4 levels in nuclear and cytoplasmic fractions were analyzed following parasite infection to assess its subcellular distribution and activation status. Mitochondrial fractions were similarly purified following the manufacturer’s protocol (Thermo Fisher Scientific, 89874). Mitochondrial DNA (mtDNA; Abcam, ab65321) and nuclear DNA (nDNA; Abcam, ab65358) were purified from uninfected and infected cells following the manufacturer’s protocols. Purified mtDNA and nDNA from uninfected and infected cells were also used for transfection in uninfected Wt-MΦ macrophages to evaluate AIM2 activation.

### Extraction of TgESPs and TEM

The RH strain of *T. gondii* and *T. gondii*^ΔASP5^ were propagated in HFF cells. The parasites were harvested, rinsed in PBS, and centrifuged at 1000 × *g* for 5 min at room temperature, a process repeated twice. Purified tachyzoites were then suspended in 1 ml of Hank’s balanced salt solution (Gibco, Thermo Fisher Scientific, Inc.) and subjected to oscillation at 3.57 × g in a constant-temperature shaker set at 37 °C for 4 h. Subsequently, the samples were centrifuged three times at 14,000 × *g* for 5 min each at 4 °C, and the resulting supernatant, referred to as TgESPs, was collected and stored. To visualize vesicle-like structures in the TgESPs, the samples were deposited onto a nickel grid, stained with uranyl acetate and lead citrate, and imaged using a Tecnai G2 20 Twin transmission electron microscope.

### Whole transcriptome analysis of experimental conditions

Whole transcriptome analysis was conducted on 12 biological samples across four experimental conditions: uninfected control, infected, uninfected treated with XAV939 (4 mg/kg body weight), and infected treated with XAV939 (4 mg/kg body weight), with three biological replicates per condition. Sequencing was performed using the Illumina platform with 2 × 150 bp paired-end chemistry, generating high-quality raw data between 12.21 Gb and 20.41 Gb per sample (raw reads ~ 20-60 million). Raw reads were processed using Trimmomatic (v0.39) to remove adapter sequences [113], ambiguous reads (>5% unknown nucleotides), and low-quality bases (>10% with Phred scores <25). Reads shorter than 100 nucleotides were discarded. Additional filters included a sliding window (10 bp, quality threshold 25) and trimming of low-quality bases (<25) at read ends. High-quality reads were mapped to the *Mus musculus* reference genome (mm39) using the STAR aligner [83] (v2.7.10a), yielding mapping efficiencies from 53.8% (infected + XAV939) to 96.65% (uninfected). Differential gene expression (DGE) was analyzed using DESeq2, with volcano plots visualizing DEGs [97]. Fold changes (X-axis) were color-coded (upregulated in red, downregulated in green), while the Y-axis displayed the negative log-transformed P-values (*p* ≤ 0.05); non-significant genes appeared in black. KEGG pathway enrichment was performed using ClusterProfiler (v4.2.2) [114], highlighting pathways with p < 0.05, and summarizing the top 30 pathways. This workflow integrates preprocessing, read alignment, feature quantification, normalization, DEG identification (padj < 0.05), visualization, and pathway enrichment to provide valuable insights into transcriptomic variations and biological pathways.

### Ubiquitination assay

To analyse the ubiquitination of β-catenin in the presence of XAV939, CD11b-MΦ^β-cat–/–^ were transfected with FLAG-Wt-β-catenin and after 12 h of transfection, the cells were pre-treated with proteasome inhibitor (MG132, 10 μM) for 4 h, and then infected with parasites at different time points. The cell lysates were immunoprecipitated with FLAG antibody and the immunoprecipitated samples were analysed by immunoblot, using anti-ubiquitin.

### Measurement of ROS

In addition to their critical role in ATP synthesis, mitochondria are also a major source of ROS in cells. Cells were seeded in 96-well plates and were infected with parasites according to the experimental design. ROS levels were quantified at 12 h and 24 h after the infection using the Cellular ROS detection assay kit (Abcam, ab186027), according to the manufacturer’s protocol. The red dye used in the ROS assay protocol is cell-permeable and generates red fluorescence when it reacts with H_2_O_2_, released from cells. The fluorescence at λ ex/em 520/605 nm was measured by a microplate spectrofluorometer (Biotek PowerWave XS Microplate Reader).

### Quantification of oxidative DNA damage in subcellular fractions using 8-OHdG ELISA

To assess oxidative DNA damage across cellular compartments, nuclear and cytoplasmic fractions were first separated using the nuclear and cytoplasmic Extraction Kit (Gbiosciences,786182), following the manufacturer’s protocol. The nuclear and cytoplasmic fractions were collected for downstream DNA extraction, while the mtDNA from the mitochondrial fraction was obtained independently using the mitochondrial DNA isolation kit (Abcam, AB65321). Cytoplasmic DNA (cDNA) and nuclear DNA (nDNA) were extracted using the DNA Isolation Kit (Promega), ensuring high-quality DNA suitable for oxidative damage quantification. DNA purity and concentration were assessed using a NanoDrop spectrophotometer (Thermo Fisher Scientific). Oxidative damage was quantified by measuring 8-hydroxy-2′-deoxyguanosine (8-OHdG), a well-established biomarker of ROS-induced DNA lesions. The 8-OHdG content in each DNA fraction (tDNA, nDNA, mtDNA) was determined using a competitive ELISA kit (Elabscience, E-EL-0028) according to the manufacturer’s instructions. Briefly, 2 µg of purified DNA was denatured at 95 °C for 5 min, rapidly cooled on ice, and then applied to the ELISA plate. Absorbance was measured at 450 nm using a microplate reader, and 8-OHdG concentrations were calculated from a standard curve. All measurements were performed in technical triplicates, and results were normalized to DNA concentration and presented as ng 8-OHdG per µg DNA.

### Measurement of relative mtDNA copy number

Relative mitochondrial DNA copy number (mtDNAcn) was determined by qPCR using SYBR Green chemistry. The mitochondrial gene MT-ND1 and ND5 were amplified and normalized to the nuclear reference gene HK2. Total DNA from uninfected and infected macrophages after 24 h.p.i., containing both nuclear, cytoplasmic and mitochondrial genomes, was extracted from sample fractions and quantified spectrophotometrically. qPCR reactions were performed in triplicate using the SciPhi qPCR Master Mix (Nextgen) on a Real-Time PCR System. The cycling protocol consisted of an initial denaturation at 95 °C for 10 min, followed by 40 cycles of 95 °C for 15 s and 60 °C for 60 s. A melt-curve analysis was performed at the end of the run to verify amplicon specificity. Relative mtDNA/nDNA ratios were calculated using the ΔCt method, where ΔCt = Ct_MT-ND1_-Ct_HK2_.

### Sample preparation for metabolomics analysis

MΦ^β-cat-FL/FL^ macrophages (CD11b^+^F4/80^+^ double-positive cells) from β-catenin^flox^ mice and CD11b-MΦ^β-cat–/–^macrophages from β-cat^ΔMΦ^ mice were differentiated as mentioned above and seeded at a density of 1 × 10⁵ cells per well. The cells were infected with live RH strain parasites for 6h, followed by the collection of samples for untargeted metabolomics. For T-cells metabolomics, β-catenin^flox^ and β-cat^ΔMΦ^ mice were infected with the RH strain via the i.p. route as mentioned above. 10 days post-infection, splenocytes were isolated and subjected to red blood cell (RBC) lysis. CD3⁺ T-cells were subsequently sorted using CD3ε-BUV395 (BD Biosciences, 569614) via flow cytometry. CD11b^+^F4/80^+^ gated macrophages, as mentioned above, were prepared from the two groups of mice: MΦβ-cat-FL/FL macrophages from β-catenin^flox^ mice and CD11b-MΦβ-cat^–/–^ macrophages from β-cat^ΔMΦ^ mice. These macrophages were incubated with CSA (40 μg/well) derived from the RH strain (*T. gondii*) for 6 h. Following this, the purified CD3⁺ T-cells, sorted from uninfected and infected splenocytes of both β-catenin^flox^ and β-cat^ΔMΦ^ mice, were co-cultured with the macrophages at a ratio of 5:1 for an additional 6 h. Then, CD4^+^ and CD8^+^ T-cells were sorted using specific antibodies (CD4 RB744 Rat Anti-Mouse, and Ms CD8b BUV563 H35-17.2) from co-culture and analyzed for metabolites via LC-MS/MS. Macrophages and CD4^+^ and CD8^+^ T-cells were immediately quenched by placing them in -20 °C pre-cooled 80% methanol (5 mL). After thorough removal of methanol, 1 mL of 80% (v/v) cold methanol was quantitatively added. Two washes with 2 mL of 80% cold methanol were performed, and the washed solutions were collected. Intermittent ultrasound (sonication for 1 s with 1 s interval) was performed for 3 min with a cellular sonicator in a water bath. 2 ml of the prepared sample was taken and vacuum dried at 4 °C. 1 ml of pre-cooled methanol/acetonitrile/water solution (2:2:1, v/v/v) was added and vortexed for mixing, then centrifuged at 14,000 g for 20 min at 4 °C. The supernatant was taken and vacuum dried at 4 °C. Before mass spectrometry analysis, 100 μl of aqueous acetonitrile solution (acetonitrile: water = 1:1, v/v) was added for re-dissolution, vortexed for mixing, and then centrifuged at 14,000 g for 15 min at 4 °C. The final 100 μl supernatant was reconstituted in 95:5:5 standard solution (95% water: 5% internal standards: 5% acetonitrile) and was then subjected to reverse-phase chromatography in a C18 column (Thermo Scientific™25003102130: 3 µm, 2.1 mm, 100 mm) using an ultra-high-performance liquid chromatographic system, followed by high-resolution orbitrap mass spectrometry (HRMS).

### Data processing and differential metabolites analysis

Compound Discoverer (Thermo Scientific™ Compound Discoverer™) software [115] was used to preprocess the LC/MS raw data, annotate features using mass spectra mzCloud™ (www.mzcloud.org) and mzVault (Thermoscientific) and generate a three-dimensional data matrix in CSV format. This matrix contained key information, including sample details, metabolite names, and mass spectrum response intensities. Metabolites were identified by querying databases, such as HMDB (http://www.hmdb.ca/), Metlin (https://metlin.scripps.edu/), and the Majorbio database. Identified metabolites were then annotated. First, metabolite peaks were identified and processed for denoising based on relative standard deviation. Subsequently, peak with the highest area under the curve was chosen. Data analysis incorporated the normalization method. The resulting dataset was imported into MetaboAnalyst 6.0 (https://new.metaboanalyst.ca/docs/About.xhtml) for multivariate analysis. Annotated features were filtered based on non-parametric relative standard deviation (MAD/median) and were subjected to log normalization and pareto-scaling using metaboanalyst 6.0 (http://metaboanalyst.ca.) server. To reduce the impact of noise and the variance of the variables, the data were scaled and transformed logarithmically. After these transformations, the median-centered and auto-scaled data underwent principal component analysis (PCA), supervised partial least squares discrimination analysis (PLS-DA), and orthogonal partial least squares discriminant analysis (OPLS-DA). Metabolites with a fold change (FC) value > 1.5 and a p-value less than 0.05 (as determined by repeated measures one-way ANOVA followed by Holm-Sidak’s multiple comparisons adjustment) were considered significantly different. These differential metabolites were mapped to biochemical pathways using metabolic enrichment and pathway analysis via the KEGG database (http://www.genome.jp/kegg/). Metabolites were categorized by the pathways in which they were involved or the functions they served. Enrichment analysis was further conducted to identify the biological pathways most impacted. All data were expressed as mean ± standard deviation (mean ± SEM). A value of p < 0.05 using Holm-Sidak’s multiple comparisons adjustment was considered significant. Group comparisons were conducted using a t-test and one-way ANOVA with the statistical analysis software GraphPad Prism 9.0 (La Jolla, CA).

### Metabolic and cellular bioenergetics analysis

The extracellular acidification rate (ECAR) and oxygen consumption rate (OCR) were assessed using the Seahorse XF Glycolysis Rate Assay Kit (Agilent Technologies, 103344-100) and the Seahorse XF Cell Mito Stress Test Kit (Agilent Technologies, 103015-100), respectively, following the manufacturer’s protocols using the Seahorse XFe 24 Analyzer (Agilent Technologies). A glycolytic stress test was performed to evaluate glycolytic parameters, including glycolysis, glycolytic capacity, and glycolytic reserve, using the Seahorse XF Glycolysis Stress Test Kit. One hour before the assay, the cell culture media were replaced with basal Seahorse media supplemented with glutamine, excluding glucose and pyruvate, to match culture conditions. The cells were incubated in a non-CO₂ 37 °C incubator for 1 hour before the initial measurement of “non-glycolytic acidification,” defined as the ECAR not attributed to glycolysis. Following this, 75 µl of glucose, oligomycin (an ATP synthase inhibitor), and 2-deoxyglucose (a competitive inhibitor of hexokinase, the first enzyme in glycolysis) were sequentially added to each well at working concentrations of 10 mM of glucose, 1 µM of oligomycin (Cayman, 11341), and 50 mM of 2-deoxyglucose (Cayman, 14325). These additions determined the ECAR associated with basal glycolysis, maximum glycolytic capacity, and confirmed that the initial ECAR was glycolysis dependent. Glycolysis was quantified as the glucose-induced increase in ECAR, calculated by subtracting non-glycolytic acidification from the highest ECAR measured after glucose addition. Maximum glycolytic capacity was determined as the difference between the highest ECAR during non-glycolytic acidification and the highest ECAR after oligomycin addition. Glycolytic reserve was calculated as the difference between ECAR after glucose and after oligomycin. All Seahorse assay data were normalized to cellular DNA content measured post-assay. Hoescht 33342 dye (Thermo Fisher Scientific, H1399) was added to each well at a 1:1,000 final concentration and incubated for 30 min at 37 °C with constant shaking. Fluorescence was recorded using a plate reader (excitation: 350 nm, emission: 461 nm). The mitochondrial stress test, or OCR analysis, was conducted using the Seahorse XF Cell Mito Stress Test Kit to evaluate mitochondrial function parameters, including basal respiration, ATP production-coupled respiration, maximal respiration, and spare capacity. One hour before the assay, the complete medium was replaced with basal Seahorse media supplemented with glucose (10 mM), glutamine (2 mM), and pyruvate (1 mM) to match the culture conditions. The cells were incubated in a non-CO₂ 37 °C incubator for 1 h before the initial measurement of the “basal respiration rate,” which represents the total mitochondrial respiration rate. Following the baseline measurement, oligomycin (2 µM; ATP synthase inhibitor), FCCP (1 µM; protonophore, Cayman, 15218-980), and a combination of rotenone (0.5 µM; NADH dehydrogenase inhibitor, Cayman, 13995-3822) and antimycin A (0.5 µM; cytochrome c reductase inhibitor, Sigma, 34799) were sequentially added to each well. These additions were used to measure ATP-coupled respiration, maximum respiration, and non-mitochondrial oxygen consumption, respectively. The ATP-coupled respiration was calculated as the difference between the basal OCR and the OCR after oligomycin addition, representing oxygen consumption linked to ATP production. Maximal respiration was determined as the difference between the OCR after FCCP addition and the lowest OCR measured post-oligomycin injection. Spare capacity (difference between maximal and basal respiration), indicative of the metabolic potential to counteract stress, was also calculated. Data were analysed using the Seahorse Wave Software v2.6.3.5 (Agilent Technologies).

### Disease score

To assess the disease severity following parasitic infection, a comprehensive set of parameters was evaluated, including the survivability of mice, weight loss, splenomegaly, and a variety of cellular and inflammatory assessments.

### Measurement of splenomegaly

Mice were weighed prior to and at regular intervals post-infection to monitor weight loss, an indicator of disease severity. The spleens were dissected post-euthanasia, weighed, and compared to the spleen weights of uninfected control mice. The relative spleen weight was calculated to quantify splenomegaly as a measure of systemic immune activation.

### Macrophage polarization

MΦ^β-cat-FL/FL^ macrophages from β-catenin^flox^ mice and CD11b-MΦ^β-cat–/–^macrophages from β-cat^ΔMΦ^ mice were differentiated as mentioned above and were seeded at a density of 1 × 10⁵ cells per well. The cells were infected with live RH strain parasites for 6 h. Following this, Brefeldin A (eBioscience, 00-4506-51) was added to the culture for an additional 4 h to inhibit protein secretion and enable intracellular cytokine detection. After the incubation, the cells were harvested and subjected to surface and intracellular staining using fluorophore-conjugated antibodies, as detailed in Table 1. Flow cytometry was subsequently performed to assess macrophage polarization (M1 vs M2 surface markers) and cytokine production, focusing on differences driven by the β-catenin expression profiles of the macrophages.

**Table 1:** The list of antibodies used for flow cytometry.

Sl. no	Manufacturer	Catalog number	Antibody/Reagent
1	BD Biosciences	569614	Ms CD3 BUV395 17 A2
2	BD Biosciences	570488	CD4 RB744 Rat Anti-Mouse
3	BD Biosciences	741342	Ms CD8b BUV563 H35-17.2
4	BD Biosciences	569181	Ms CD8a BUV496 53-6.7
5	BD Biosciences	564370	Ms CD25 BV421 3C7
6	BD Biosciences	560515	Ms NK1.1 Alexa 700 PK136
7	BD Biosciences	569089	T-bet RB780 O4-46
8	BD Biosciences	570645	IFN-γ RB705 Rat Anti-Mouse
9	BD Biosciences	514107	Alexa Fluor® 647 anti-mouse IL-9
10	BD Biosciences	571158	RORγt RY610 Mouse Anti-Mouse
11	BD Biosciences	570075	IL-17A BUV805 Rat Anti-Mouse
12	BD Biosciences	567922	ICOS (CD278) BV786 C398.4 A
13	BD Biosciences	566881	Ms FoxP3 PE 3G3
14	BD Biosciences	567633	GATA3 R718 L50-823
15	BD Biosciences	560699	Ms IL-4 PE-Cy7 11B11
16	BD Biosciences	570356	CD25 RB780 Rat Anti-Mouse (IL-2 Receptor α)
17	BD Biosciences	564331	Ms CD152 APC UC10-4F10-11
18	BD Biosciences	563553	CD11b BUV395 M1/70
19	BD Biosciences	744340	Ms F4/80-BV786
20	BD Biosciences	568973	Ms CD11c BV786 N418
21	BD Biosciences	560564	Ms IL-12 p40/p70 FITC C15.6
22	BD Biosciences	557644	Ms TNF PE-Cy7 MP6-XT22
23	BD Biosciences	565638	Ms LAP (TGF-β) BV421 TW7-16B4
24	BD Biosciences	564081	Ms IL-10 BV711 JES6-16E3
25	BD Biosciences	561363	Ms IL-6 Alexa 488 MP5-20F3
26	BD Biosciences	565317	Ms IL-23 p19 Alexa 647 N71-1183
27	BD Biosciences	567077	Ms IL-2 R718 JES7-5H4
28	BD Biosciences	554479	Ms IL-12 p40/p70 PE C15.6
29	BD Biosciences	567082	Ms IL-10 R718 JES6-16E3
30	BD Biosciences	566363	Ms IL-2 BV750 JES7-5H4
31	BD Biosciences	741927	Ms CD69 BUV805 H1.2F3
32	BD Biosciences	566646	Ms IRF4 PE
33	R&D System	FAB5891S-	Ms IL-2 R beta Alexa Fluor 750
34	Biolegend	514106	Ms APC IL-9 RM9A4
35	BD Biosciences	568345	Ms CD11b BUV805
36	BD Biosciences	561954	Ms CD80 FITC
37	Biolegend	141727	Ms CD206 BV711
38	BD Biosciences	562574	Transcription Factor Buffer Set
39	BD Biosciences	554714	Cytofix/Cytoperm Soln Kit
40	BD Biosciences	566385	Brilliant Stain Buffer Plus
41	BD Biosciences	560497	Anti Ms Ig CompBead Plus Set
42	BD Biosciences	552845	Rat/Ham Ig Kpa Comp Bead Set

### Macrophage migration assay

MΦ^β-cat-FL/FL^, and CD11b-MΦ^β-cat–/–^ macrophages (5 × 10⁴) were infected with freshly egressed tachyzoites at an MOI of 3. The cells were seeded onto gelatin-coated glass slides and incubated at 37 °C for various time points during infection. Subsequently, the macrophages were transferred onto transwell filters with 8 μm pore size (Corning, CLS4401) and incubated at 37 °C with 5% CO₂ for different infection durations. The migrated macrophages were quantified using a hemocytometer.

### In vitro cytokine and enzyme detection by ELISA

MΦ^β-cat-FL/FL^, and CD11b-MΦ^β-cat–/–^ macrophages were seeded (1 × 10^5^ per well) in 96-well plates and left to adhere overnight at 37 °C, 5% CO_2_. Cells were then infected with freshly egressed and filtered *T. gondii* parasites (3 MOI), and culture supernatants were collected at different time points of infection. CCL19 (MIP-3β) levels were measured by ELISA according to the manufacturer’s instructions (Thermo-Fischer Scientific, EMCCL19) in four independent experiments. Similarly, IL-1β levels were measured in the supernatants of macrophage or macrophage-T cell co-cultures at a 1:5 ratio using an ELISA assay (Thermo-Fischer Scientific, BMS6002-2), which quantitatively detects mouse IL-1β from cell culture medium according to the manufacturer’s instructions in four independent experiments. IL-1β from cell culture medium from THP-1 macrophages was detected using a human IL-1β ELISA kit (Thermo-Fischer Scientific, BMS224-2). Similarly, IFN-γ levels in the cell culture supernatant were measured using mouse- and human-specific ELISA kits (Thermo Fisher Scientific; KHC4021 and KMC4021C). HKII activity (Sigma, MAK091) and LDH-A levels (Sigma, MAK066) were measured in the cell culture medium using colorimetric enzyme activity assay kits, according to the manufacturer’s protocols, and absorbance was measured using a microplate reader.

### Macrophage-directed T-cells paradigm

To investigate β-catenin-dependent interaction between macrophages and T-cells, β-catenin^flox^ and β-cat^ΔMΦ^ mice were infected with the RH strain via the i.p. route. At 10 days post-infection, splenocytes were isolated and subjected to red blood cell (RBC) lysis. CD3⁺ T-cells were subsequently sorted using CD3ε-BUV395 (BD Biosciences, 569614) via flow cytometry. CD11b^+^F4/80+ gated macrophages, as mentioned above, were prepared from the two groups of mice: MΦβ-cat-FL/FL macrophages from β-catenin^flox^ mice and CD11b-MΦ^β-cat–/–^ macrophages from β-cat^ΔMΦ^ mice. Macrophages were incubated with complete soluble antigens (CSA) derived from the RH strain of *T. gondii* at a concentration of 40 μg for 6 h. Subsequently, purified CD3⁺ T cells, sorted from uninfected and infected splenocytes of both β-catenin^flox^ and β-cat^ΔMΦ^ mice, were co-cultured with these macrophages at a macrophage-to-T cell ratio of 1:5 for an additional 6 h. Specifically, MΦ^β-cat-FL/FL^ macrophages were co-cultured with purified CD3⁺ T cells isolated from β-catenin^flox^ mice, whereas CD11b-MΦ^β-cat–/–^ macrophages were co-cultured with purified CD3⁺ T cells derived from β-cat^ΔMΦ^ mice. Brefeldin A was then added to the culture for the final 4 h to facilitate intracellular cytokine detection. Subsequently, cells were harvested for surface and intracellular transcription factors and cytokines staining using fluorophore-conjugated antibodies. Flow cytometry was performed to analyze T-cell activation, cytokine production, and phenotypic changes of T-cells induced by macrophages with differing β-catenin expression profiles. This methodology provided insights into the role of β-catenin on macrophage-directed T-cell responses in *T. gondii* infection. The list of antibodies used for flowcytometry enlisted in Table 1.

## Supplementary information

Supplementary information

original Western data

Video S1A,B

Video S1C

## Data Availability

The whole transcriptome data generated in this study have been deposited in the NCBI-Sequence Read Archive (SRA) database and are publicly accessible with the accession number PRJNA1212584.

## Materials availability

Plasmids generated in this study are available from the lead contact upon request. This study did not generate new, unique reagents. Any additional information required to reanalyse the data reported in this paper is available from the lead contact upon request.
