# Supplementary material for: β-catenin-driven innate and metabolic reprograming in macrophages fuel T-cell-dependent inflammation in Toxoplasma gondii infection: implications for therapeutic intervention
Source: Cell Death Dis. 2026 Jun 13;17(1):568. doi: 10.1038/s41419-026-08953-1 (PMC13264635; doi:10.1038/s41419-026-08953-1)
Supplement: Supplementary file 1 — Supplementary information [file 41419_2026_8953_MOESM1_ESM.docx]

**SUPPLEMENTARY INFORMATION**

**
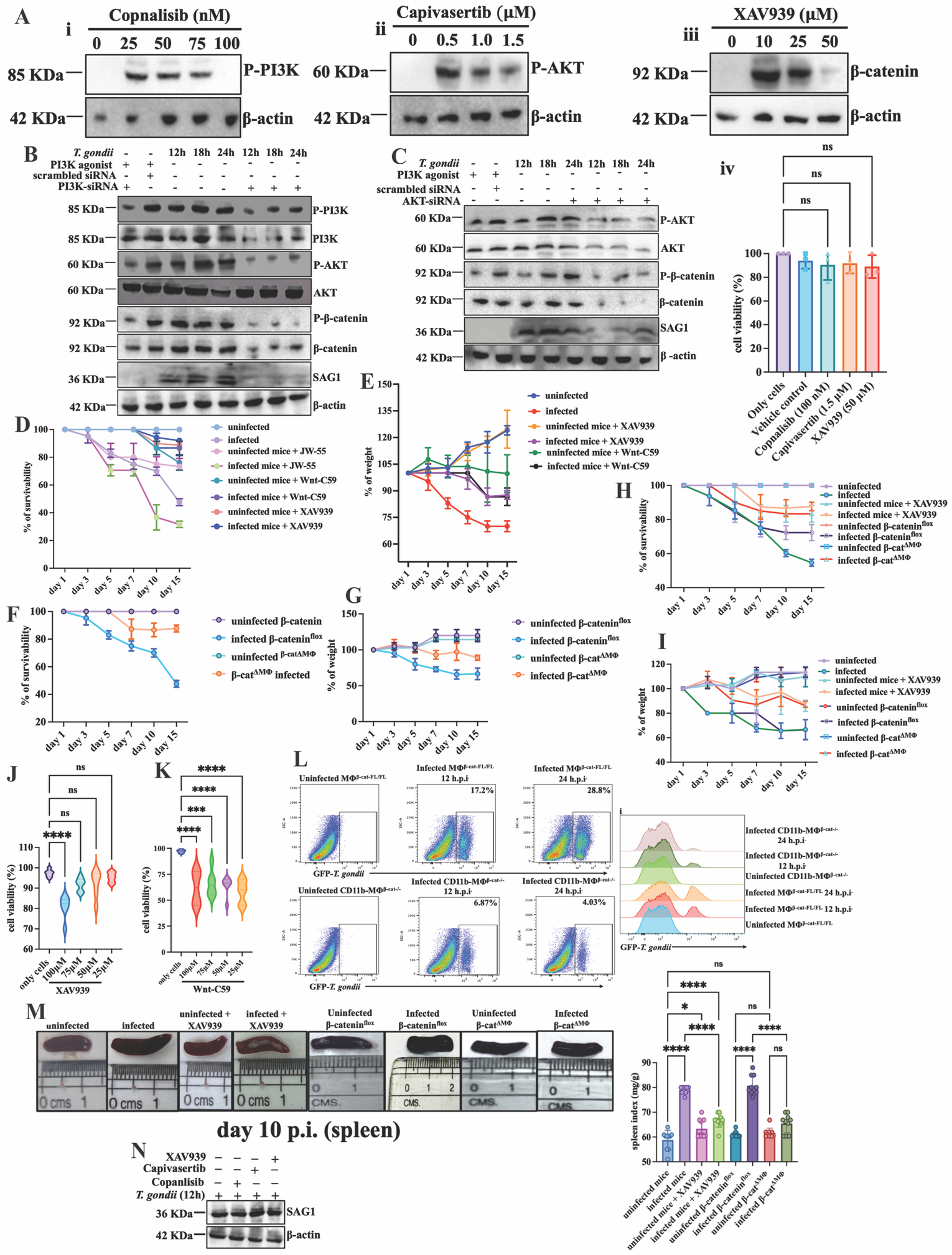
**

**Figure S1. β-catenin inhibition enhances survival, reduces weight loss in infected mice, and demonstrates minimal cytotoxicity in macrophages with inhibitors.** **(A)** Pharmacological inhibitors targeting **(i)** PI3K, **(ii)** AKT, and **(iii)** β-catenin were used to assess the functional dependency on these signaling pathways in a dose dependent manner. **(iv)** Cell viability was assessed following treatment with the effective dose of each inhibitor using CytoTox-Glo cytotoxicity assay kit (Promega) Data are shown as mean ± SEM from three independent experiments (n = 3), each performed in triplicate. **(B, C**) PI3K and AKT expression were silenced using specific siRNAs, with scrambled siRNA serving as a negative control. Different proteins expression levels were analyzed following parasite infection. Wild-type mice with different pharmacological inhibitors or β-catenin^flox^ mice or β-cat^ΔMΦ^ , and infected β-cat^ΔMΦ^  mice were infected with 50 type I parasites through i.p. to induce infection *in vivo*. Starting the day after infection, inhibitors of β-catenin signaling were administered intraperitoneally at a dose of 4 mg/kg body weight for 7 consecutive days. **(D, F)** Survival and **(E, G)** total body weight were monitored throughout the study. Each group consisted of 20 infected animals. Similarly, Wt mice, β-catenin^flox^ and β-cat^ΔMΦ^ mice were orally gavaged with 100 cysts of ME49 strain to induce infection *in vivo*. **(H)** Survival and **(I)** total body weight were monitored, with 20 infected animals per group. **(J, K)** WT-MΦ were seeded at a density of 1 × 10⁴ cells/well in 96-well plates and incubated for 12 h with increasing concentrations of XAV939 or Wnt-C59. Cytotoxicity was assessed using the CytoTox-Glo cytotoxicity assay (Promega), which measures protease activity associated with cytotoxicity. Relative luminescence values were recorded and normalized to untreated controls. Data are shown as mean ± SEM from six independent experiments (n = 6), each performed in triplicate. Statistical analysis was conducted using one-way ANOVA, with ****p < 0.0001, ***p < 0.001 indicating significance, and "ns" denoting no significance. **(L)** MΦ^β-cat-FL/FL^ and CD11b-MΦ^β-cat⁻/⁻^ macrophages were infected with GFP-*T. gondii* and percentage of GFP positive cells were analysed through flow cytometry and **(i)** the flow cytometry histogram illustrates GFP signal intensity as an indicator of *T. gondii* infection. **(M)** Spleen from i.p. infected mice were collected, as well as those organs were collected from infected β-catenin^flox^ and β-cat^ΔMΦ^ mice at 10 days post-infection. Spleen index was measured, and the average values were presented as bar graphs. Data shown are the mean ± SEM (n = 10) with statistical significance assessed using one-way ANOVA. Statistical significance is indicated as ****p<0.0001, *p<0.05 and "ns" indicating no significance. (N) Pharmacological inhibitors targeting PI3K, AKT, and β-catenin were used to assess their effect on parasites viability by immunoblotting to using SAG1 antibody.

**
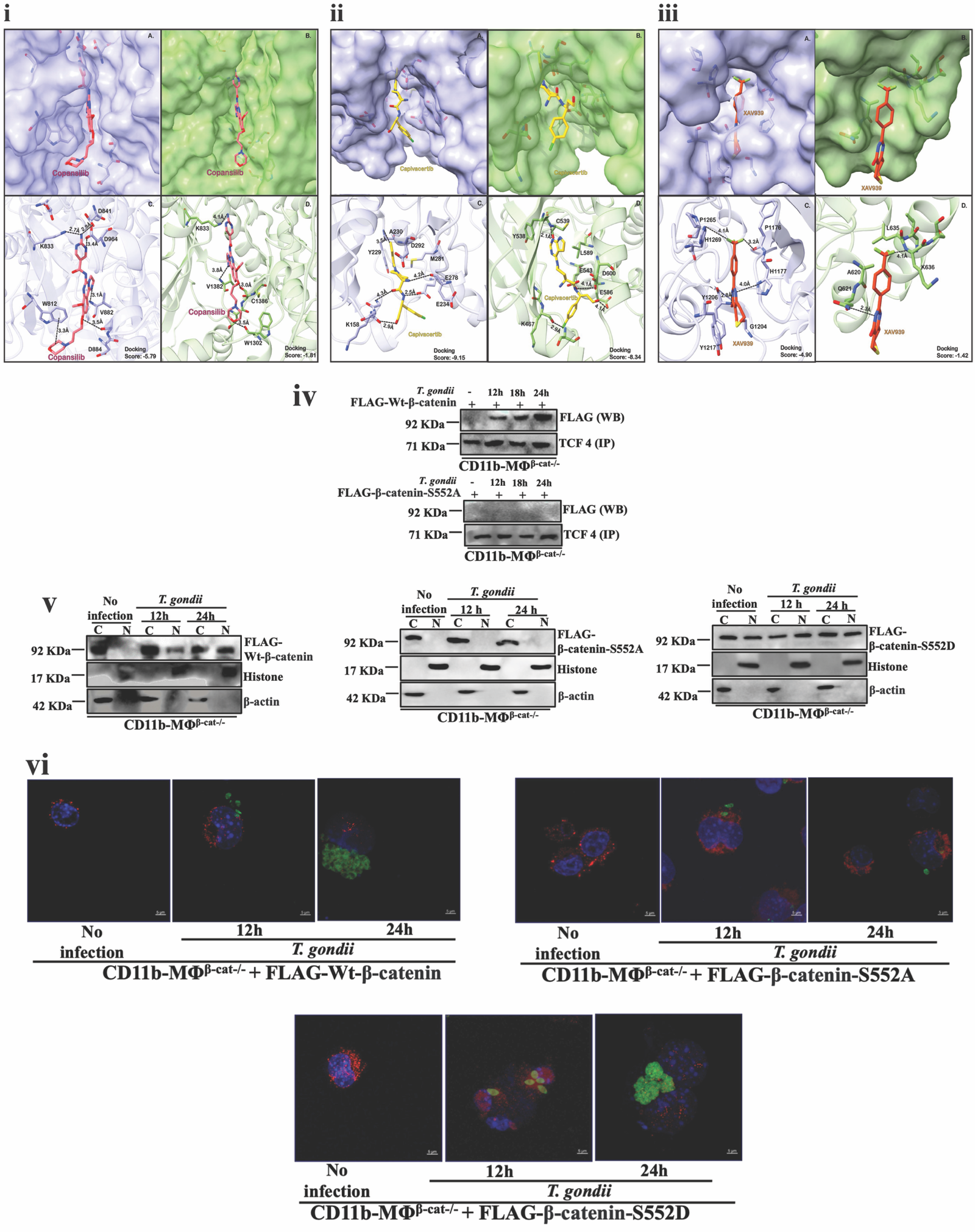
**

**Figure S2. Computational modelling, molecular docking, and interaction profiling of PI3K, AKT, and TNKS homologs from mouse and *T. gondii*.** i. Copansilib inhibitor comparative docking with the PI3K proteins of mice and *T. gondii* A. Inhibitor copansilib docked into the mouse PI3K protein's binding pocket, reveals a deep well enclosed binding cavity B. Copansilib’s surface representation within the T*. gondii* PI3K homolog, demonstrates a relatively more open and solvent-accessible binding site C. Copansilib interactions with important active site residues in mouse PI3K are shown in detail highlighting multiple interactions with the active site residues. D. Binding interactions of copansilib with the active site of *T. gondii* PI3K highlighting fewer stabilizing integrations with the active site residues. ii. Capivacertib inhibitor comparative docking with the AKT proteins of mice and *T. gondii* A. Capivacertib's docked into the mouse AKT protein's binding pocket, revealing a distinct and enclosed binding cavity B. Capivacertib's bound within the *T. gondii* AKT homolog, demonstrating a relatively more open and solvent-accessible binding site C. Capivacertib's interactions with important active site residues in mouse AKT are shown in detail highlighting the interactions with the active site residues. D. Binding interactions of capivacertib with the active site of *T. gondii* AKT. iii. Docking analysis of XAV939 with mouse and *T. gondii* tankyrase-1 (TNKS1) proteins. A. XAV939 (orange) surface representation docked into the mouse TNKS protein's binding pocket, revealing a deep well enclosed binding cavity. B. XAV939’s surface representation within the *T. gondii* TNKS homolog, demonstrating a relatively more open and solvent-accessible binding site. C. XAV939’s interactions with important active site residues in mouse TNKS are shown in detail in three dimensions. D. Binding interactions of XAV939’s with the active site of *T. gondii*. Very few stabilizing interactions with the active site residues were observed and the ligand was outside the active site. iv. Co-immunoprecipitation analysis showing interaction of Wt-β-catenin with TCF4 upon infection, which is lost in the S552A mutant. v. CD11b-MΦ^β-cat⁻/⁻^ macrophages were reconstituted with FLAG-tagged Wt-β-catenin, phosphorylation-deficient (S552A), or phospho-mimetic (S552D) mutants and infected with *T. gondii* where indicated. Immunoblot analysis of cytoplasmic and nuclear fractions demonstrating infection-induced nuclear translocation of WT β-catenin, cytoplasmic retention of the S552A mutant, and constitutive nuclear localization of the S552D mutant. vi. Confocal microscopy showing subcellular localization of FLAG-tagged β-catenin (red) and GFP-expressing parasites (green). Wt-β-catenin exhibits increased nuclear localization over time, S552A remains cytoplasmic, and S552D localizes to the nucleus even in uninfected cells. Nuclei are stained with DAPI (blue).

**
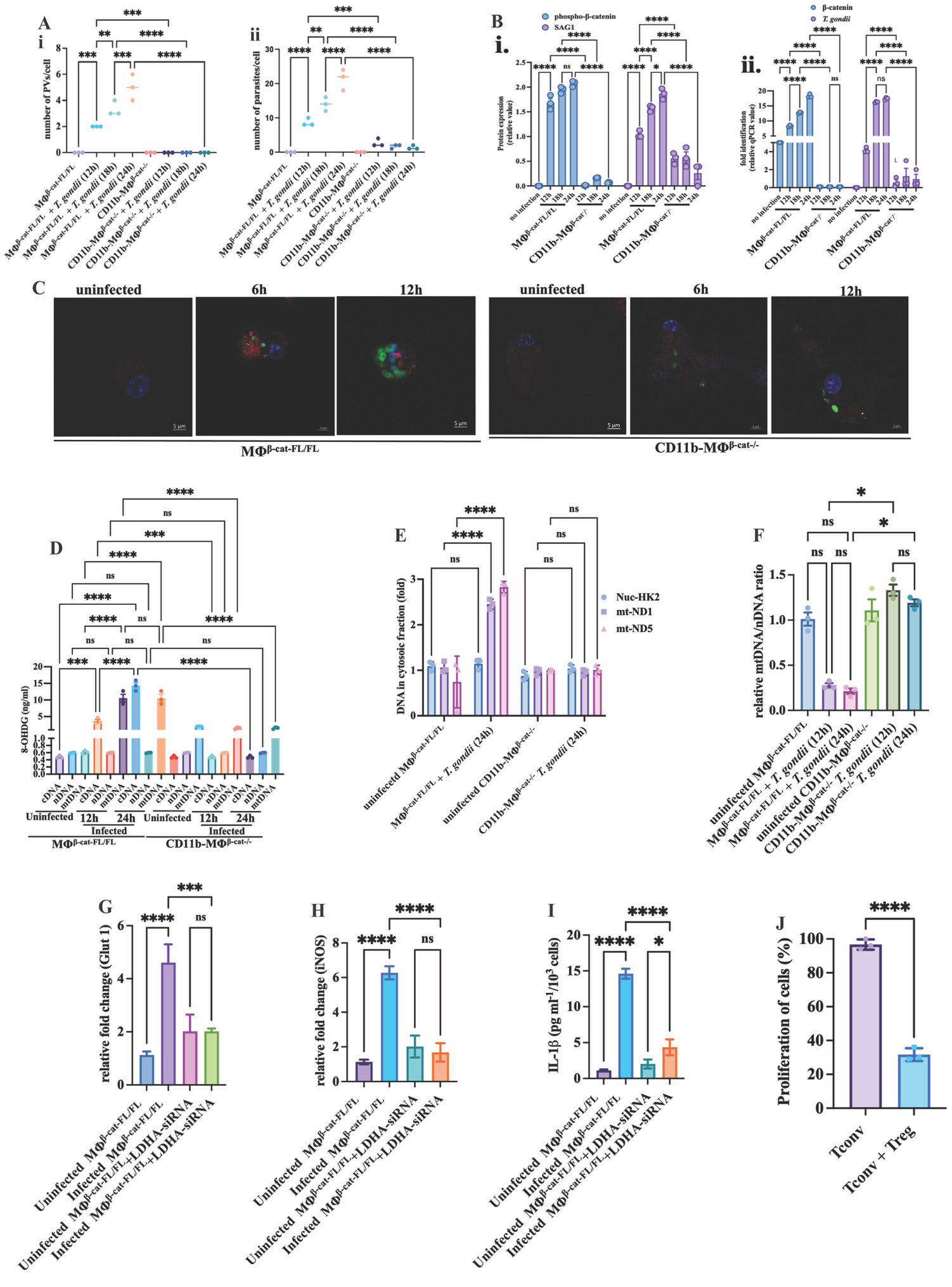
**

**Figure S3. β-catenin signaling in macrophages modulates oxidative DNA damage during T. gondii infection. (A)** **(i)** The number of PVs per cell, as well as **(ii)** the parasite numbers within infected cells were quantified and plotted as a bar graph based on data shown are the mean ± SEM (n = 3) with statistical significance assessed using two-way ANOVA. Statistical significance is indicated as ****p<0.0001, ***p<0.001, and **p<0.01. **(B) (i)** The average intensity of each band was plotted in bar diagram of three individual experiments after normalizing against total β-actin. Data shown are the mean ± SEM (n = 3) with statistical significance assessed using two-way ANOVA. Statistical significance is indicated as ****p<0.0001, and *p<0.05. **(ii)** The growth of *T. gondii* (*SAG1*) and expression of β-catenin (*CTNNB1*) in MΦ^β-cat-FL/FL^ and CD11b-MΦ^β-cat⁻/⁻^ macrophages were quantified by qPCR. Data shown are the mean ± SEM (n = 3) with statistical significance assessed using two-way ANOVA. Statistical significance is indicated as ****p<0.0001, and "ns" indicating no significance. **(C)** Confocal microscopy images showing 8-oxo-2'-deoxyguanosine (8-OHdG, red) staining in **MΦ-β-cat^FL/FL^** and **CD11b-MΦ^β-cat−/−^** macrophages at 0 (uninfected), 6 h, and 12 h post T. gondii infection. Nuclei are stained with DAPI (blue), and GFP-tagged *T. gondii* is shown in green. Scale bar: 5 μm. **(D**) **i.** Quantification of oxidative damage of cytoplasmic, nuclear and mitochondrial DNA by ELISA measuring 8-OHdG levels in DNA isolated from **MΦ-β-cat^FL/FL^** and **CD11b-MΦ^β-cat−/−^** macrophages at 12h.p.i. and 24h.p.i. Data represent mean ± SEM from three independent experiments. Statistical significance was determined using one-way ANOVA with ****p<0.0001, and "ns" indicating no significance. **(E)** Quantification of DNA leaked into cytoplasm from either nucleus (*HK2* gene) or mitochondria (*ND1*, *ND5* gene) by qPCR analysis was done from **MΦ-β-cat^FL/FL^** and **CD11b-MΦ^β-cat−/−^** macrophages at 24h.p.i. Data represent mean ± SEM from three independent experiments. Statistical significance was determined using one-way ANOVA with ****p<0.0001, and "ns" indicating no significance. **(F)** Relative mitochondrial DNA copy number (mtDNAcn) was measured in experimental groups. mtDNAcn was determined by qPCR targeting *MT-ND1* and normalized to the nuclear gene *HK2*. Data represents mean ± SEM from triplicate reactions (n=3). Statistical analysis was conducted using one-way ANOVA, with *p<0.01, and "ns" indicating no significance. **(G-I)** LDHA expression was silenced in **MΦ-β-cat^FL/FL^ by siRNA** and then **MΦ-β-cat^FL/FL^ or LDHA-siRNA treated MΦ-β-cat^FL/FL^ cells were infected with parasitise for 12 hours and the expression of Glut1, iNOS, and IL-1β expression** were quantified by qRT-PCR. Data shown are the mean ± SEM (n = 3) with statistical significance assessed using two-way ANOVA. Statistical significance is indicated as ****p<0.0001, ***p<0.001, and *p<0.05, and "ns" indicating no significance. **(J)** Bar diagram illustrating the proliferative capacity of T-cell subsets under *ex vivo* stimulation. CD4⁺CD25⁻FoxP3⁻ conventional T-cells (Tconv) from uninfected β-catenin^flox^ mice were stimulated for 72 hours with plate-bound anti-CD3 and anti-CD28 antibodies. In contrast, Tconv co-cultured with CD4⁺CD25⁺FoxP3⁺ Treg cells isolated from T. gondii-infected **β-cat^ΔMΦ^**  mice displayed significantly reduced proliferation (~35%). Proliferation was quantified using a CCK-8 colorimetric assay at 450 nm. Data are presented as mean ± SEM from three independent experiments. Statistical significance was assessed using an unpaired t-test, with ****p < 0.0001 indicating a highly significant difference.

**
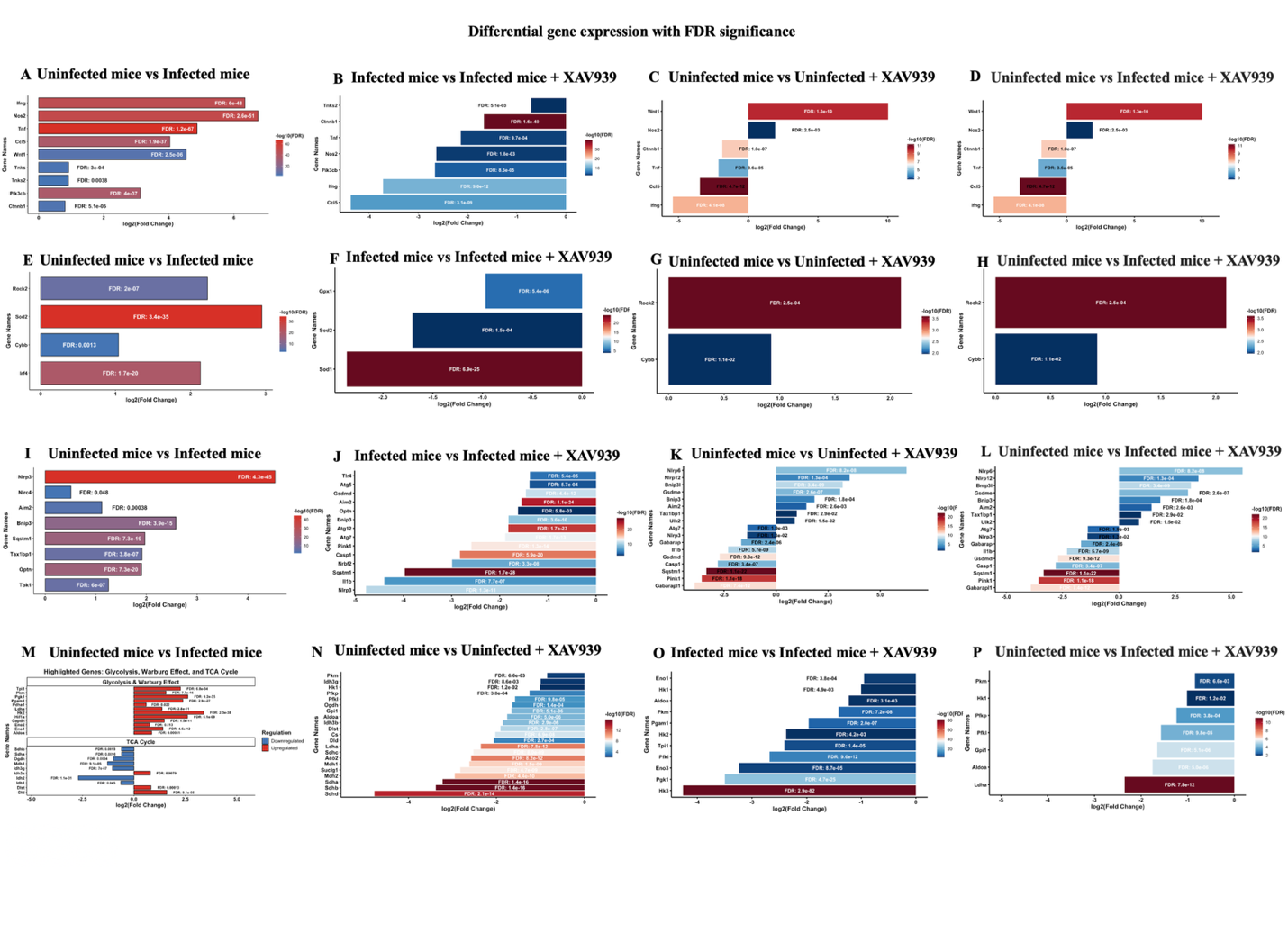
**

**Figure S4. Differential gene expression (DGE) and pathway enrichment analysis following whole transcriptome profiling.** Differential gene expression (DGE) analysis of total RNA isolated from splenocytes was conducted using DESeq2 to identify differentially expressed genes (DEGs) across the following comparisons: **(A, E, I, M)** uninfected vs. infected mice, **(B, F, J, N)** infected mice vs. infected mice treated with XAV939, **(C, G, K, O)** uninfected mice vs. uninfected mice treated with XAV939, and **(D, H, L, P)** uninfected mice vs. infected mice treated with XAV939. Bar plots were generated to visualize DEGs, with fold changes (log2) represented on the X-axis and gene names presented on the Y-axis. The false discovery rate (FDR) was represented as**-log10(p-adjusted value)** to highlight the significance of each DEG. Abbreviations*: Ifng*, Interferon-γ; *Nos2*, nitric oxide synthase 2; *Tnf*; tumor necrosis factor-α; *Ccl5*, CC-chemokine ligand 5; *Wnt1*, wingless-type MMTV integration site family, member 1; *Tnks*, tankyrase 1; *Tnks2*, tankyrase 2; *Pi3kcb*, phosphatidylinositol-3 kinase; *Ctnnb1*, β-catenin; Rock2, ho-associated, coiled-coil-containing protein kinase 2; Sod2, superoxide dismutase 2; cybb, cytochrome b-245, beta chain (NADPH oxidase); *Irf4*, Interferon regulatory factor 4; *Nlrp6*/*Nlrp12*/*Nlrp3*, NOD‐like receptor family pyrin domain containing-6/12/3; *Bnip3l*, BCL2/adenovirus E1B ligand; *Gsdme*, Gasdermin E; *Bnip3*, BCL2/adenovirus E1B; *Aim2*, absent in Melanoma 2; *Taxbp1*, Tax1-binding protein 1; *Ulk2*, Unc-51-like autophagy activating kinase 2; *Atg7*, autophagy related 7; *Gabarap*, gamma-aminobutyric acid receptor-associated protein; *Il1b*, interleukin-1-β; *Gsdmd*, Gasdermin D; *Casp1*, caspase 1; *Sqstm1*, sequestosome 1; *Pink1*, PTEN-induced kinase 1; *Pkm*, pyruvate kinase M; *Tpi1*, triosephosphate isomerase 1; *Pgk1*, phosphoglycerate kinase 1; *Pgam1*, phosphoglycerate mutase 1; *Pdha1*, pyruvate dehydrogenase-A1; *Ldha*, lactate dehydrogenase-A; *Hk2*, hexokinase 2; *Hif1a*, hypoxia-inducible factor-1; *Gapdg*, glyceraldehyde-3-phosphate dehydrogenase; *Eno2*, enolase 2; *Aldoa*, fructose-bisphosphate aldolase A; *Sdhb*, succinate dehydrogenase B; *Ogdh*, 2-oxoglutarate dehydrogenase; *Mdh1*, malate dehydrogenase 1; *Idh3g*, isocitrate dehydrogenase; *Idh3a*, isocitrate dehydrogenase (NAD(+)) 3 catalytic subunit alpha; *Idh1/2*, NADP(+)-dependent isocitrate dehydrogenase-1/2; *Dlst*, Dihydrolipoamide S-Succinyltransferase; *Dld*, dihydrolipoamide dehydrogenase.

**
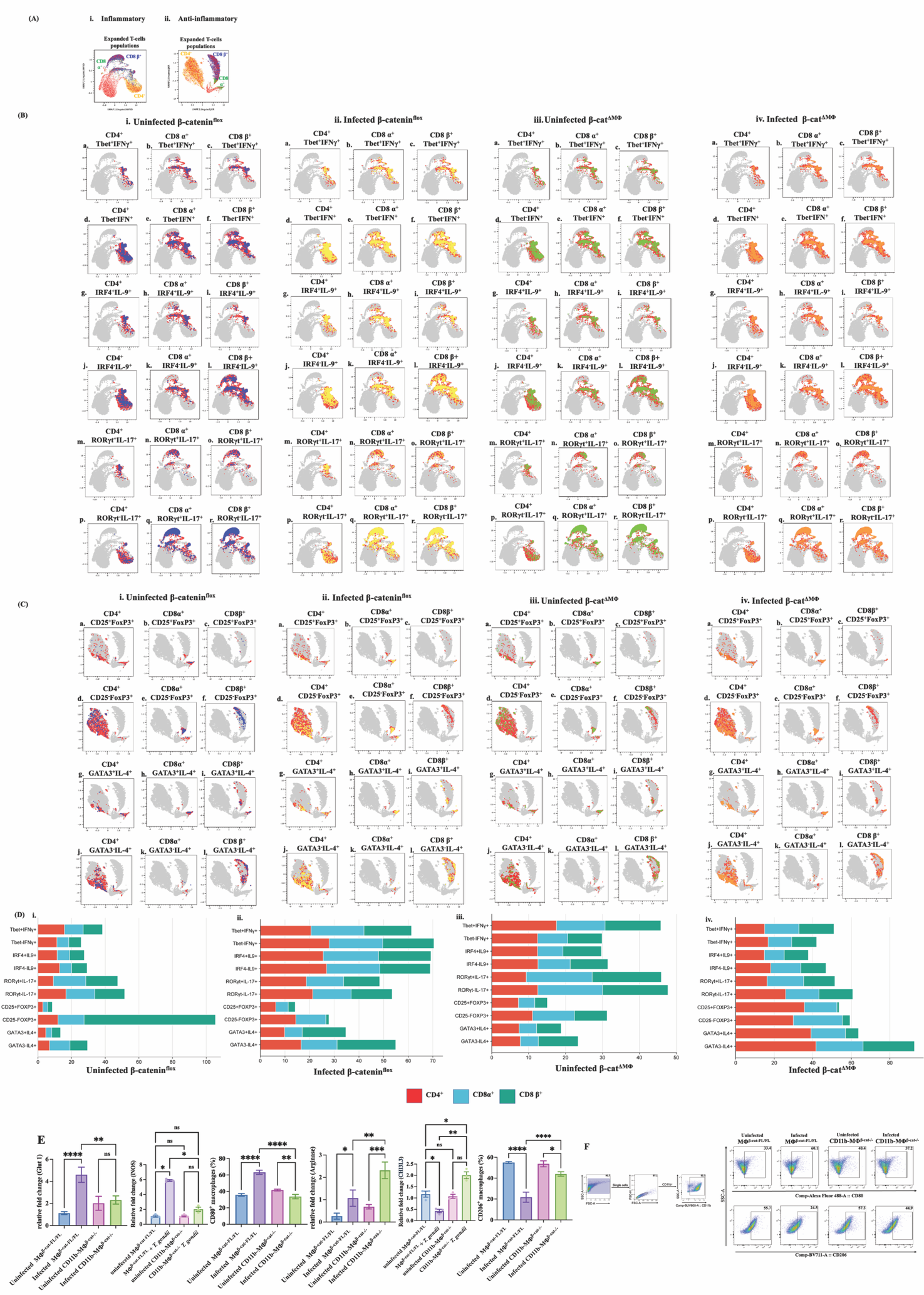
**

**Figure S5. β-catenin in macrophages shapes T-cells differentiation and cytokine expression during infection. (A)** UMAP visualization of expanded T-cell subsets from mice, displaying CD3⁺, CD4⁺, CD8α⁺, and CD8β⁺ populations. **(B)** Feature plots illustrate protein level expression of inflammatory cytokines and their corresponding transcription factors across CD4⁺, CD8α⁺, and CD8β⁺ T-cells from four different experimental groups (i-iv). Specifically, the plots depict: (a-c) Tbet^+^IFNγ^+^; (d-f) Tbet^-^IFNγ^+^; (g-i) IRF4^+^IL-9^+^; (j^-^l) IRF4^-^IL-9^+^, (m-o) RORγt^+^IL-17^+^; (p-r) RORγt^-^IL-17^+^. Representative intracellular cytokines IFNγ, IL-9, and IL-17, along with transcription factors T-bet, IRF4, and RORγt, were evaluated in expanded T-cell subsets to assess their differentiation patterns.  **(C)** Feature plots present the expression of regulatory cytokines and their transcription factors across CD4⁺, CD8α⁺, and CD8β⁺ T-cells from different experimental groups (i-iv). Specifically, the plots depict: (a-c) CD25⁺FoxP3⁺, (d-f) CD25⁻FoxP3⁺, (g-i) GATA3⁺IL-4⁺, and (j-l) GATA3⁻IL-4⁺. Representative intracellular cytokine IL-4 and transcription factors FoxP3 and GATA3 were analyzed to determine the differentiation patterns of expanded T-cell subsets.  **(D)** A stacked bar plot represents cytokine and transcription factor expression across three T-cell subtypes (CD4⁺, CD8α⁺, CD8β⁺) in different groups of mice (i-iv). The X-axis denotes the percentage expression of various markers within the T-cell subtypes, while the Y-axis lists cytokine marker subsets, including inflammatory markers (T-bet⁺IFNγ⁺, T-bet⁻IFNγ⁺, IRF4⁺IL-9⁺, IRF4⁻IL-9⁺, RORγt⁺IL-17⁺, RORγt⁻IL-17⁺) and anti-inflammatory markers (CD25⁺FoxP3⁺, CD25⁻FoxP3⁺, GATA3⁺IL-4⁺, GATA3⁻IL-4⁺). Different T-cell subtypes are color-coded: CD4⁺ (Red), CD8α⁺ (Blue), and CD8β+ (Green). **(E)** qRT-PCR analysis of **Glut1, iNOS, arginase, and CHI3L3 (Ym1)** expression and FACS analysis of **CD80** and **CD206** surface markers in uninfected and infected **MΦ^β-catFL/FL^** and **CD11b-MΦ^β-cat−/−^** macrophages at 12 h post-T. gondii infection. **(F)** Representative flow cytometry plots showing CD80⁺ and CD206⁺ macrophage populations under the same conditions. Data are presented as mean ± SEM (n = 3), and statistical significance was determined using two-way ANOVA. ****p < 0.0001, ***p < 0.001, **p < 0.01, *p < 0.05, ns = not significant.


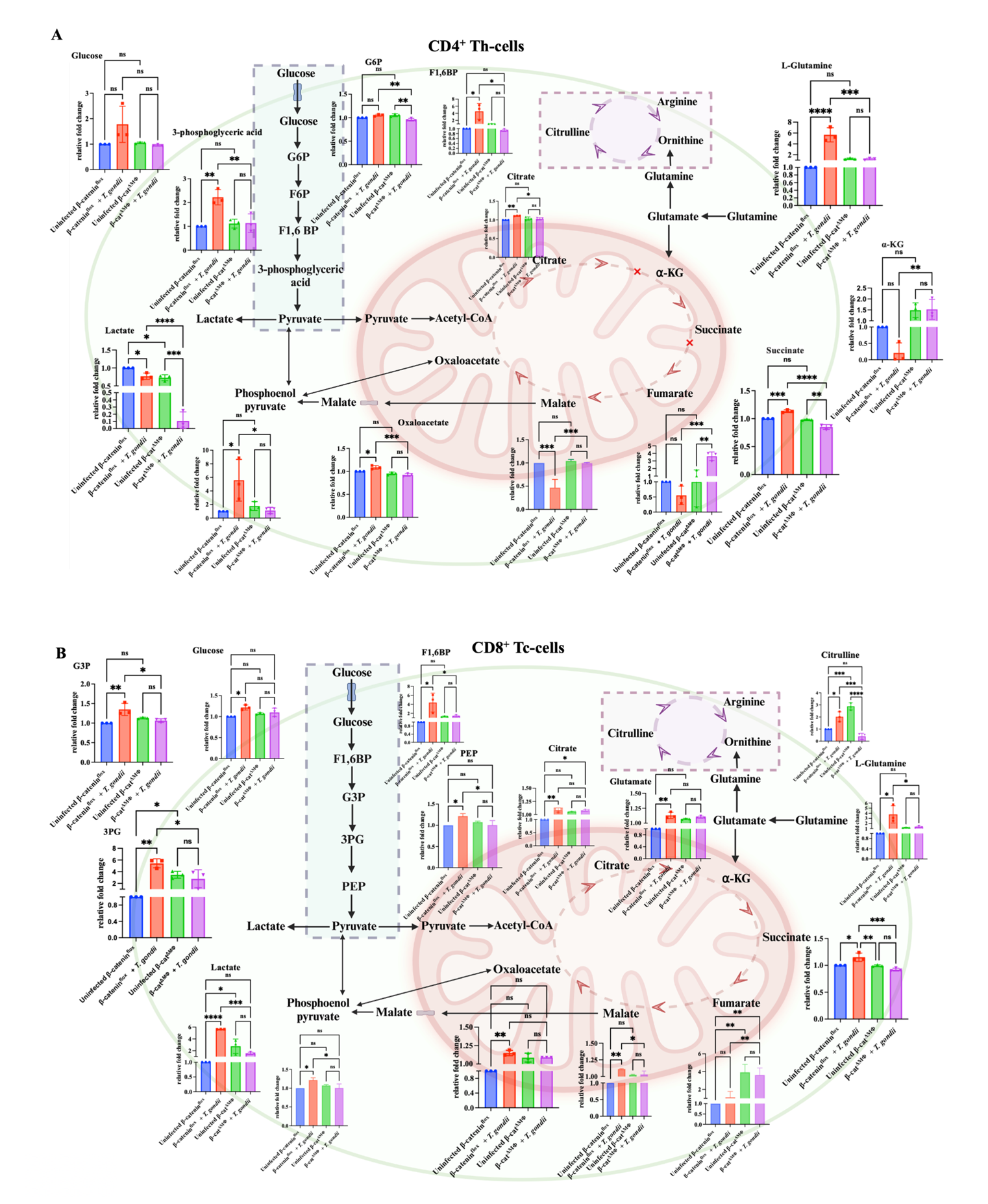


**Figure S6.** **β-catenin regulates the metabolic profile of T-cell subsets, influencing their polarization in response to parasitic infection.** Macrophages (BMDM) were differentiated from uninfected β-catenin^flox^ or β-cat^ΔMΦ^ mice and incubated with CSA for 6 hours. Subsequently, macrophages and T-cells were co-cultured under similar sources and conditions. For example, when macrophages were derived from β-catenin^flox^ mice, the splenic CD3⁺ T-cells were also obtained from β-catenin^flox^ mice, either uninfected or infected. After 6 hours of co-culture, total cell population was used for sorting using **(A)** CD4^+^ and **(B)** CD8^+^ surface markers of T-cells and analyzed for metabolites via LC-MS/MS. The relative fold changes of key glycolysis and TCA cycle metabolites were measured. The bar diagrams represent the average data from three experiments conducted within the same batch for each metabolite. Data shown are the mean ± SEM (n = 3) with statistical significance assessed using one-way ANOVA. Statistical significance is indicated as ****p<0.0001, ***p<0.001, **p<0.01, *p<0.05, and "ns" indicating no significance. Abbreviations: G6P, glucose-6-phosphate; F6P, fructose-6-phosphate; F1,6BP, fructose 1,6-bisphosphate; 3-Phosphoglyceric acid; PEP, Phosphoenolpyruvate; α-KG, α-ketoglutarate.

**
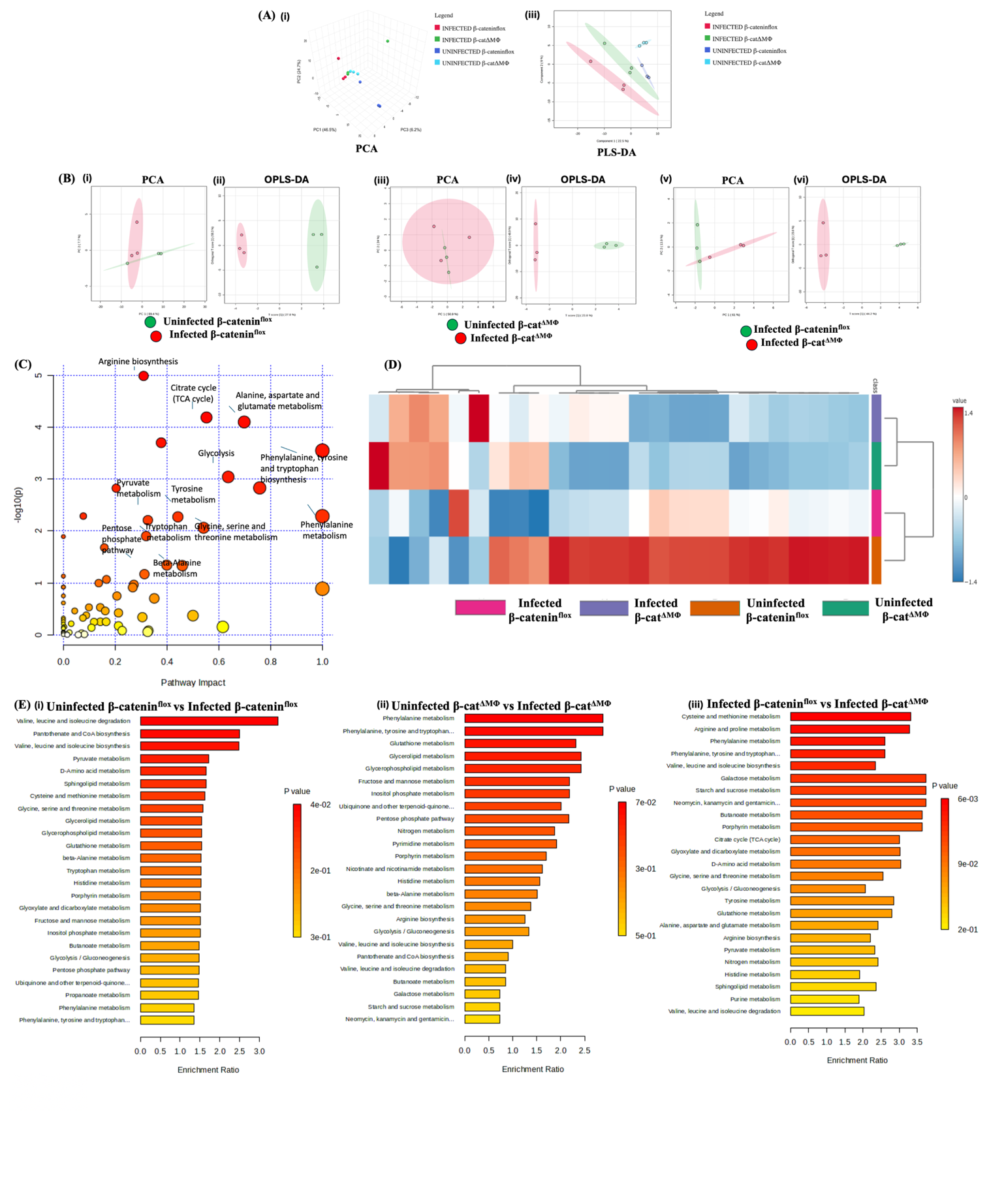
**

**Figure S7. Baseline metabolic profile CD4^+^ T-cells. (A)** Principal component analysis (i) and PLS-DA (ii) score plots show variability among the metabolic profile CD4^+^ T-cells from uninfected β-catenin^flox^ (blue), infected β-catenin^flox^ (red), uninfected β-cat^ΔMΦ^ (light blue) and infected β-cat^ΔMΦ^ (green) groups of mice at baseline. **(B)** PCA and orthogonal partial least squares discriminant analysis (OPLS-DA) are used to compare metabolite differences in CD4^+^ T-cells within the groups (i-vi). The PLS-DA analysis of the metabolite profiles revealed significant differences between all the groups, suggesting that the PLS-DA model effectively distinguishes between the groups, demonstrating high discrimination and prediction rates (*p* < 0.05). **(C)** Metabolic pathway analysis bubble diagram. Metabolic pathway analysis used the MetaboAnalyst 6.0 software; the circle size based on pathway impact value; color based on P value (p<0.05) (n = 3).  **(D)** The hierarchical clustering heatmap reveals that the overall metabolic profile of CD4^+^ T-cells from uninfected β-catenin^flox^ mice is markedly distinct from those of infected β-catenin^flox^, uninfected β-cat^ΔMΦ^ , and infected β-cat^ΔMΦ^  mice. A large number of metabolites show higher expression in uninfected β-catenin^flox^ derived CD4^+^ T-cells compared to other groups. The expression is given as follows: red, upregulated; blue, downregulated; and white, unregulated (p <0.05, Kruskal-Wallis test). **(E)** Pathway enrichment analysis: The bar plot represents the pathways up-regulated and down-regulated in CD4^+^ T-cells of Uninfected β-catenin^flox^ vs Infected β-catenin^flox^ (i), Uninfected β-cat^ΔMΦ^ vs Infected β-cat^ΔMΦ^ (ii) and Infected β-catenin^flox^ vs Infected β-cat^ΔMΦ^ (iii) respectively (*p* < .05; FC > 1.5). The *x*-axis represents the enrichment ratio and *y*-axis represents the pathways.

**
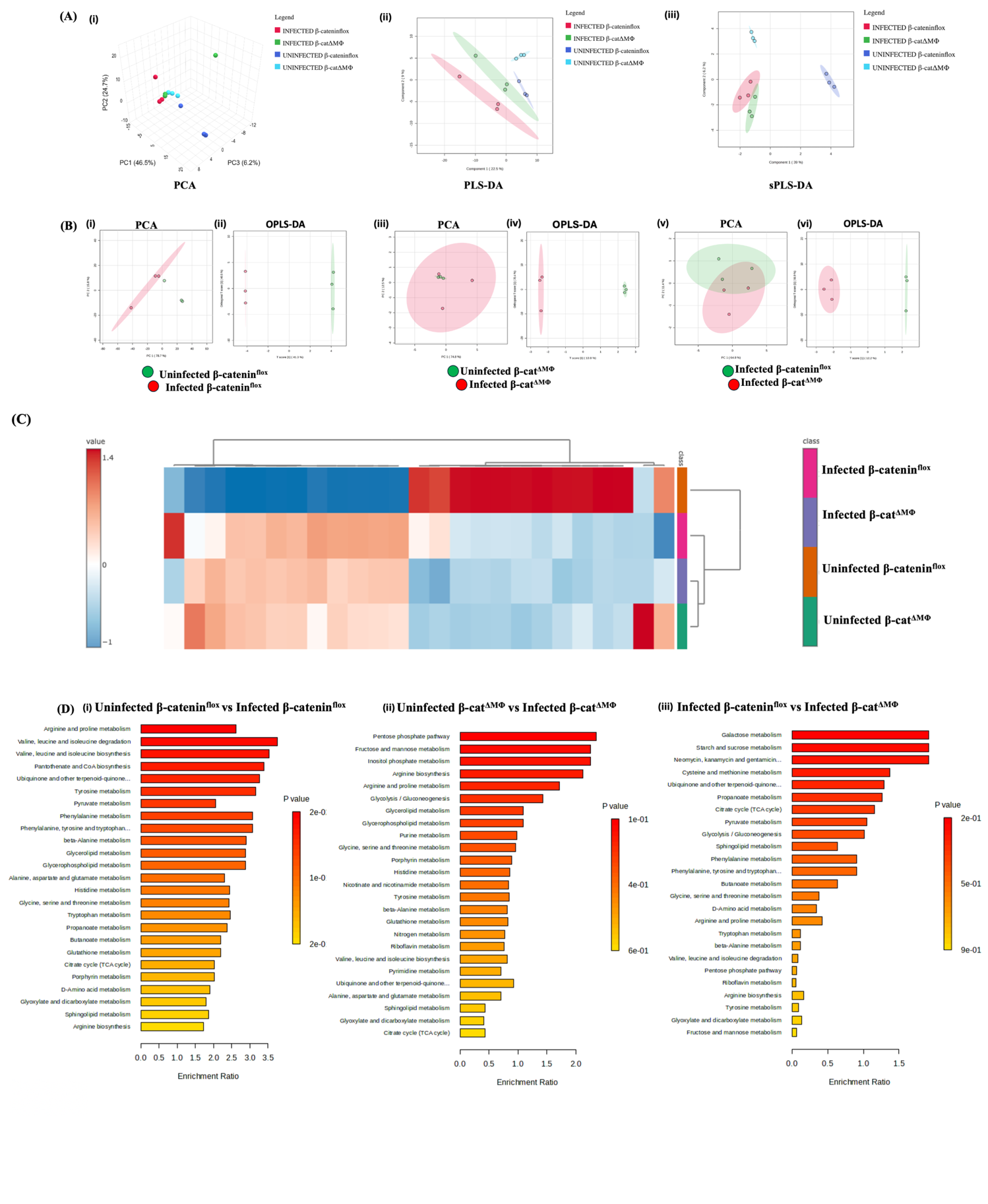
**

**Figure S8. Baseline metabolic profile of CD8^+^ T-cells. (A)** Score plot of the metabolic profile variability of CD8^+^ T-cells from uninfected β-catenin^flox^, infected β-catenin^flox^, uninfected β-cat^ΔMΦ^, infected β-cat^ΔMΦ^ groups of mice using 3D-PCA (i), PLS-DA (ii) and sPLS-DA (iii) models. **(B)** PCA and orthogonal partial least squares discriminant analysis (OPLS-DA) are employed to analyze metabolite differences in CD8^+^ T cells within the groups (i-vi). **(C)** A heatmap illustrating the overall metabolite profile changes across CD8^+^ T cells from different groups of mice. The expression is given as follows: red, upregulated; blue, downregulated; and white, unregulated (p <0.05, Kruskal-Wallis test). **(D)** Pathway enrichment analysis of upregulated and downregulated biological pathways in CD8^+^ T cells from Uninfected β-catenin^flox^ vs Infected β-catenin^flox^, Uninfected β-cat^ΔMΦ^ vs Infected β-cat^ΔMΦ^ and Infected β-catenin^flox^ vs Infected β-cat^ΔMΦ^ respectively (*p* < .05; FC > 1.5). The *x*-axis represents the enrichment ratio and *y*-axis represents the pathways.

**
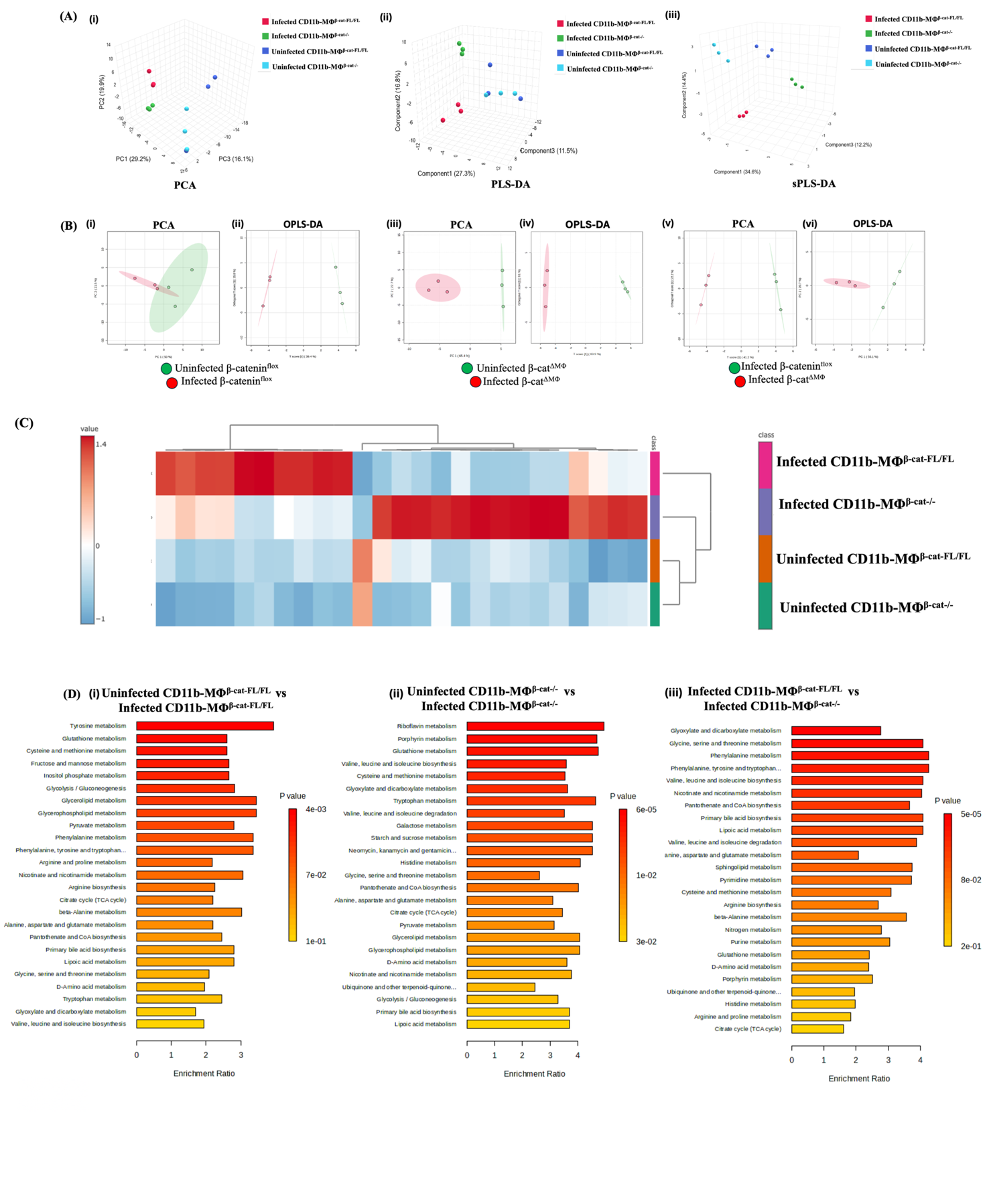
**

**Figure S9. Baseline metabolic profile of CD11b^+^F4/80^+^ macrophages. (A)** Score plot of the metabolic profile variability of CD11b^+^F4/80^+^ macrophages from uninfected CD11b-MΦ^β-cat-FL/FL^, infected CD11b-MΦ^β-cat-FL/FL^, uninfected CD11b-MΦ^β-cat-/-^ , infected CD11b-MΦ^β-cat-/-^ groups of mice using 3D-PCA (i), PLS-DA (ii) and sPLS-DA (iii) models. **(B)** PCA and orthogonal partial least squares discriminant analysis (OPLS-DA) employed to analyze metabolite differences in CD11b^+^F4/80^+^ macrophages within the groups (i-vi). **(C)** A heatmap illustrating the overall metabolite profile changes across CD11b^+^F4/80^+^ macrophages from different groups of mice. The expression is given as follows: red, upregulated; blue, downregulated; and white, unregulated (p <0.05, Kruskal-Wallis test). **(D)** Pathway enrichment analysis of upregulated and downregulated biological pathways in CD11b^+^F4/80^+^ macrophages from uninfected CD11b-MΦ^β-cat-FL/FL^ vs infected CD11b-MΦ^β-cat-FL/FL^, uninfected CD11b-MΦ^β-cat-/-^ vs infected CD11b-MΦ^β-cat-/-^ and infected CD11b-MΦ^β-cat-FL/FL^ vs infected CD11b-MΦ^β-cat-/-^ respectively (*p* < .05; FC > 1.5). The *x*-axis represents the enrichment ratio and *y*-axis represents the pathways.

**Video S1. Dynamic imaging of GFP-RH parasite infection in macrophages.** Live imaging of **(A)** **MΦ^β-cat-FL/FL^** and **(B)** **CD11b-MΦ^β-cat⁻/⁻^** macrophages infected with GFP-RH parasites at a **1:3 MOI** was conducted using confocal microscopy. To understand the infection dynamics, live images were captured from **1 h.p.i. to 30 h.p.i. (C)** MΦ^β-cat-FL/FL^ cells were infected with the GFP-RH strain of T. gondii and, at specified time points, stained with MitoTracker (red) for mitochondria and DAPI (blue) for nuclei. Confocal microscopy was used to capture the dynamics of infection, spanning from **24 h.p.i. to 30 h.p.i**.
