# Supplementary material for: β-catenin-driven innate and metabolic reprograming in macrophages fuel T-cell-dependent inflammation in Toxoplasma gondii infection: implications for therapeutic intervention
Source: Cell Death Dis. 2026 Jun 13;17(1):568. doi: 10.1038/s41419-026-08953-1 (PMC13264635; doi:10.1038/s41419-026-08953-1)

Figure 1

A

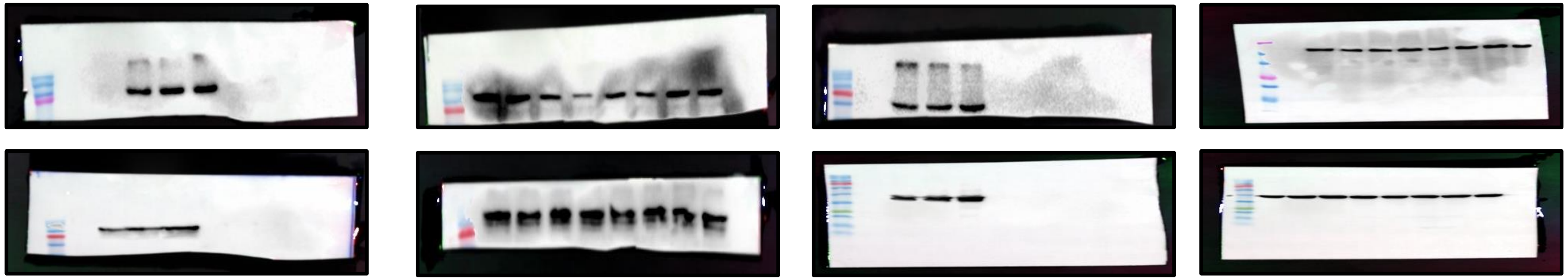

B

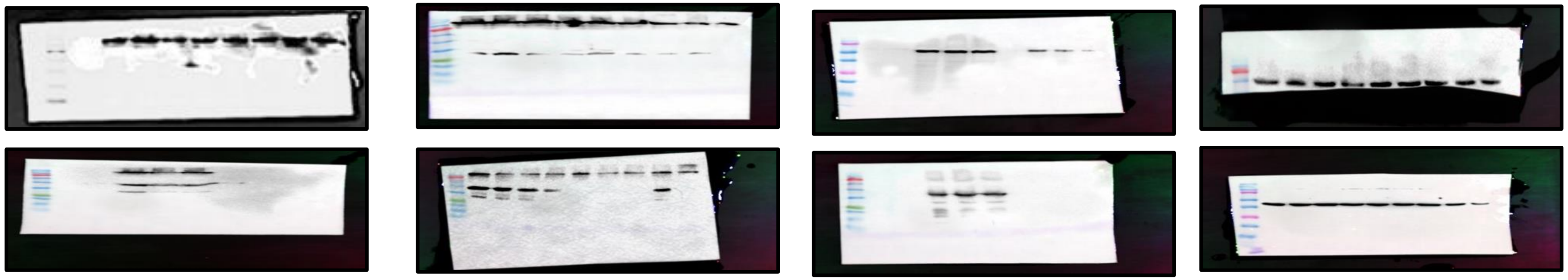

C

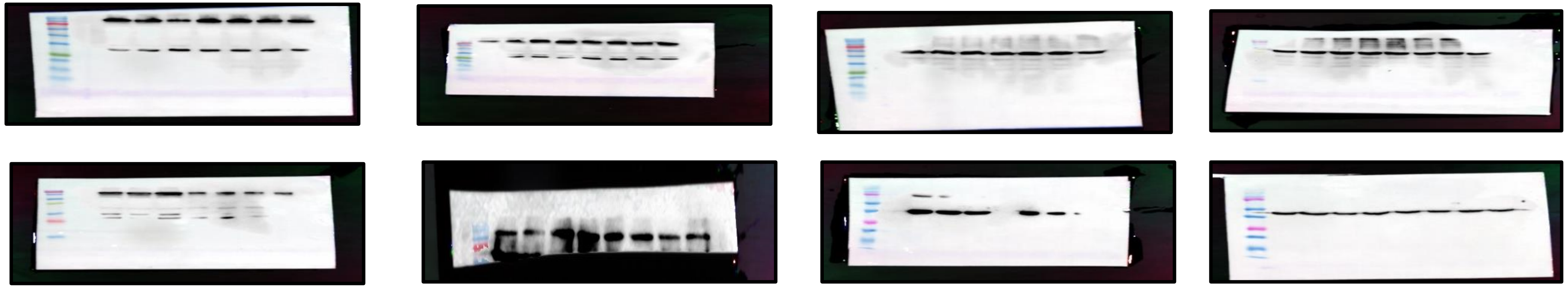

Figure 2

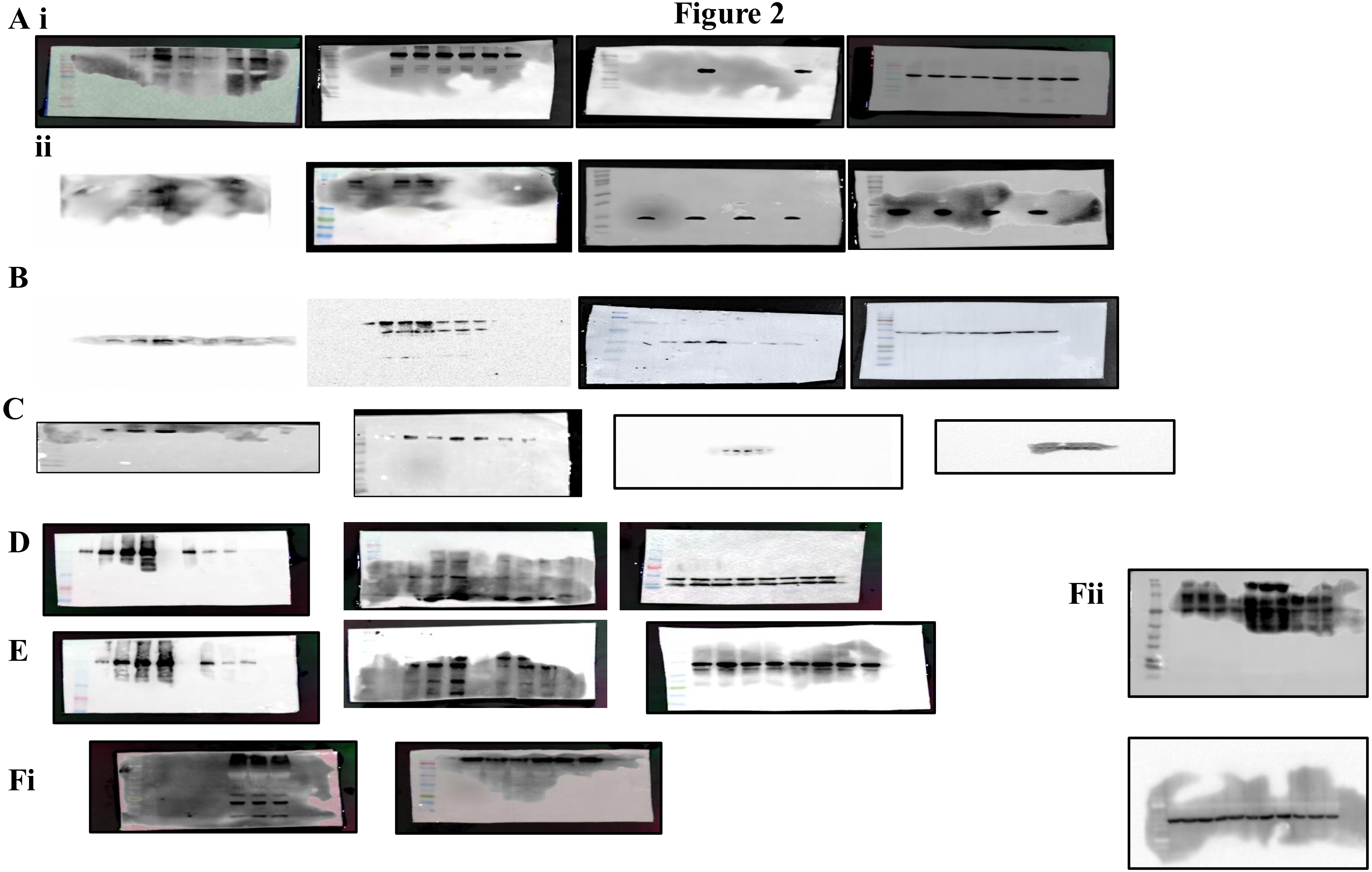

Figure 2H

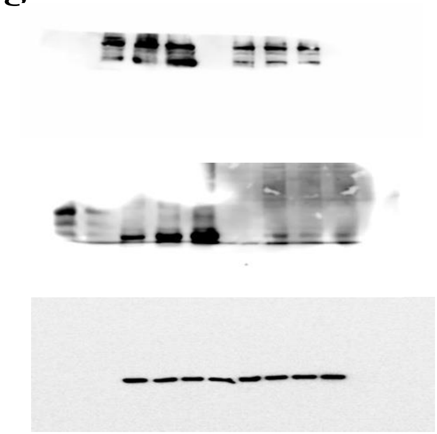

Figure 2L

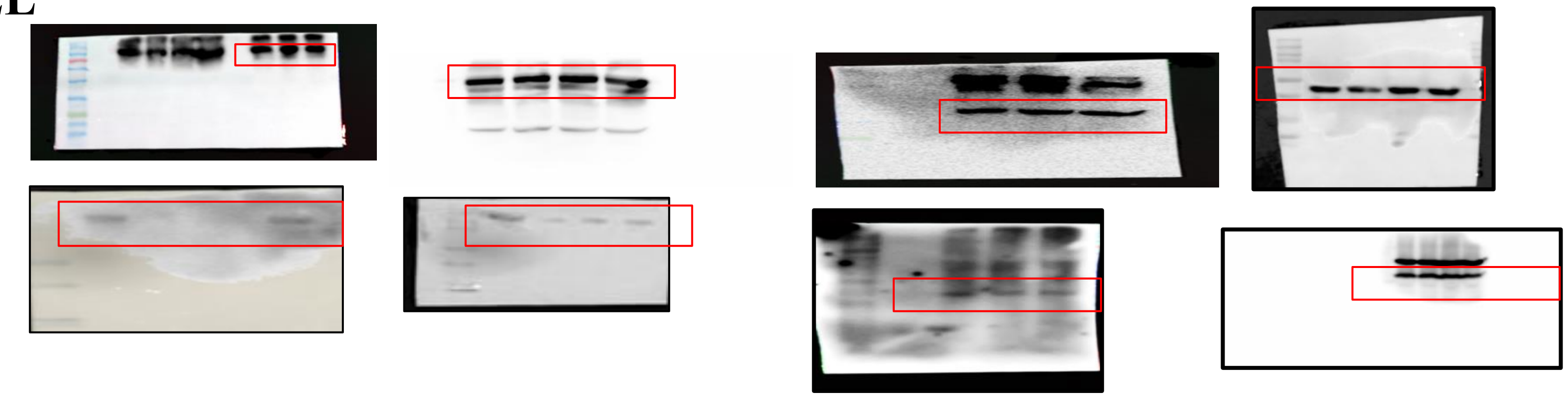

Figure 3

F

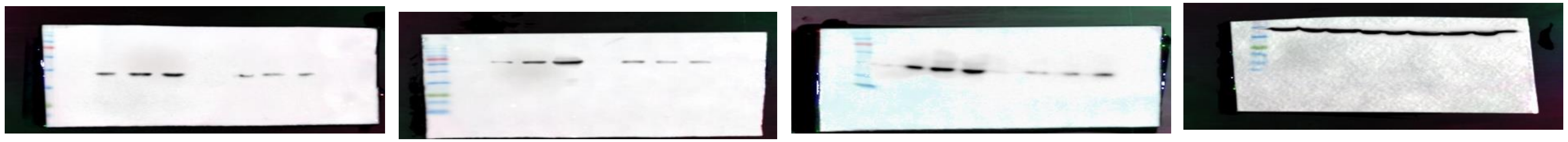

G

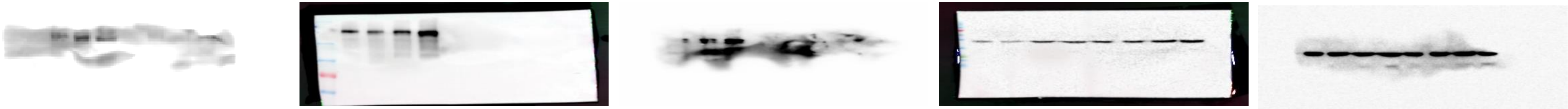

H

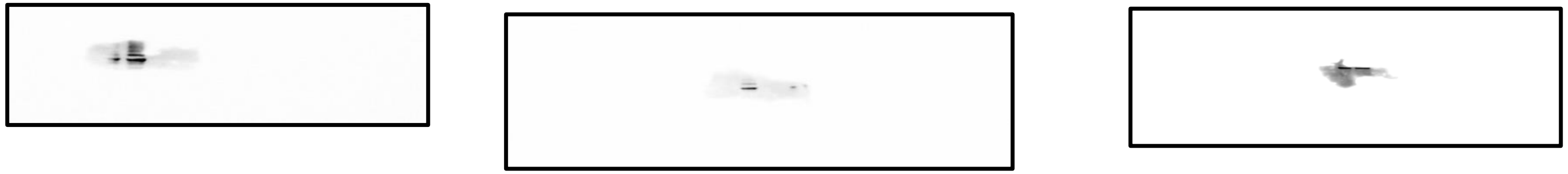

I

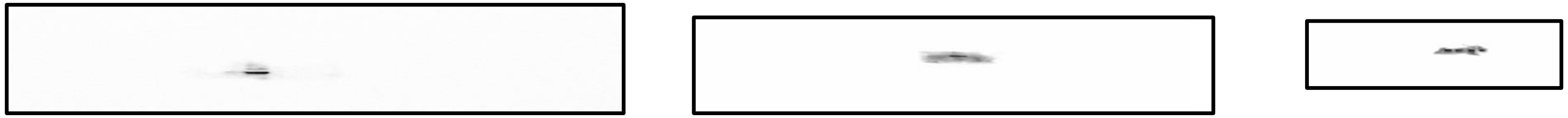

J

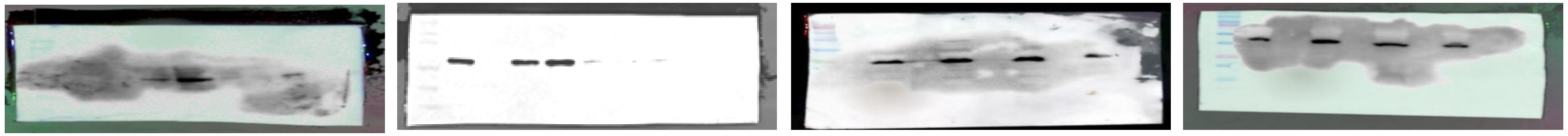

Figure 4

F

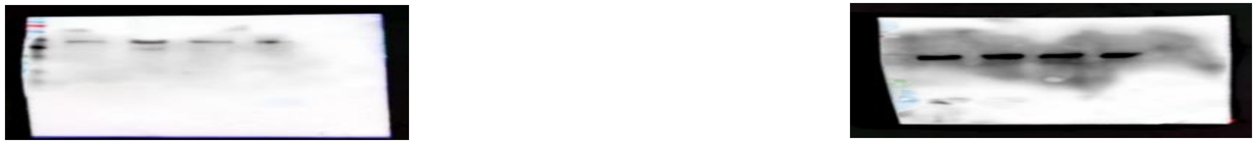

Hi

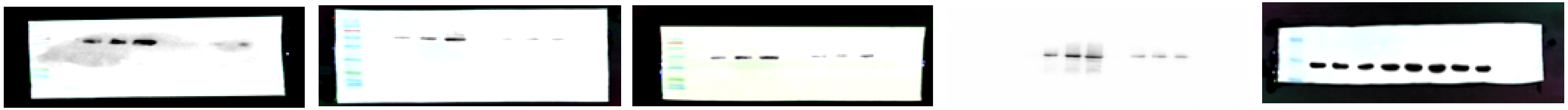

Hii

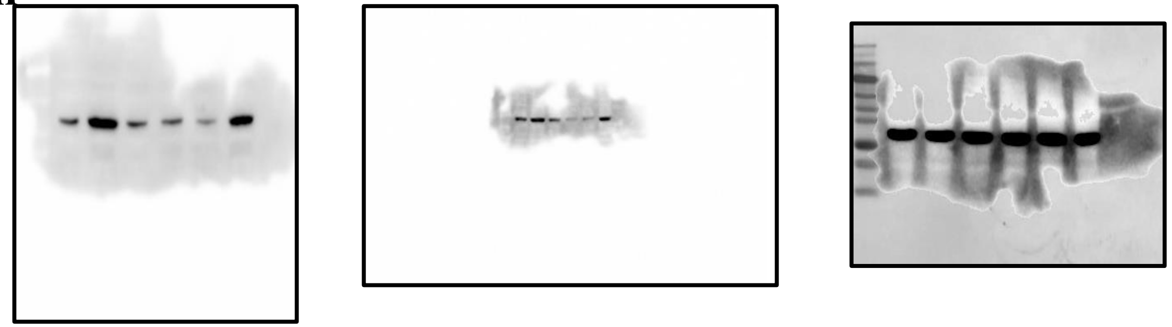

Hiii

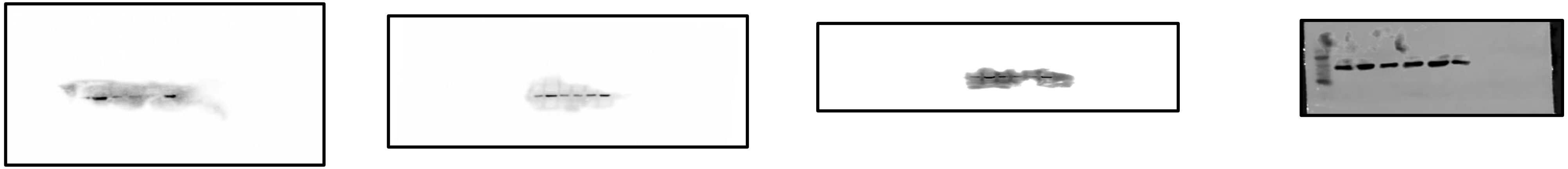

I

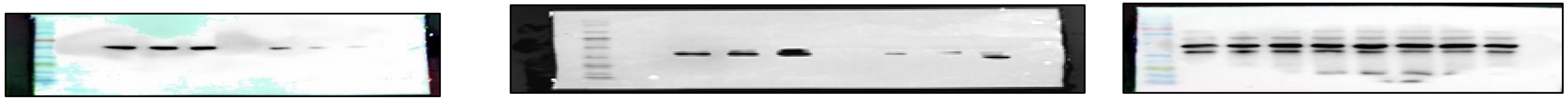

J

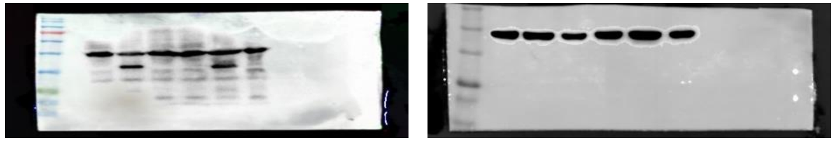

L

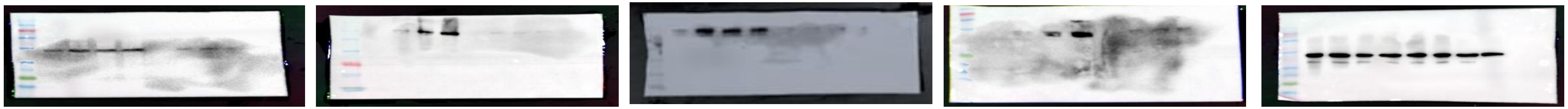

M

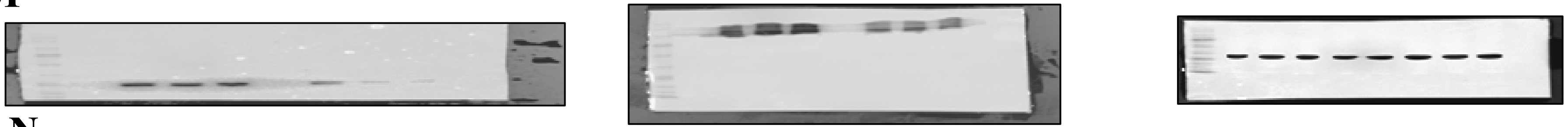

N

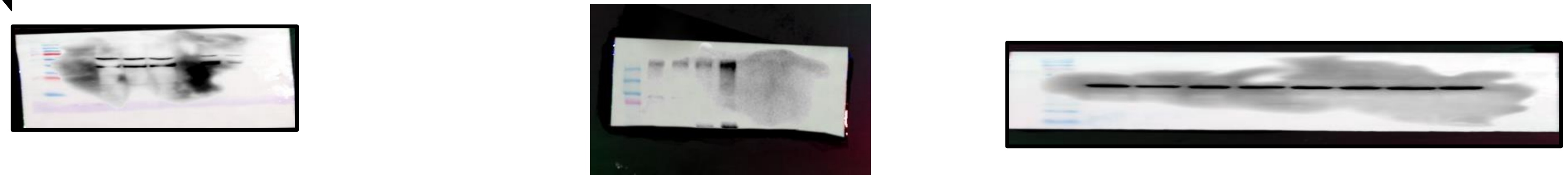

Figure 5

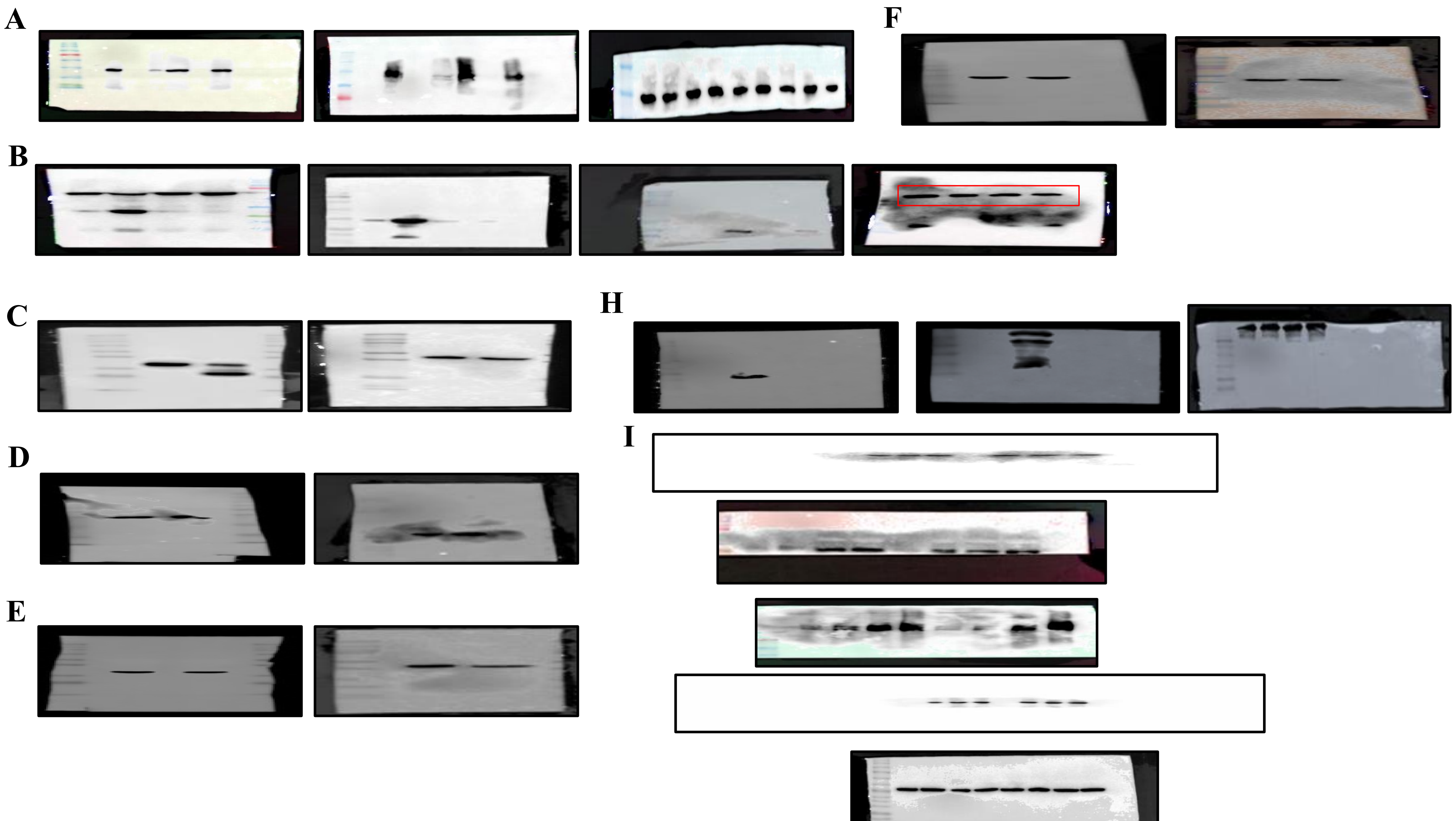

Figure 5

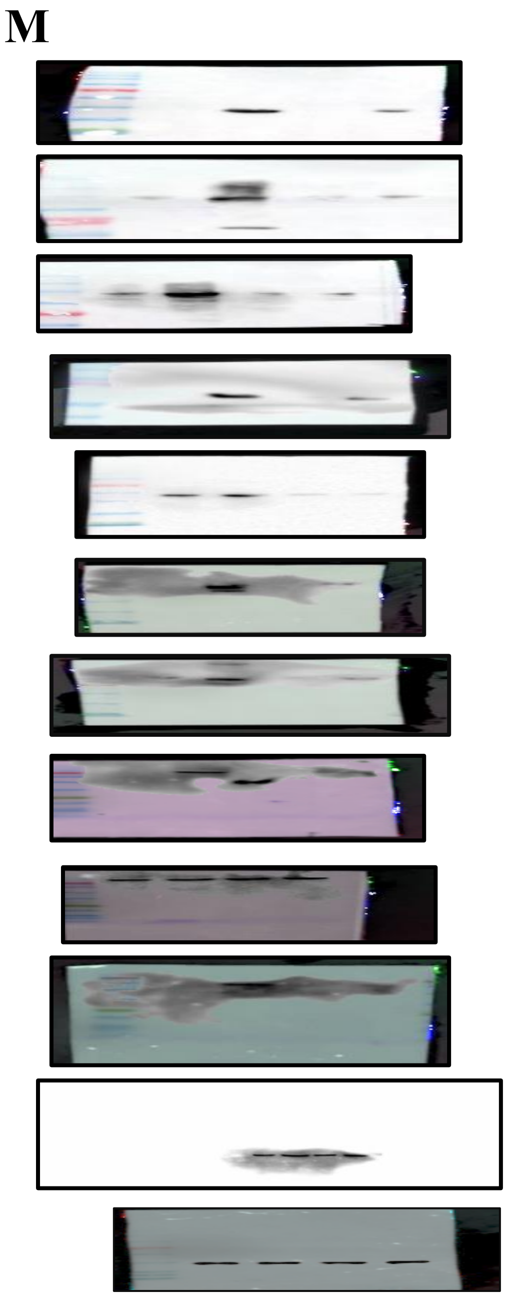

Figure 7

D

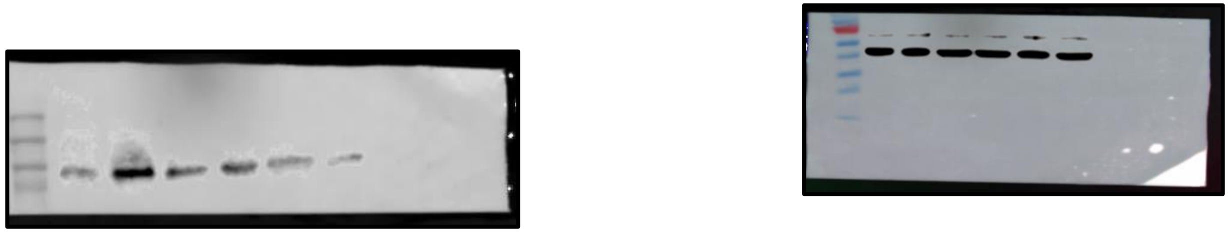

E

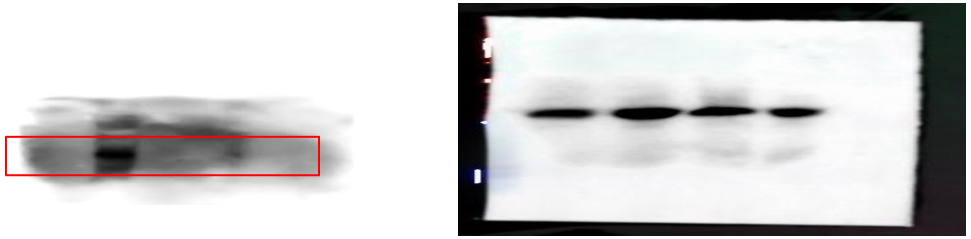

F

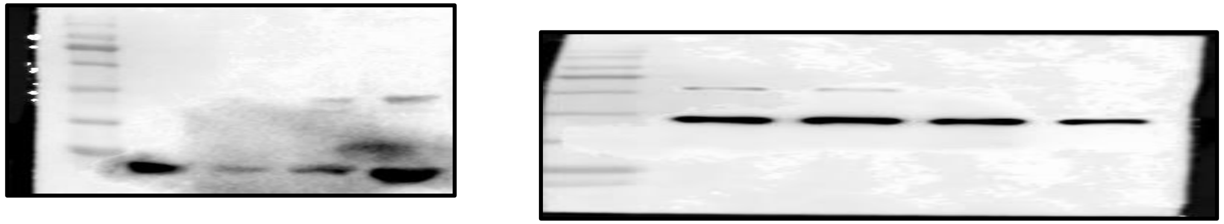

Figure S1

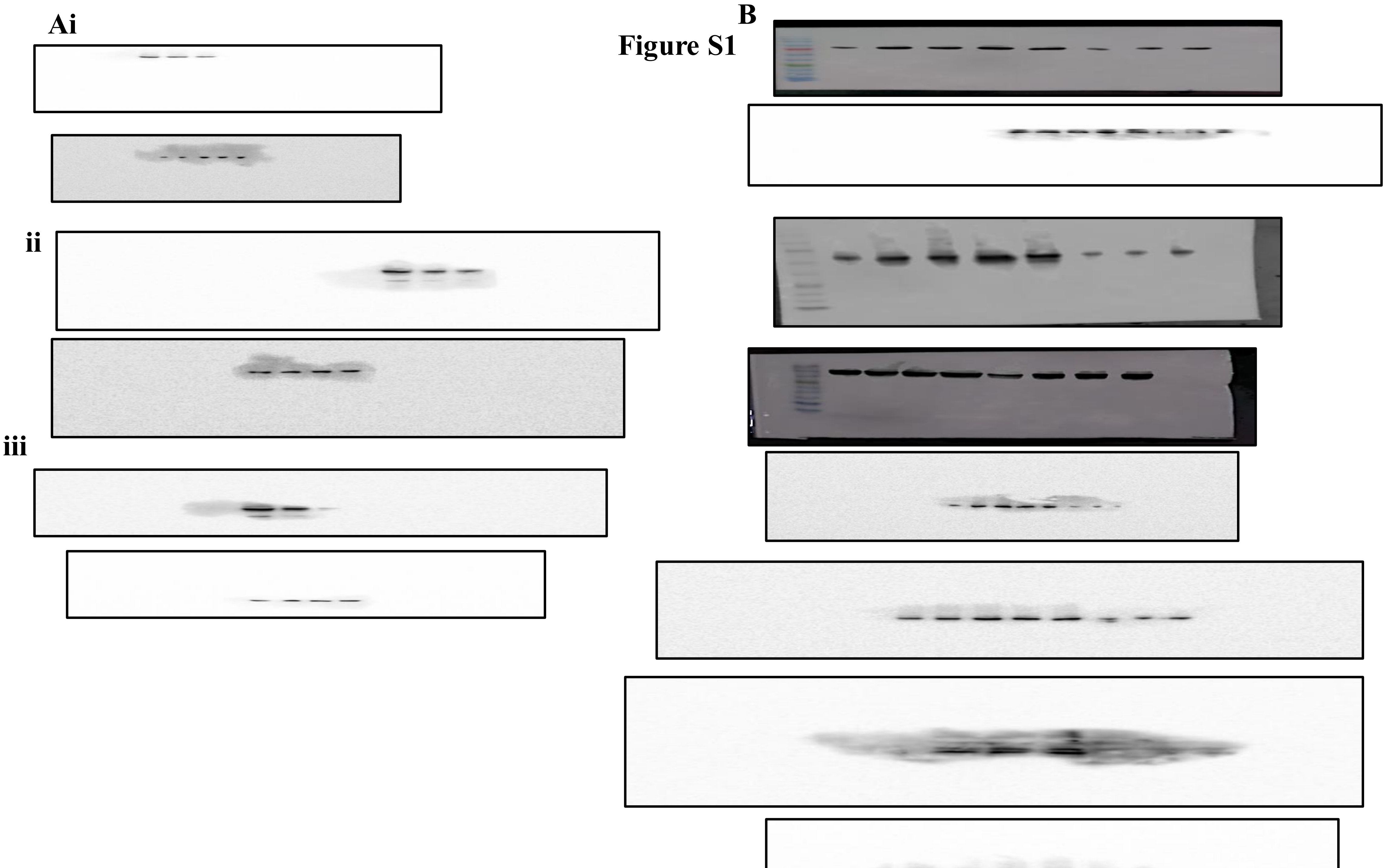

C

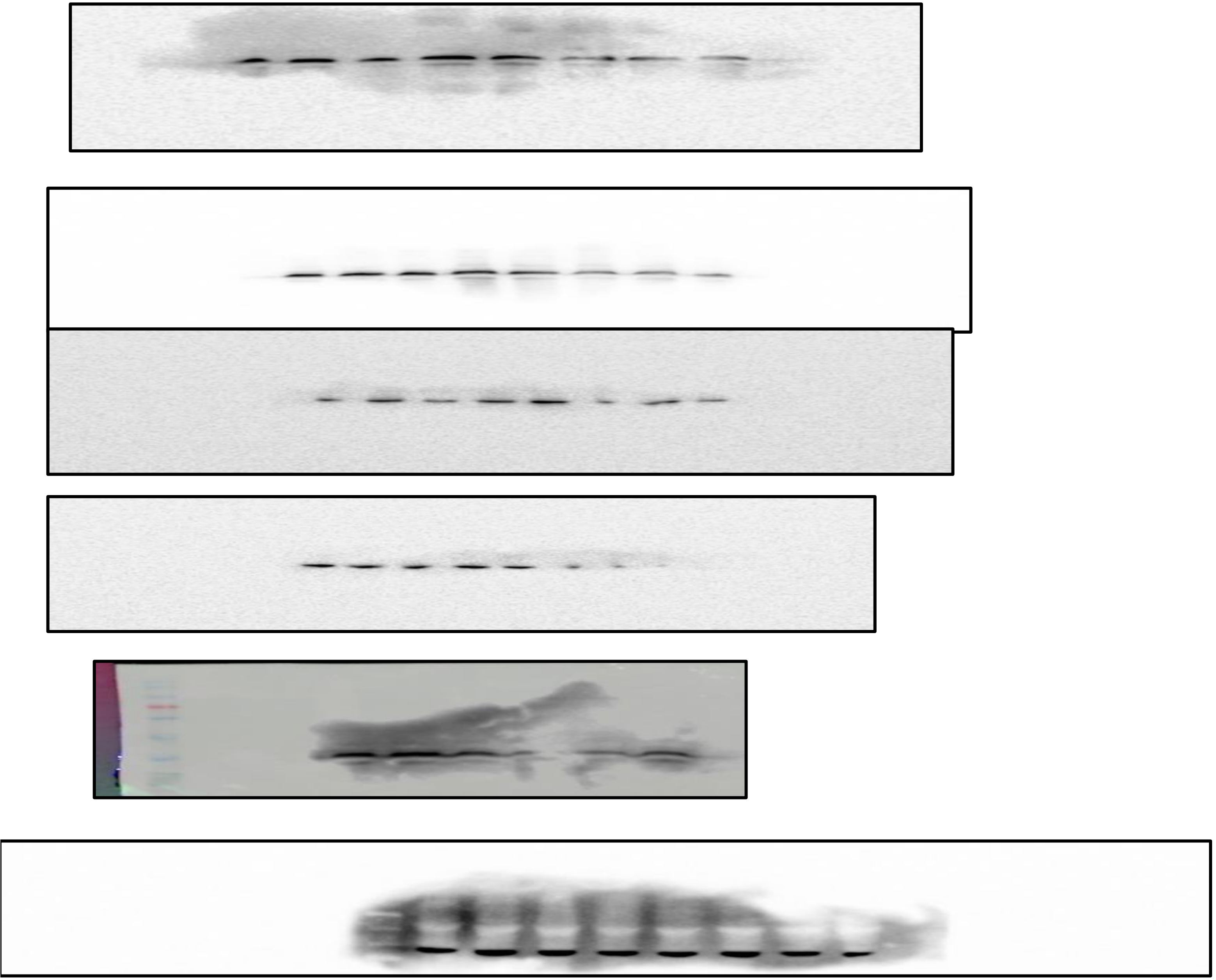

Figure S2

IV

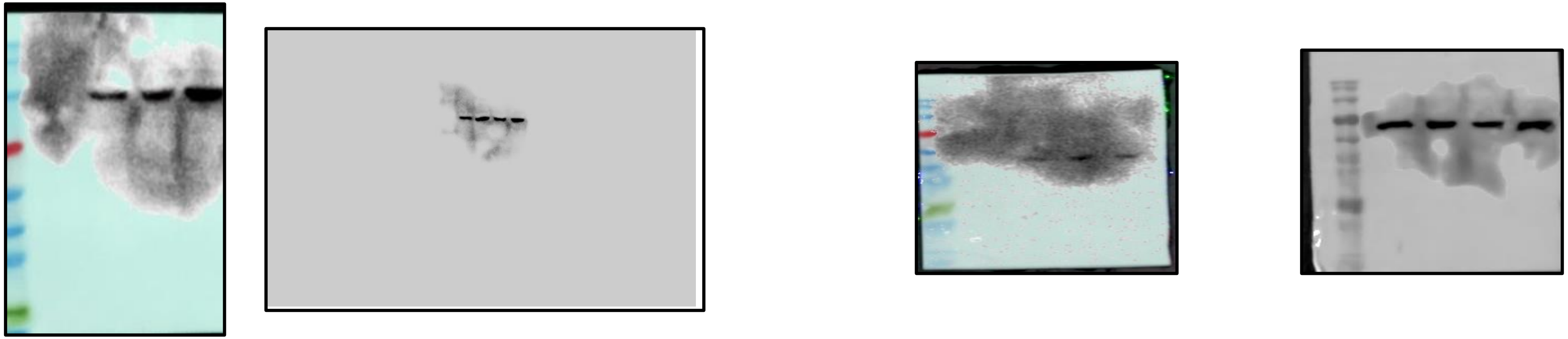

V

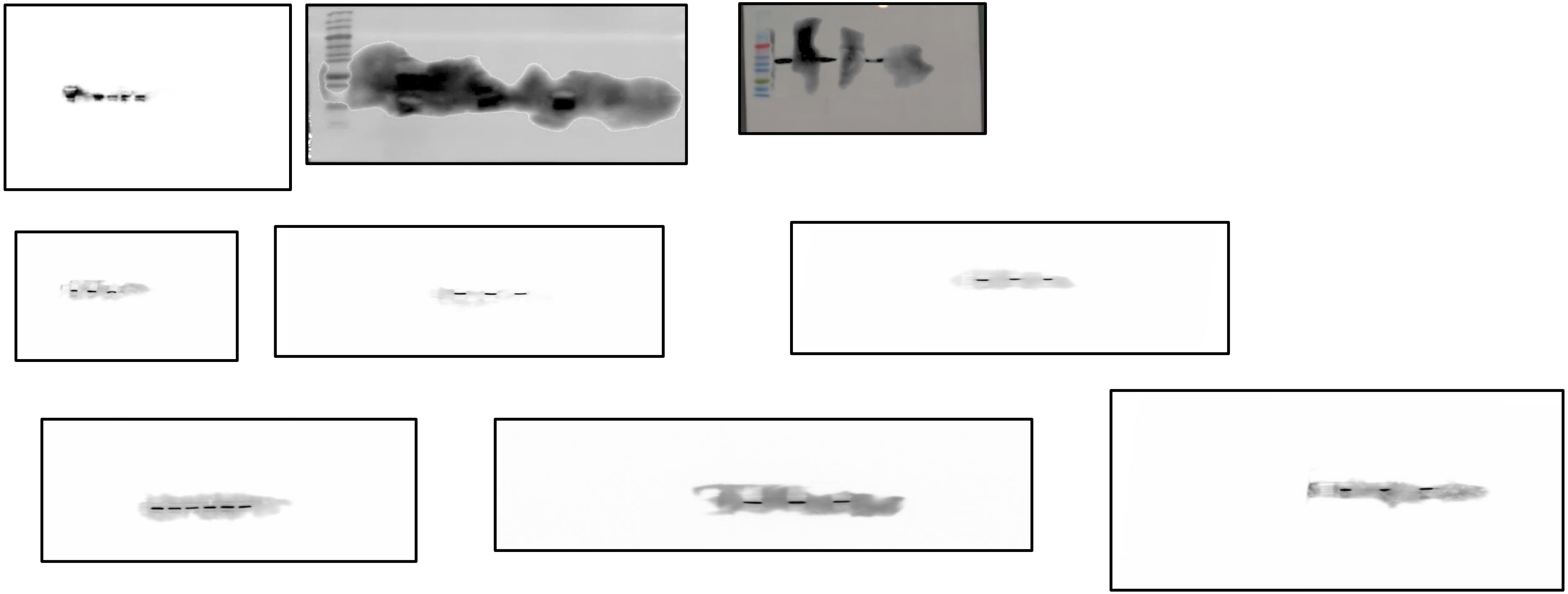

Supplement: Supplementary file 2 — original Western data [file 41419_2026_8953_MOESM2_ESM.pdf]
